# Improving Precursor Selectivity in Data-Independent Acquisition Using Overlapping Windows

**DOI:** 10.1007/s13361-018-2122-8

**Published:** 2019-01-22

**Authors:** Dario Amodei, Jarrett Egertson, Brendan X. MacLean, Richard Johnson, Gennifer E. Merrihew, Austin Keller, Don Marsh, Olga Vitek, Parag Mallick, Michael J. MacCoss

**Affiliations:** 1 Department of Radiology, Stanford University, 3155 Porter Drive, Palo Alto, CA USA; 2 Department of Genome Sciences, University of Washington, 3720 15th Ave. NE, Seattle, WA USA; 3 College of Computer and Information Science, Northeastern University, 440 Huntington Ave, Boston, MA USA

**Keywords:** Data-independent acquisition, Multiplexed acquisition, LC-MS/MS, Label-free quantification, Rapamycin, Targeted mass spectrometry, Skyline, Proteasome regulation

## Abstract

A major goal of proteomics research is the accurate and sensitive identification and quantification of a broad range of proteins within a sample. Data-independent acquisition (DIA) approaches that acquire MS/MS spectra independently of precursor information have been developed to overcome the reproducibility challenges of data-dependent acquisition and the limited breadth of targeted proteomics strategies. Typical DIA implementations use wide MS/MS isolation windows to acquire comprehensive fragment ion data. However, wide isolation windows produce highly chimeric spectra, limiting the achievable sensitivity and accuracy of quantification and identification. Here, we present a DIA strategy in which spectra are collected with overlapping (rather than adjacent or random) windows and then computationally demultiplexed. This approach improves precursor selectivity by nearly a factor of 2, without incurring any loss in mass range, mass resolution, chromatographic resolution, scan speed, or other key acquisition parameters. We demonstrate a 64% improvement in sensitivity and a 17% improvement in peptides detected in a 6-protein bovine mix spiked into a yeast background. To confirm the method’s applicability to a realistic biological experiment, we also analyze the regulation of the proteasome in yeast grown in rapamycin and show that DIA experiments with overlapping windows can help elucidate its adaptation toward the degradation of oxidatively damaged proteins. Our integrated computational and experimental DIA strategy is compatible with any DIA-capable instrument. The computational demultiplexing algorithm required to analyze the data has been made available as part of the open-source proteomics software tools Skyline and msconvert (Proteowizard), making it easy to apply as part of standard proteomics workflows.

**Electronic supplementary material:**

The online version of this article (10.1007/s13361-018-2122-8) contains supplementary material, which is available to authorized users.

**Figure:**
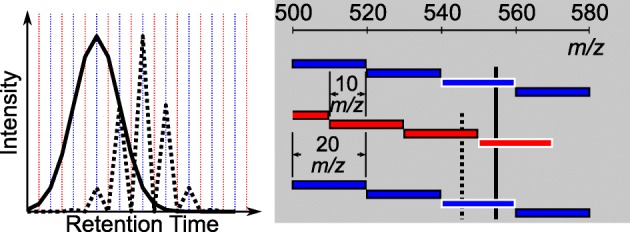
Graphical Abstract

## Introduction

Proteomics techniques for peptide detection and quantification in a complex sample typically begin with a tryptic digest of the sample of interest followed by liquid chromatography tandem mass spectrometry (LC-MS/MS). Tandem mass spectrometry (MS/MS) is almost always necessary for the unambiguous confirmation of the peptide sequence in a complex mixture [1]. Traditionally, MS/MS acquisition methods have fallen into two broad paradigms: data-dependent (or “shotgun”) acquisition (DDA) and targeted acquisition (e.g., selected reaction monitoring (SRM), parallel reaction monitoring (PRM)).

DDA is performed in two steps. First, a “survey” MS scan is performed; next, peptide precursors of interest (usually the most intense ions) are selected for fragmentation (MS/MS analysis) to determine their amino acid sequence. DDA is designed for “discovery-based” analysis, where the goal is to detect a broad range of peptides present in the sample, rather than to measure specific peptides of interest. With modern instrumentation, DDA can assess the presence of tens of thousands of peptides in a single run and is useful for the initial characterization of a sample. However, MS/MS sampling by DDA is semi-random, non-comprehensive, and most of the instrument time is spent sampling the most abundant proteins [2]. Because MS/MS sampling is essential for the unambiguous assignment of the peptide sequence, information on many peptides is absent from the acquired data. Furthermore, the only signal that is measured systematically is the MS1 precursor which is limited in selectivity and, in ion trapping instruments, the MS1 signal from a spectrum spanning a large *m*/*z* range is limited in dynamic range because of space charge limits.

Targeted acquisition, by contrast, aims to quantify a small, pre-specified set of peptides (usually 10’s or 100’s) as accurately and reproducibly as possible. SRM and PRM both cycle through highly selective analysis of pre-determined, carefully selected MS/MS acquisitions targeting the peptides of interest. Targeted precursor selection uses a narrow *m*/*z* isolation range (~ 0.2–3 *m*/*z*) which filters out signal from most irrelevant ions (e.g., chemical noise) to improve sensitivity and distinguish between co-eluting precursors. This targeted approach is extremely sensitive and selective; however, it is limited to a short list of peptides derived prior to collecting data. DDA and PRM data are acquired on the same instrumentation with similarly narrow precursor mass range isolation (0.2–3 *m*/*z*) for MS/MS scans. This narrow precursor selection produces a clean MS/MS signal and enables confident and reliable peptide identification (DDA) and quantification (PRM).

**Figure 1:**
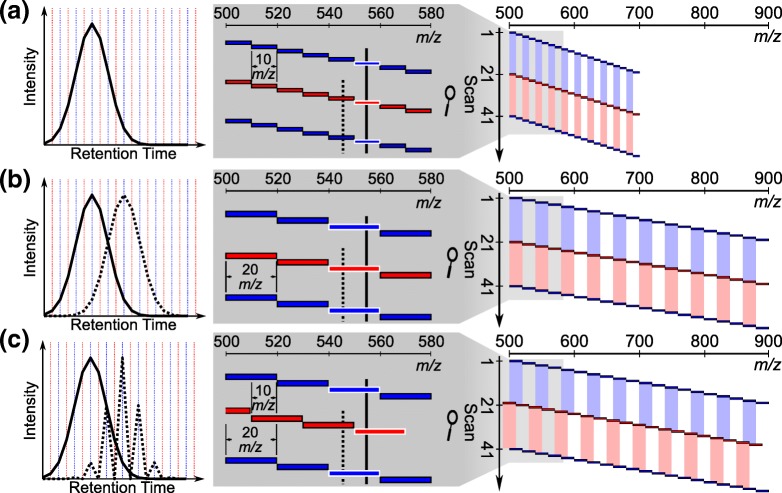
Schematic illustration of overlap acquisition schemes. (**a**) The data-independent acquisition (DIA) method covers the mass range from 500 to 700 *m*/*z* with 20 10 *m*/*z* wide targeted MS/MS scans which do not overlap with each other. (**b**) Another method also uses non-overlapping targeted MS/MS scans but with double the isolation width (20 *m*/*z*) to increase the mass range covered (500–900 *m*/*z*). (**c**) The novel “overlapped” DIA technique we have developed which repeats two sequences of scans. The first sequence (blue) is 20 consecutive non-overlapping targeted MS/MS scans (20 *m*/*z* wide isolation) covering 500–900 *m*/*z*. The second sequence (red) is the same as the first except with the windows offset to the left by − 10 *m*/*z*. The windows from the second sequence overlap with the windows from the first sequence. For all methods, the chromatograms are drawn on the left for two nearby precursors: a precursor of interest (solid black line) and an interfering precursor (dashed black line). Scans isolating the precursor of interest are outlined in white

Data-independent acquisition (DIA) [3, 4] aims to combine DDA’s strengths in discovery proteomics with the sensitivity and reproducibility of PRM. DIA often works by acquiring a repeated cycle of wide-window MS/MS scans to comprehensively sample wide portions of the *m*/*z* range [4–6]. For example, MS/MS information can be acquired for every peptide between 500 and 900 *m*/*z* by acquiring a repeated ~ 2.5-s cycle of 20 consecutive 20 *m*/*z*-isolation targeted MS/MS scans (Figure 1B) on a Thermo Q-Exactive (Thermo Fisher Scientific, Bremen, Germany). The computational analysis of DIA spectra can be performed in the same “targeted” manner as PRM data, i.e., fragment ion chromatograms for each peptide can be extracted and used for quantification. This targeted extraction can be done for any peptide in the sampled range (e.g., between 500 and 900 *m*/*z*), rather than just for a subset of pre-specified peptides. Thus, the reproducible targeting and MS/MS-based quantification of PRM can in principle be combined with DDA’s ability to identify thousands of proteins.

**Figure 2:**
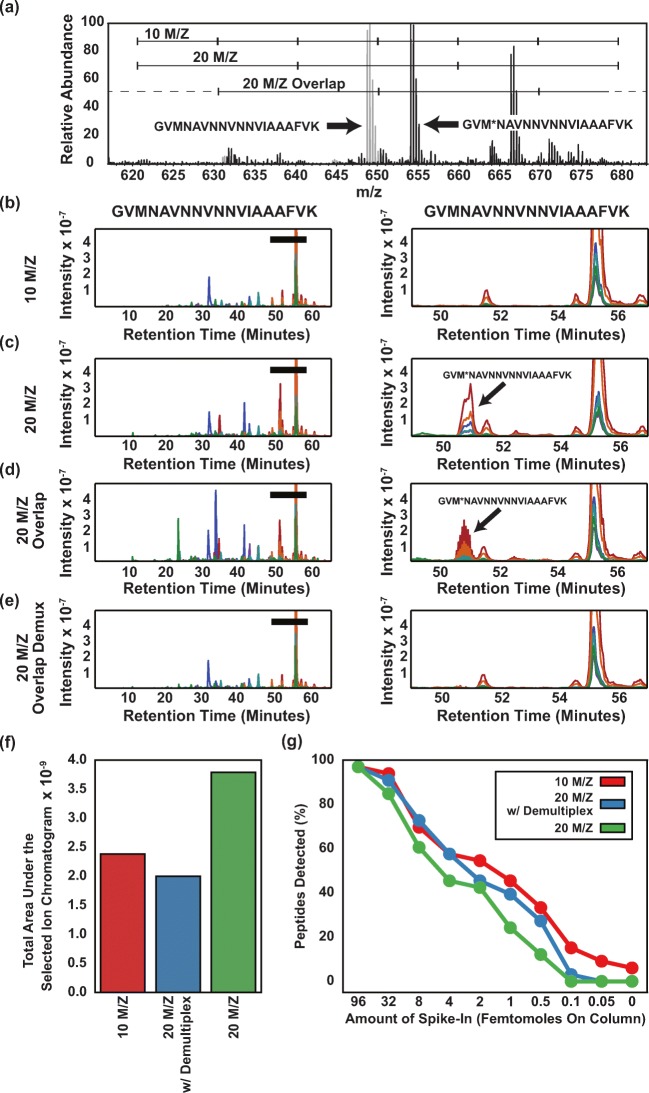
Impact of demultiplexing on precursor selectivity and detectability*.* (**a**) A representative MS spectrum showing the region where the peptide GVMNAVNNVNNVIAAAFVK+++ (light gray, at retention time 55 min) and its modified form GVM*NAVNNVNNVIAAAFVK+++ (dark gray, at retention time 51 min) are both observed. (**c**–**e**) Fragment ion chromatograms for the peptide GVMNAVNNVNNVIAAAFVK+++ were extracted from DIA data acquired on the yeast background matrix using 10 *m*/*z* windows, 20 *m*/*z* wide windows, 20 *m*/*z* wide windows with overlap, and 20 *m*/*z* wide windows with overlap and demultiplexing. The panels present a view of the full chromatograms (left) and a view zoomed in on the peak for the peptide (right). The targeted peptide elutes at ~ 55 min and a modified form with an oxidized methionine elutes at ~ 51 min. (**f**) The total area under selected ion chromatograms (a metric for precursor selectivity) for 37 peptides in the spiked-in bovine mix averaged over every spike-in amount is plotted for data acquired with 10 *m*/*z* isolation windows, 20 *m*/*z* overlapped windows with demultiplexing, and 20 *m*/*z* windows non-overlapping. (**g**) The fraction of the 32 bovine peptides analyzed in (**f**) detected directly from the DIA data using our automated peak detection algorithm at each spike-in point

One disadvantage of DIA’s large isolation width (usually greater than 10 *m*/*z* [5–7]) is that the MS/MS spectra contain product ion signals from other precursors in addition to product ions from the target precursor of interest. This poor selectivity can also arise with PRM or DDA [8]. However, precursor selectivity is much worse with DIA because the precursor selection windows are much larger. Interferences make assigning the correct chromatographic peak to the correct peptide sequence challenging. For example, using 20 *m*/*z* wide isolation windows, modified and unmodified forms of a peptide can be isolated in the same window and interfere with each other (Figure 2A). Simply reducing the isolation width of the MS/MS scans, however, negatively impacts other experimental parameters like cycle time, sensitivity, or mass range covered. One approach is to use narrow isolation windows in more complex regions of *m*/*z* and wider windows in sparser regions [9]. Increasing the resolution of fragment ion spectra can improve selectivity in a manner complementary to improved precursor selectivity but would require improvements in instrumentation or a decreased scan speed [10].

In previous work, we minimized the problem of poor precursor selectivity by using multiplexed acquisition of DIA spectra coupled with computational demultiplexing. Our multiplexing method performed the serial selection and activation and accumulation of product ions from five distinct 4-*m*/*z* precursor windows. Following the accumulation of product ions, a single mixed MS/MS spectrum was acquired. This mixed spectrum was then demultiplexed using non-negative least squares to recover the intensities of each product ion *m*/*z* belonging to the five respective precursors [11]—a technique referred to as MSX. MSX is an attractive approach because precursor selectivity can be improved without reducing the *m*/*z* range analyzed in each MS/MS scan, thus avoiding most of the tradeoffs incurred if the isolation width were reduced. The MSX method under these conditions can theoretically improve precursor selectivity fivefold (i.e., from 20 to 4 *m*/*z*). While the selectivity is improved, the method is limited by the time required to serially fill each of the precursor *m*/*z* separately resulting in a potential loss in sensitivity. Furthermore, this strategy is currently limited to quadrupole-Orbitrap mass spectrometers where the precursor isolation, activation, and accumulation can occur in parallel to the MS/MS spectrum acquisition.

Here, we present an alternative multiplexed DIA strategy that can be implemented on any quadrupole ion trap or quadrupole time-of-flight instrument. This approach improves precursor selectivity up to twofold with minimal changes to the data acquisition. The improvement in precursor selectivity is obtained by offsetting successive cycles of isolation windows with respect to each other. For example, rather than acquiring a repeating cycle of 20 by 20 *m*/*z* wide isolation windows to cover 500–900 *m*/*z*, a two-step cycle of first 20 by 20-*m*/*z* windows covering 500–900 *m*/*z* and second 20 by 20-*m*/*z* windows covering 490–890 *m*/*z* can be acquired, as in Figure 1C. The overlapping of windows makes it possible to computationally assign the proportion of each product ion’s intensity to the left or right side of a given precursor window (Figure 1C, right panel). We present a computational technique for performing the demultiplexing of overlapping precursor isolation windows, which improves precursor selectivity to 50% of the precursor window width. Because the only change to the acquisition method is shifting the location of the isolation windows in alternating scan cycles, there is no impact on the scan speed, sampling the chromatographic peak, or MS/MS spectrum quality. Below, we compare this “overlap” method to traditional approaches and note improved peptide detection and quantification (sensitivity and accuracy) on a Q-Exactive (Thermo Scientific, Bremen). The demultiplexing approach presented here should also be applicable to similar methods using overlapping window acquisition such as Waters SONAR [12], multi-mode acquisition [13], or micro-DIA [14].

We demonstrate the practical benefits of the 20-*m*/*z* overlap DIA approach by applying the method to study the response of *Saccharomyces cerevisiae* to the drug rapamycin. Rapamycin is a macrolide that robustly extends lifespan in eukaryotes ranging from yeast to mouse even if treatment only begins late in the life of the organism [15–17]. In mice, studies suggest that rapamycin appears to slow the global process of aging rather than just protecting the organism from any particular malady [18–21]. Rapamycin acts by attenuating target-of-rapamycin (TOR) signaling, causing downstream effects such as reduced protein synthesis, resistance to stress, increased autophagy, and changes in mitochondrial metabolism [16]. Using DIA, we found increased abundance of the catalytic core of the proteasome (20S subunit) and decreased abundance of the regulatory subunit (19S) in rapamycin-treated yeast. We hypothesize that the upregulation of the catalytic core by rapamycin contributes to the life-extending effect by improving the ability of the organism to degrade oxidized proteins which would otherwise unfold and form harmful protein aggregates. We performed the analysis with the 20-*m*/*z* overlapping DIA approach and a DDA approach and found the evidence of regulation was stronger and more consistent with the proposed workflow.

## Materials and Methods

The experiments in this manuscript were designed to compare the performance of our proposed combination of experimental design and computational analysis to pre-existing data-dependent and independent analytical workflows. To this end, we acquired two datasets: a spike-in experiment to measure the sensitivity, accuracy, and variability of our technique and a comparative proteomics experiment on rapamycin-treated yeast to demonstrate the practicality of the new technique. We use the term “acquisition method” throughout to refer to the scan program executed by the mass spectrometer, sometimes also referred to as the “scan sequence.” We use DDA and three DIA acquisition methods: (1) 10 *m*/*z* non-overlapping windows, (2) 20 *m*/*z* non-overlapping windows, and (3) 20 *m*/*z* overlapping windows. The term “analytical workflow” is the combination of an acquisition method and post-processing (ex. 20 *m*/*z* overlapping windows with demultiplexing). The term “condition” represents a spike-in concentration in the bovine dilution experiment and a rapamycin treatment in the rapamycin study. Details on demultiplexing of data, sample preparation, data acquisition, and data analysis follow.

### Demultiplexing of Data Acquired Using Overlapping Windows

#### Demultiplexing Algorithm

The overlap-based acquisition scheme in Figure 1C uses the pattern of the scans to infer the side of an isolation window that generated a signal. For example, a fragment that originated from a precursor *m*/*z* of 875 will appear in scans that cover 860–880 *m*/*z* and in scans that cover 870–890 *m*/*z* but *not* in scans that cover 850–870 *m*/*z*. By contrast, a fragment that originated from a precursor *m*/*z* of 865 *m*/*z* will appear in the 860–880-*m*/*z* scans and in the 850–870-*m*/*z* scans but *not* in the 870–890 *m*/*z* scan. When fragment chromatograms are extracted, signals that originated from the correct side of the 20 *m*/*z* window will appear as smooth chromatographic peaks, while signals that originated from the incorrect side will appear only in every-other scan and will have a characteristic “spiky” pattern (Figure 1C, right panel) due to their alternating presence/absence in the scans. The proposed demultiplexing algorithm conceptually exploits this property of the overlapped window scheme to remove interference and attempts to assign each signal from a 20 *m*/*z* window to its correct 10 *m*/*z* sub-window.

The algorithm is based on linear non-negative least squares and is mathematically similar to, but conceptually and practically distinct from, the algorithm for demultiplexing multifill DIA data presented previously [11]. Specifically, we define the notion of “demultiplexing regions,” which are the smallest windows to which signals can mathematically be assigned based on the pattern of overlapping windows. For example, in the acquisition strategies used in this manuscript (see “Data-Independent Acquisition”), the demultiplexing regions have a width of 10 *m*/*z*. Each 20 *m*/*z* isolation window combines signals from two demultiplexing regions, the left and the right halves of the window (see Figure 1C). There are a total of 41 demultiplexing regions, in steps of 10 *m*/*z* from 490 to 900 *m*/*z*. Our goal is to assign signals of interest in a given isolation window to the correct demultiplexing region.

To do this, for each scan *s* and for each transition of interest, we use the scans *s* − 20 to *s* + 19 to form a linear system of equations relating the measured intensity of each scan to the combination of multiplexed regions that it includes. We then solve the matrix equation:

$$ XA=Y $$

where *X* is an *n* × *n* “design matrix” whose rows represent scans (plus one regularization row) and whose columns represent demultiplexing regions (for our data *n* = 41) and contains a 1 wherever a scan includes a given demultiplexing region and a 0 where it does not. *Y* is the “observed data,” an *n* × *m* matrix whose rows represent scans (plus one regularization row) and whose columns represent fragment ions (*m* is the number of fragment ions of interest and depends on the specific peptides being analyzed) and whose entries represent the observed intensity of each transition for each scan. *A* is an *n* × *m* matrix whose rows represent demultiplexing regions and whose columns represent transitions and whose entries represent the unknown demultiplexing region intensities that we are solving for.

Because mass spectrometry signal intensities are required to be positive, we solve the equation *XA = Y* using non-negative least squares, specifically using the Lawson-Hanson algorithm [22]. This technique is similar to the method described previously, but unlike the randomly chosen, non-adjacent windows described previously [11], for our overlapping windows, each MS/MS scan consists of adjacent demultiplexing regions, so the matrix *A* is close to diagonal. This allows us to use special-purpose heuristics to approximate the solution of the constrained least squares problem quickly. An approximate solution can be obtained by working with small blocks of the matrix equation *XA = Y* rather than having to work with the full matrices (see Supplementary Note). With these heuristics, we can demultiplex ~ 1000 transitions from ~ 56,000 MS/MS spectra in 10 min. The mathematical details of solving the matrix equation are described in the Supplementary Note.

The non-negative least squares algorithm, including our heuristic approximations, is general and thus can be applied to any acquisition scheme that involves partially overlapping windows. The algorithm is not limited to the 20 *m*/*z* width/10 *m*/*z* overlap chosen in this manuscript. The algorithm is implemented in Skyline [23] (see Supplementary Methods) and ProteoWizard msconvert [24] where it can be used to produce full demultiplexed spectra.

### Bovine Spike-In Experiment: Data Acquisition

#### Sample Preparation

We generated a series of samples in which an equimolar six bovine protein digest (Bruker-Michrom) was spiked into a background matrix of digested *S. cerevisiae* soluble lysate. The yeast tryptic digest background was prepared from 400 μL of 5.1 μg/μL of yeast proteins (strain BY4741) that are soluble in 50 mM ammonium bicarbonate (ABC). This digest was combined with 60 μL 1 M ABC, 110 μL 0.5% PPS detergent (Agilent Technologies, Santa Clara, CA), and 6 μL 500 mM Tris(2-carboxyethyl)phosphine (Thermo Fisher Scientific, Rockford, IL) for 30 min at 60 °C. The reaction was cooled to room temperature and 8 μL 500 mM iodoacetamide was allowed to react for 30 min prior to the addition of 40 μL of 2 μg/μL TPCK-treated trypsin (Worthington Biochemical, Lakewood, NJ). Digestion proceeded for 4 h at 37 °C. Residual iodoacetamide was quenched with 5 μL 500 mM dithiothreitol for 15 min at 37 °C. The final digest was acidified with 60 μL 10% trifluoroacetic acid. The six bovine protein tryptic peptide mixture (Bruker, Auburn, CA) was mixed in various concentrations (48, 16, 4, 2, 1, 0.5, 0.25, 0.05, and 0.025 fmol/μL) with a constant yeast tryptic peptide background (0.75 μg/μL). Two microliters of each sample was injected for each mass spectrometry run.

#### Liquid Chromatography

Splitless, nano-flow liquid chromatography separation was performed using a Waters nanoACQUITY (Waters Corp., Milford, MA) UPLC system configured for partial loop injection coupled online to the Q-Exactive mass spectrometer. Fused silica capillary tubing (75 μm I.D.; Polymicro Technologies) was pulled to a tip of ~ 5 μm at one end and packed with 15 cm of Jupiter Proteo 4 μm C12 beads (Phenomenex, Torrance, CA). A Kasil fritted trap was prepared using a 100-μm I.D. capillary tube packed with 2 cm of the identical C12 packing material, and 2 μL of sample was trapped at a flow rate of 1 μL/min for 8 min prior to going in-line with the packed tip. Peptides were separated over a 60-min linear reversed phase gradient ranging from 2 to 32% Buffer B (0.1% *w*/*v* formic acid in acetonitrile) mixed with Buffer A (0.1% *w*/*v* formic acid in water) with a flow rate of 300 nL/min. Quality control runs analyzed a six bovine protein digest (Bruker-Michrom, Auburn, CA) using a 30-min linear gradient.

#### Q-Exactive

The capillary temperature was set to 300 °C. The S-Lens RF level was set to 50.0. The instrument was running the Q-Exactive 2.2 SP1 software.

#### Data-Independent Acquisition

Three methods were used for DIA acquisition: 10, 20, and 20 *m*/*z* with overlap. The 10 *m*/*z* method was a repeating cycle of twenty 10 *m*/*z* wide windows, covering the range from 500 to 700 *m*/*z*, as shown in Figure 1A. The 20 *m*/*z* method was a repeating cycle of twenty 20 *m*/*z* wide windows, covering the range from 500 to 900 *m*/*z*, as shown in Figure 1B. The 20 *m*/*z* overlap method also used twenty 20 *m*/*z* wide windows, but alternating cycles were offset by − 10 *m*/*z*, so that odd-numbered cycles covered windows from 500 to 900 *m*/*z*, while even-numbered cycles covered windows from 490 to 890 *m*/*z* (Figure 1C). All isolation window locations were optimized to place the edges into regions where peptide precursor ions are unlikely to occur [11]. In all methods, MS/MS DIA scans were taken at a resolving power (R.P.) of 17,500 at 200 *m*/*z*, a maximum fill time of 55 ms, an AGC target of 1 million ions, a default charge state of 2, and a normalized collision energy of 20.0. In all three methods, an MS survey scan was taken every 10 MS/MS scans, with R.P. 35,000 at 200 *m*/*z*, a maximum fill time of 55 ms, and an AGC target of 1 million ions. The scan range of the MS scan was 500–700 *m*/*z* for the 10 *m*/*z* method and 500–900 *m*/*z* for the 20 *m*/*z* methods. Data for both MS and MS/MS scans were acquired in profile mode.

#### Data-Dependent Acquisition

MS scans were acquired with R.P. 35,000 at 200 *m*/*z*, an AGC target of 1 million ions, and a maximum inject time of 10 ms in profile mode covering the range from 400 to 2000 *m*/*z*. MS/MS scans were acquired with R.P. 17,500 at 200 *m*/*z*, an AGC target of 200,000 ions, a maximum injection time of 55 ms, and a normalized collision energy of 23 in profile mode. The DDA method was a top-12 method, targeting the top 12 most abundant precursors for MS/MS after every MS scan. Unassigned and singly charged peptides were excluded from selection for MS/MS analysis. Dynamic exclusion was on with an exclusion time of 15 s and the “Exclude isotopes” feature enabled.

#### Experiment Design

Each sample was analyzed using four acquisition methods—one DDA and three DIA (10, 20, and 20 *m*/*z* overlap, see “Data-Independent Acquisition” above). The DDA runs were necessary to construct a spectral library for validation of peptide identifications from DIA data. In total, 120 runs were performed (4 analytical workflows, 3 biological replicates, 10 dilutions) excluding blanks and quality control (QC) runs. The runs were block-randomized. Each of the replicate sets consisted of all the spiked concentrations and acquisition methods. The three replicate sets were acquired sequentially. Within each replicate set, the concentrations were run in an increasing order, to avoid carryover effects. Within each concentration, the order of acquisition methods was randomized in order to avoid batch effects. A QC and two blank runs were conducted every 10 injections. A full run sheet of the experiment is shown in Supplementary Table 1. One of the three technical replicates of the 4-fmol concentration in the 20 *m*/*z* acquisition method was truncated at 55 min, causing 11 peptides to receive no measurement in that replicate. These peptides were excluded from the analysis (see “Selection of Peptides and Precursors”).

### Bovine Spike-In Experiment: Data Analysis

We used the bovine spike-in data to compare acquisition workflows based on metrics pertaining to selectivity, peptide detection, and peptide quantification. We used the automated mProphet [25] algorithm to compare the quality of peptide detection across the three DIA workflows. mProphet automatically selects the best chromatographic peak for each query peptide sequence (from the six spiked-in bovine proteins) and assigns a *q* value indicating the confidence in that peak annotation. We used the number of spike-in peptides detected by mProphet below a 1% false discovery rate cutoff as a metric for comparing peptide detection quality between different analytical workflows.

When comparing peptide quantification, we made efforts to avoid confounding the comparison with differences in peptide detection quality between each DIA workflow. To this end, we verified that the chromatographic peak selected for each peptide was within 2 min of its library DDA spectrum.

#### Selection of Peptides and Precursors

The three DDA runs at the highest spike-in concentration (96 fmol) were searched against all of these tryptic peptides using X!Tandem [26] and PeptideProphet [27] in CPAS [28]. Peptides were selected if they were identified at least once at PeptideProphet score > 0.95. Peptides that were outside of the analyzed *m*/*z* range (500–700 or 500–900 *m*/*z*), less than 6 or greater than 25 amino acids, or contained missed cleavages were filtered from the analysis. There were 37 such peptides in the 500–700 *m*/*z* range and 53 in the complete 500–900 *m*/*z* range. A complete list of these peptides is shown in Supplementary Table 3. Eleven of the detected peptides were removed from the analysis because they had retention time > 55 min and were thus truncated in the 20 *m*/*z* 4-fmol file (see “Bovine Spike-In Experiment—Experiment Design”).

Fragment ion extraction for these peptides was performed in Skyline, with demultiplexing applied on-the-fly in the case of overlapping window methods (see Supplementary Methods and Supplementary Note).

#### Full Extracted Ion Current Analysis

To compare the precursor selectivity of the analytical workflows used in this manuscript, we analyzed the integrated intensity across the entire retention time profile of all extracted chromatograms (fragment ions) of interest, in each of the acquisition strategies. Raw chromatograms were exported from Skyline and the TotalArea column (which represents intensity integrated over time without background subtraction across the entire run for each fragment) was summed over all fragment ions and then averaged over peptides and replicates within each acquisition strategy. The resulting number (reported in the main results) represents the total intensity (integrated area) recorded at all fragment masses of interest for a given peptide, from zero retention time to the end of the run and is thus a measure of how selective the extracted chromatogram is for the desired peptide (fewer interfering ions means more selectivity). For this analysis, the single acquisition replicate that was truncated (20 *m*/*z* at 4 fmol) was removed, and all 37 peptides between 500 and 700 *m*/*z* were retained in the analysis (see “Selection of Peptides and Precursors”).

#### Peptide Detection

For comparisons of peptide detection across acquisition methods, we used the Skyline implementation of the mProphet algorithm [25] with the peptides (and associated DDA spectra) described in the section “Selection of Peptides and Precursors.” Spectral libraries were constructed from the three DDA runs at the highest spike-in concentration. The top 8 transitions from each library spectrum in Skyline were used as input transition peak groups to the feature scores. Decoy peptides were generated from reversed peptide sequences. Unless otherwise noted, only peaks within 5 min of the DDA spectrum retention time were considered by mProphet. The features used in the prediction were as follows: “Intensity,” “Retention time difference,” “Retention time difference squared,” “Library intensity dot-product,” “Shape (weighted),” “Co-elution (weighted),” “Co-elution count,” “Signal to noise,” “Product mass error,” “Precursor-product shape score,” “Precursor mass error,” and “Precursor isotope dot product.” A false discovery rate threshold of *q* < 0.01 was used for detection.

#### Peptide Quantification and Limit of Quantification Analysis

We quantified and analyzed the lower limit of quantification (LoQ) of the spike-in dilution series at both the fragment and the peptide precursor ion level. Integrated peak areas were calculated by Skyline using peak boundaries chosen by a combination of mProphet and linear retention time alignment (see “Peak Picking for Quantification”). Peak areas in each LC-MS/MS run were normalized by integrated peak areas of a set of peptides from the background yeast matrix to correct for run-to-run variation in, e.g., LC column performance, ion source cleanliness, and sample load volume (see “Peak Area Normalization”). MSStats version 3.10.2 was used to calculate the lower limit of quantification from spike-in curve data based on the shape of the spike-in curve and reproducibility of replicate measurements [29].

Results at the peptide level were calculated by summing over the integrated areas of the top *N* most intense fragment ions from the DDA library spectra. Unless otherwise indicated, the top 5 most intense fragment ions were used. Only transitions from the *y* or *b*-ion fragment ion series at index 3 to *n* − 2 with charge + 1 or + 2 and *m*/*z* < 1500 were considered. To determine the average LoQ of an acquisition method across many peptides, we took the geometric mean of the LoQ of each peptide because the LoQ may vary over several orders of magnitude between peptides. Bias was quantified for each peptide with each acquisition method as follows:

1. A calibration factor *D* between observed intensity (*I*) and true peptide concentration is calculated from the top 2 spike-in concentrations (32 and 96 fmol) and the mean of the intensities measured for the peptide at that intensity point:

$$ D=\frac{1}{2}\times \left(\frac{96}{\mathrm{Mean}\left({I}_{96}\right)}+\frac{32}{\mathrm{Mean}\left({I}_{32}\right)}\right) $$

2. For any measurement with intensity *I* and true peptide concentration (*C*), the bias is then calculated as the absolute value of the log2 fold change between the “measured” concentration calculated from the intensities (*D* × *I*) and the true concentration *C*:

$$ \mathrm{Bias}\ \mathrm{at}\ C=\left|{\log}_2\left(\frac{D\times I}{C}\right)\right| $$

Variability was quantified as the coefficient of variation of the intensity measurements across replicates. The bias and variability of peptide measurements were only calculated at concentrations that were above a fixed concentration cutoff for each peptide. The fixed concentration cutoff for each peptide was the maximum LLOQ observed for that peptide among the acquisition techniques being compared.

MS1-based quantification was accomplished by using the interlaced MS1 scans acquired once every 10 MS/MS scans (see “Experiment Design”) in the DIA data. We performed MS1 quantification using the sum of the integrated areas under the *M*, *M* + 1, and *M* + 2 isotope peaks.

#### Peak Picking for Quantification

Peak boundaries for the bovine spike-in experiment were calculated using an algorithm to propagate peak integration boundaries from high spike-in concentration runs to lower concentration runs where the peptide may not be detected. First, unrefined peak boundaries were estimated by using the boundaries from an mProphet (see “Peptide Detection”) analysis in Skyline keeping only boundaries from detections with *q* < 0.01. Next, peak boundaries from runs where the peptide was detected are propagated to runs where the peptide was not detected. Boundaries are propagated using an alignment function generated from a weighted spline interpolation on common yeast background detections (see “Peak Area Normalization”) between a run where the peptide was detected and the one in which it was not. Finally, the peak boundaries are adjusted to a fixed width such that the width of each integrated peak in retention time is consistent between all measurements for a given peptide in an acquisition mode (i.e., 10, 20, or 20 *m*/*z* + overlap). To accomplish this, the desired integration width is calculated as the maximum width observed for the peptide at the 96-fmol spike-in point. Additionally, a “reference spectrum” is calculated for the peptide by averaging the measured transition intensities at 96 fmol. For each run, the best location for the boundaries with the desired width is calculated by performing a local search around the existing boundaries (calculated by mProphet or propagation from mProphet results). The local search considers all peak boundary locations centered within ± 0.1 min of the existing location and calculates the optimal location as the one with the best least squares fit between the reference spectrum and the observed transition intensities at the potential center retention time. These peak boundaries are then imported back into the Skyline document.

#### Peak Area Normalization

DDA runs acquired on the yeast background matrix with no spike-in were used to identify peptides that could be used for normalization. The LC-MS/MS runs were database searched using the Crux [30] pipeline with Comet [31] version 2015.01 rev2 and Percolator [32] version 2.09. The identified peptides were first filtered down to those with precursor charge 2 and precursor *m*/*z* between 500 and 700. The peak intensity was calculated for each of the candidate normalization peptides using Skyline [23]. For each peptide, the transitions used for quantification were the top 8 fragment ions measured in the reference peptide-spectrum match from the DDA data. The area under the curve of each of these transitions was extracted from the 10 *m*/*z* wide window DIA data acquired on the yeast background (no spike-in) and summed to represent the signal from each peptide. Only the top 100 most intense peptides were kept as candidate normalization peptides.

Peak areas for each of these 100 peptides were extracted from every LC-MS/MS run in the bovine spike-in experiment using Skyline using the top 5 most intense transitions. For each analytical workflow, each normalization peptide was manually investigated and removed if it demonstrated poor signal, chemical noise interference, poor chromatography, or a potentially incorrect peak assignment in any of the LC-MS/MS runs. The 10, 20, and 20 *m*/*z* overlapping (with demux) and 20 *m*/*z* overlapping (without demux) workflows used 84, 81, 84, and 86 peptides for normalization, respectively.

For each LC-MS/MS run (*i*), the total normalization intensity (*N*) is calculated by summing up the integrated peak area of each normalization peptide (*k*):

$$ {N}_i={\sum}_{k=1}^n{Area}_{i,k} $$

where *n* is the number of peptides used for normalization in LC-MS/MS run *i*. Each run *i* has an associated analytical workflow:

$$ Analytical\ workflow(i)\in \left\{10\  mz,20\  mz,20\  mz\  OverlapDemux,20\  mz\  OverlapNoDemux\right\} $$

For each analytical workflow (*a*), the top normalization intensity (TNI) is calculated as:

$$ {TNI}_a=\max \left({N}_i\  where\ AnalyticalWorkflow(i)=a\right) $$

A normalization factor (NF) is calculated for each run as:

$$ {NF}_i=\frac{TNI_a}{N_i}\ \mathrm{where}\ a= AnalyticalWorkflow(i) $$

The normalized peak area for a given peptide (*k*) measured in run *i* is then calculated as:

$$ {NormalizedArea}_{i,k}={NF}_i\times {Area}_{i,k} $$

### Rapamycin Experiment: Data Acquisition

The quality control runs, liquid chromatography setup, Q-Exactive setup, and data acquisition methods were the same for this experiment as in the Bovine Spike-In Experiment (see “Bovine Spike-In Experiment: Data Acquisition”).

#### Sample Preparation

Three conditions were tested in this experiment: wild-type yeast, wild-type yeast grown in rapamycin, and *tor1* deletion mutant yeast. The wild-type and *tor1* haploid strains of *S. cerevisiae* were from an ORF deletion collection [33] and had the parental background BY4742—MATα hist3∆1 leu2∆0 lys2∆0 ura3∆0. Both strains were grown in YPD media in three biological replicates. The wild-type strain was grown either in the presence or absence of 10 nM rapamycin. Strains were grown from OD600 ~ 0.15 to OD600 0.6 prior to lysis and digestion. The samples without rapamycin grew to this density in about 6 h, while the rapamycin-treated strains required 8 h. Cells were lysed by bead beating using a lysis buffer of 50 mM ammonium bicarbonate at pH 7.8 with phosphatase inhibitors (Pierce—Halt phosphatase inhibitor cocktail). Bead beating was for 1 min, followed by 1 min of cooling the sample on ice, repeated three times. The digestion was performed for 1 h using sequencing-grade trypsin (Promega, Madison, WI) as in Hoopmann et al. [34] except using 0.1% PPS Silent Surfactant (Protein Discoveries) instead of RapiGest SF (Waters Corporation, Milford, MA).

#### Experiment Design

Three conditions (WT, WT + rapamycin, *tor1*) were run with three biological replicates each. For each of these runs, three data acquisition methods were run (20 *m*/*z* overlap, 10 *m*/*z*, and DDA). The runs were block-randomized. Each replicate set consisted of all the measurements on a biological replicate. The order of the conditions was randomized, and then the order of three acquisition methods was further randomized within each condition. The order of the runs is provided in Supplementary Table 2. QC and blank runs were performed every three instrument runs. The chromatography and mass spectrometry methods were as described above.

### Rapamycin Experiment: Data Analysis

#### DDA Analysis and Pathway Query

The DDA data was searched using a pipeline which runs Bullseye [35], Sequest [36], and Percolator [32] to identify peptides. Bullseye was run to determine high accuracy precursor *m*/*z* values (~ 10 ppm or less) prior to the database search by using MS1 data and peak detection. SEQUEST was run using a semi-tryptic search against a yeast open reading frame database (target) obtained from the Saccharomyces Genome Database that was built on February 3, 2011. The data were also searched against a reversed (decoy) database. The searches were done with a precursor peptide mass tolerance of 10 ppm and fragment ion mass tolerance of 0.03 *m*/*z* using monoisotopic parent and fragment ion masses and allowing for two missed cleavages. Percolator was used to calculate peptide level *q* values for every peptide. A total of 8607 unique peptides were identified with *q* < 0.01 in all the data sets combined.

Sixty-four proteins involved in protein ubiquitination, assembly of the 26S proteasome, and the proteasome itself were chosen as targets for analysis (see Supplementary Table 4). Of these 64 proteins, 32 had at least one confident peptide identification (*q* < 0.01) in the DDA data. Peak picking and MSStats analysis were performed on any protein with at least one confident peptide identification. Special considerations did not need to be taken for propeptides of the beta subunits (1, 2, 5, 6, and 7) because none were identified. The original set of query proteins and the number of peptides identified for each can be found in Supplementary Table 4. The number of peptides used for quantifying each protein is overlaid on the structure of the proteasome in Supplementary Figure 1.

#### Peak Picking and Statistical Analysis

For each data acquisition method (10 *m*/*z*, 20 *m*/*z* with overlap, DDA MS1 filtering), Skyline was used to extract chromatograms for each peptide of interest. For the DDA data, the *M*, *M* + 1, and *M* + 2 precursor ion chromatograms were extracted for each peptide. For DIA data, subsets of the singly-charged *b* and *y*-ion series were extracted ranging from the third ion in each series to the *N* − 2 ion where *N* is the length of the series. *M*, *M* + 1, and *M* + 2 precursor ion chromatograms were also extracted from the DIA data but were not used unless otherwise indicated. Peaks were selected by manually mapping confident DDA identifications to each dataset using retention time, precursor ion mass accuracy, and relative fragment ion intensity information. The peak boundaries were selected by manual integration. For fragment ion refinement in the DIA methods, the top 5 most intense fragment ion peaks from the DDA library spectra were used. The total intensity for each run was normalized by the MS1 TIC within each acquisition method. Using MSstats [37], the peak areas for the peptides were then used to find proteins that had significantly increased or decreased abundance in the WT vs. WT + rapamycin comparison or the WT vs. *tor1* comparison. The statistical model was generated in MSStats v1.99 beta using the label-free setting. The model included a term to model interference (feature x condition). The model also accounted for heterogeneous intensity variation between features. The scope of biological replication was set to “restricted,” and the scope of technical replication was set to “expanded.” The default action for missing values was “no interaction.” MSStats was run separately for each data acquisition type and *p* values adjusted for multiple hypothesis testing were reported.

#### Data and Tutorial Availability

All RAW datafiles and Skyline documents associated with this manuscript can be found at https://panoramaweb.org/DIAOverlap.url on Panorama Public and via http://www.proteomexchange.org/ (PXD011910). Additionally, a tutorial for how to generate the Q-Exactive overlapping window isolation list can be found at https://skyline.ms/tip-dia-overlap.url. A tutorial for how to perform full spectrum demultiplexing of overlapped DIA windows using msconvert can be found at https://skyline.ms/tip-dia-demux.url.

## Results

Here we introduce a DIA acquisition strategy that incorporates halfway overlapping 20 *m*/*z* DIA acquisition windows (Figure 1C) with a sophisticated spectral demultiplexing algorithm. Using the intermittent pattern of scans (such as in the right panel of Figure 1C), the approach infers the half of an isolation window that originated a fragment ion signal. Then, fragment ion chromatograms containing signal from only a single 10 *m*/*z* sub-window are generated using computational demultiplexing (see “Demultiplexing Algorithm” in Methods).

We evaluated the performance of this new acquisition workflow against a standard 20 *m*/*z* workflow (Figure 1B) and a standard 10 *m*/*z* workflow (Figure 1A). The standard 20 *m*/*z* workflow serves as a negative control that allows a direct measurement of the improvements in precursor selectivity (the proportion of ions in the scan that come from the peptide of interest as opposed to other interfering peptides or noise) due to overlapping windows. The standard 10 *m*/*z* method serves as a positive control and is intended to represent the level of precursor selectivity that could be theoretically achieved if the overlapping of 20 *m*/*z* windows were fully successful in removing half of the interference in each window. However, the 10 *m*/*z* method covers only half the mass range of the 20 *m*/*z* methods and therefore lacks a key advantage of the 20 *m*/*z* methods which is not reflected in these comparisons. Below, we assess the gains in precursor selectivity, identification, and quantification of the overlap DIA strategy by benchmarking it against these other strategies.

### Overlapped Windows Increase Precursor Selectivity for Spiked-In Peptides

We first tested whether the overlap-based workflow achieves precursor selectivity superior to that of 20 *m*/*z* windows and comparable to that of 10 *m*/*z* windows. To qualitatively illustrate the improvement in precursor selectivity by overlapping windows, we present extracted chromatograms for the peptide GVMNAVNNVNNVIAAAFVK+++, taken from the yeast background of the bovine spike-in dataset with no bovine digest spiked-in (Figure 2). This peptide had an abundant, modified form (oxidized methionine) that eluted at ~ 51 min. When chromatograms were extracted from the 10 *m*/*z* DIA data, the modified form did not appear because the modified and unmodified forms were in different windows (Figure 2B). However, when the 20 *m*/*z* DIA data were used, the modified form of the peptide appeared alongside the unmodified form (Figure 2C). By contrast when overlap was used without demultiplexing, the modified form was partially removed and showed a characteristic spiky pattern (Figure 2D) due to their alternating presence/absence in the scans. Using overlapping windows with computational demultiplexing, the modified form of the peptide virtually disappeared (Figure 2E), making the unmodified form more unambiguously identifiable. The overlap demultiplexing removed many spurious peaks from the full chromatogram (Figure 2E, left panel) compared to data acquired without overlap (Figure 2C, left panel), making the true peak more apparent.

To quantify this effect, we computed the total integrated area (see “Full Extracted Ion Current Analysis” in Methods) across the entire elution profile from all extracted fragment chromatograms of each peptide in the bovine spike-in dataset. This gave an indication of the total amount of ion current extracted using each acquisition methodology, with the assumption that most of the ion current will be from chemical noise which will be in part removed by the demultiplexing algorithm. We found average (across peptides) extracted total intensity of 2.4 × 10^9^ ions/s for the 10 *m*/*z* windows, 3.8 × 10^9^ ions/s for the 20 *m*/*z* windows, and 2.0 × 10^9^ ions/s for the 20 *m*/*z* windows with overlap and demultiplexing (Figure 2F). These data support the claim that the 20 *m*/*z* overlap method with demultiplexing has similar precursor selectivity to the 10 *m*/*z* method and roughly twice the precursor selectivity of the 20 *m*/*z* non-overlapped method.

### Overlapped Windows Increase Peptide Detections for Spiked-In Peptides

The improvements in precursor selectivity from overlapping windows and demultiplexing should translate into improvements in peptide detectability due to the removal of spurious peaks. We compared the detection of 32 spiked-in peptides (see “Peptide Detection” in Methods) in the 500–700 *m*/*z* range from the bovine spike-in dilution series using feature scores derived from the mProphet algorithm (see “Peptide Detection” in Methods) across acquisition workflows. We found that the 10 *m*/*z* acquisition workflow detected 411 peptides (not unique) across all concentrations, 28% more than the standard 20 *m*/*z* workflow, which detected 321 peptides. Overlapping the windows together with computational demultiplexing (376 peptides) led to a 17% improvement in peptide detections compared to the 20 *m*/*z* workflow (Figure 2G). The greatest increases in detection benefits were seen at the 1-fmol and to some extent 0.5-fmol concentrations. This may have been because at these concentrations many peptides were just below their limit of detection using the standard 20 *m*/*z* workflow but were detectable using the 20 *m*/*z* overlap workflow due to improved precursor selectivity. Overall, we conclude that the overlapping window workflow produces a substantial and robust improvement in detected peptides relative to standard 20 *m*/*z* windows.

### Overlapped Windows Improves Sensitivity of Quantification for Spiked-In Peptides

**Figure 3:**
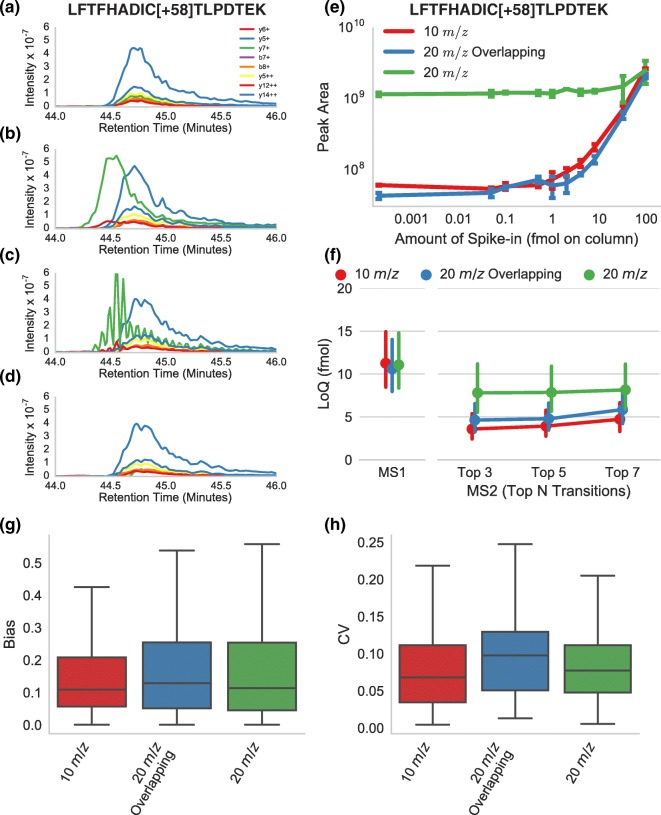
Impact of demultiplexing on quantification. **a**–**d** The top 8 fragment ion (according to a DDA library spectrum) chromatograms for the bovine peptide LFTFHADIC[+58]TLPDTEK+++ in the bovine protein digest, spiked into background yeast matrix at amounts ranging from 50 amol to 96 fmol. The fragment ion chromatograms for this peptide extracted from the DIA spectra acquired with 10 *m/z* windows, 20 *m/z* wide windows, 20 *m/z* wide windows with overlap, and 20 *m/z* wide windows with overlap and demultiplexing. **e** The measured intensity (summed area under the curve for the extracted transitions) of the peptide at each spike-in point for multiple acquisition techniques. The arithmetic mean and standard deviation (error bars) of three replicate measurements are plotted. **f** The lower limit of quantitation averaged (geometric mean) over 32 bovine peptides is shown when quantitation is done using either MS1 data acquired as part of the 10 *m/z* DIA acquisition or DIA data integrating all transitions or the top *N* most intense transitions from a library spectrum. Error bars indicate the 68% confidence interval on the geometric mean calculated by bootstrapping. **g**, **h** The accuracy and the variability of each method over the 32 bovine peptides calculated across replicate peak areas of the top 5 transitions. Whiskers indicate the most extreme observation within 1.5 times the interquartile range above or below the upper and lower quartile, respectively

We expect the improvement in precursor selectivity from overlapping windows to translate into improved quantitation due to the removal of chemical noise interference. To qualitatively demonstrate this effect, we show extracted chromatograms for the top 8 transitions (according to a DDA MS/MS spectrum) for the bovine spike-in peptide LFTFHADIC[+58]TLPDTEK+++. When chromatograms for the peptide were extracted using the 20 *m*/*z* DIA non-overlapped method, there was a strong interference in the y7^+^ ion which was not present when the data were extracted from the 10 *m*/*z* (non-overlapped) data (Figure 3A, B). In the 20 *m*/*z* overlapping window data, this same interference appeared but was removed by demultiplexing (Figure 3C, D). Qualitatively, small distortions were still visible at the apex and right side of the peak, but the demultiplexed chromatograms came very close to matching the quality of the 10 *m*/*z* chromatograms. The removal of interference by overlapping windows and demultiplexing improved the lower limit of quantitation (LLoQ) for this peptide from > 96 fmol (20 *m*/*z* no overlap) to 3.6 fmol (20 *m*/*z* overlap demultiplexed) which nearly matched the LLoQ from the 10 *m*/*z* DIA data (2.3 fmol) (Figure 3E) when the top 5 most intense transitions are integrated for quantification.

This improvement in sensitivity is generalizable to the 32 peptides of interest from the bovine spike-in dilution series. We compared the average LLoQ of all 32 peptides and found that 20 *m*/*z* with overlap demultiplexed (4.78 fmol) outperformed the standard 20 *m*/*z* method (7.85 fmol) by 64% and performed moderately worse than the 10 *m*/*z* method (3.92 fmol) (Figure 3F) when peptides were quantified by summing the top 5 transitions. The relative performance of the acquisition methods was not significantly impacted by the number of transitions summed for each peptide. We additionally calculated the average LLoQ of the peptides when quantified using *M*, *M* + 1, and *M* + 2 precursor ion chromatograms extracted from MS1 scans acquired after every 10 MS/MS scans in the 10 *m*/*z* DIA data and found that MS1 was less sensitive than DIA. In addition to the peptide level results, we measured limit of quantification on a transition-by-transition basis and found similar results (Supplementary Figure 3).

Limit of quantification is not the only important figure of merit. Accuracy and variability (both at low and high concentration) are also important, because correctly detecting fold changes at medium to high abundance can be just as critical as detecting a low abundance protein. To measure accuracy, we calculated the deviation of the measured peptide level from its expected value (see “Materials and Methods”) and found that the 20 *m*/*z* overlap approach did not decrease accuracy. When computing accuracy, only measurements above the lower limit of quantification for all three acquisition methods were compared.

To measure reproducibility, we computed the CV of the peak area across replicates for each acquisition method (Figure 3H) for measurements above the lower limit of quantification for all three acquisition methods. The mean CV over the 32 peptides measured was 0.09 for 10 *m*/*z*, 0.11 for 20 *m*/*z*, and 0.11 for the overlap technique. While not statistically significant, it appears that acquiring data with overlapping acquisition windows may cause a minor decrease in measurement reproducibility. The reproducibility of overlapped 20 *m*/*z* data with and without demultiplexing is similar (Supplementary Figure 4), indicating that the reduction in reproducibility may be due to the overlapped acquisition method itself rather than the computational demultiplexing.

These results were generalized to the wider 500–900 *m*/*z* range, of the 20 *m*/*z* isolation scheme and the overlap scheme (see Supplementary Results and Discussion, Supplementary Figure 4).

### DIA Detects Changes in the Yeast Proteasome in Response to Growth in Rapamycin

Wild-type (WT) *S. cerevisiae* yeast grown with and without rapamycin were analyzed using 20 *m*/*z* overlap DIA and DDA to demonstrate the utility of the overlap method for biological discovery and compare it to a DDA-based approach. Sixty-four proteins involved in proteasome-mediated degradation were selected as targets for the analysis. This set of proteins included the 26S proteasome, proteins involved in assembling the proteasome, and ubiquitinating proteins. Of these 64 proteins, 32 had at least one peptide identified by DDA using an automated database search (see “Materials and Methods”). Information for comparative quantification of these proteins in the three treatments were extracted from both the DDA [38] and DIA data using Skyline and analyzed using MSStats [39] (Supplementary Tables 6 and 7 for DDA, and DIA, respectively) to identify proteins that had significantly increased or decreased abundance (adjusted *p* value < 0.05). Very few significant changes were found when comparing the wild-type samples to the *tor1* mutant samples, so only results from the rapamycin vs. WT comparison are presented here.

**Figure 4:**
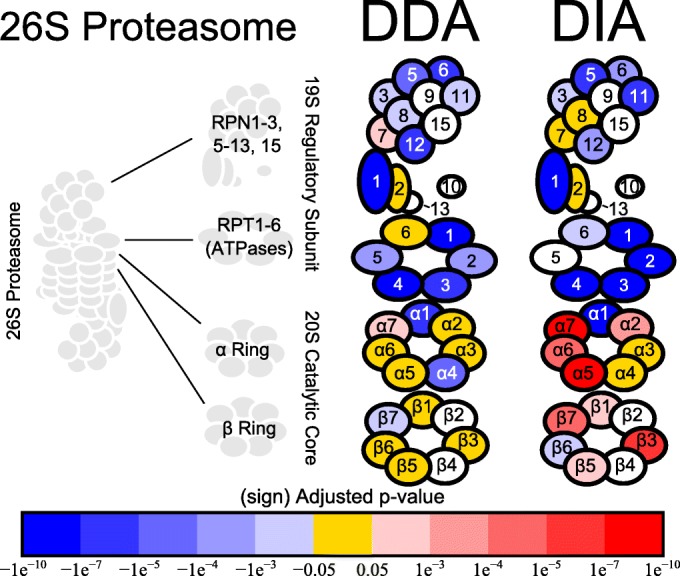
Changes in proteasome abundance detected using 20 *m*/*z* overlapped DIA. A cartoon structure of the yeast 26S proteasome is depicted (left). The proteasome is built from the 20S catalytic core, which consists of two β-rings sandwiched between two α-rings, with a 19S regulatory subunit attached on the top and bottom. The cartoons on the right show the proteins of the regulatory subunit and catalytic core with a color overlaid depicting proteins not detected (white), detected with no change in abundance (gold), significantly decreased abundance (blue), or significantly increased abundance (red). The *p* values used to determine significance were generated using MSstats. The numbers overlaid on the proteins of the regulatory subunit indicate which member of the RPN/RPT family each protein is. The protein names are overlaid on the proteins of the 20S catalytic core

Both the DDA and DIA (20 *m*/*z* overlap) workflows detected a decreased abundance of the regulatory subunit of the proteasome in response to rapamycin (Figure 4). The decrease in abundance was especially apparent for the RPT proteins, ATPases which unfold proteins as they are fed into the catalytic core. Five of the six RPT proteins were detected and all were found to have decreased abundance by DIA. The decrease in abundance of the regulatory subunit could mean a decrease in ubiquitin-mediated protein degradation as the regulatory subunit is responsible for targeting poly-ubiquitinated substrates to the proteasome.

The DIA data showed evidence of increased abundance of the catalytic core, with 8 of the 12 detected catalytic core proteins having increased abundance. An increase in abundance after rapamycin treatment was detected for two of the three proteolytically active proteins of the catalytic core (β1 and β5). The increase in the abundance of the catalytic core could mean an increase in the capacity of the cell to degrade oxidatively damaged proteins as the catalytic core can do so more efficiently than the intact 26S proteasome.

An important difference between the DDA and DIA data was the *m*/*z* range analyzed. The DDA data analyzed the range from 400 to 2000 *m*/*z*, while the DIA data analyzed the range from 500 to 900 *m*/*z* which impacted the number of peptides that were analyzed for each protein. For the proteins analyzed in Figure 4, DDA had an average of 2.2 peptides per protein while DIA averaged 1.8 peptides per protein (Supplementary Figure 1). Although the DDA technique analyzed four times the *m*/*z* range of DIA, the number of peptides per protein on average was still similar because the range between *m*/*z* 500 and 900 is very rich in peptides resulting from tryptic digestion [40, 41]. A more detailed discussion on the impact of the difference in the *m*/*z* region analyzed can be found in the Supplement.

Proteins outside of the proteasome itself were also queried but are not shown in Figure 4. Both ubiquitin (UBA1) and the yeast E1 ubiquitin ligase UBI4 were found to have decreased abundance in response to rapamycin treatment. Thirty-two of the 64 proteins queried were not identified in the sample. RPN4 is a transcription factor which binds upstream of genes encoding for the proteasome which was not identified in our data. Only one (UBC1) of the 11 E2 ubiquitin-conjugating enzymes was detected, but no changes in abundance were found. Additionally, none of the chaperones involved in the assembly of the proteasome, the proteasome catalytic core activator BLM10, nor the deubiquitinating enzymes involved in deubiquitination (UBP6, RPN11, DOA4, or HUL5) were identified. The full list of queried proteins can be found in Supplementary Table 4. The DIA workflow produced more consistent changes in abundance across subunits of the proteasome despite that fact that using DIA, most subunit members were quantified with fewer peptides than using DDA. This points to the utility of the proposed workflow for biological discovery.

## Discussion

By shifting the isolation center of MS/MS scans in every other scan cycle, many popular implementations of DIA such as FT-ARM and SWATH stand to see substantial benefits in selectivity and sensitivity with virtually no negative tradeoffs. By overlapping isolation windows and demultiplexing the data prior to analysis, we improve sensitivity by 64% and peptide detection by 17% in an analysis of a bovine mixture spiked into yeast on a Q-Exactive. The workflow is applicable to any DIA-capable instrument and can be implemented immediately with the tools required for analysis (namely the demultiplexing algorithm) already available as part of the Skyline software package. For these improvements, there are very minimal tradeoffs in the *m*/*z* range covered (~ 3%), and the demultiplexing may require additional processing time when information on many peptides is extracted.

We emphasize that our acquisition workflow is not limited, in principle or in practice, to 20 *m*/*z* windows, overlaps that cover half of a window, or alternating pairs of duty cycles. The basic linear demultiplexing method could be applied to windows that overlap by a third or even to acquisition schemes that step across overlapping windows successively rather than by alternating duty cycles. Examples include Waters SONAR [12] and micro-DIA [14] data. Our demultiplexing implementation in Skyline has been written to support arbitrarily arranged windows, though schemes other than the one presented in this paper may not be as well-optimized for speed. We use 20 *m*/*z* wide isolation windows for this study because they allow coverage of a wide *m*/*z* range (500–900 *m*/*z*) with a duty cycle (~ 2.5 s) fast enough to enable a frequent peptide sampling and an accurate reconstruction of MS/MS chromatograms for quantification. The optimal scan parameters (i.e., window width, number of windows, etc.) are machine-, sample-, and chromatography-dependent [10]. The magnitude of the benefit from overlapping windows will depend on the complexity of the sample being analyzed. When analyzing complex samples, improved precursor selectivity should substantially reduce the complexity of the resulting MS/MS spectra. However, in less complex samples, even MS/MS spectra acquired with wide isolation windows may not contain many components and thus not benefit from the improved precursor selectivity of overlapping windows.

Our method has some potential limitations. Perhaps the most significant is that it does not always perfectly remove interferences. The main reason for this imperfection is that the linear system of equations used to assign signals to sub-windows assumes that two nearby scans (e.g., a 500–520 *m*/*z* scan and a 510–530 *m*/*z* scan on the next cycle) covering a sub-window (e.g., 510–520 *m*/*z*) will read exactly the same intensity for that sub-window, whereas in reality, the intensity will change due to the shape of the elution profile. We attempt to correct for this error by smoothing and interpolating the entries of the linear equation to track the shape of the elution peak, but this correction technique is not perfect and some inaccuracy will remain. This inaccuracy will lead to residual errors in the demultiplexed solution, including failure to fully remove interfering peaks. Normally these errors will be small, but if an interference is several orders of magnitude larger than the desired signal, then the residual error could still be large enough relative to the signal to distort peak quantification or identification. We expect this limitation to be more severe when elution profiles are narrow and/or windows cycles are long.

A second limitation of our workflow is that, while our overlapping 20 *m*/*z* windows can achieve the selectivity of 10 *m*/*z* windows, they still face the intra-scan dynamic range issues associated with 20 *m*/*z* windows. That is, despite computationally removing interfering species from half of the 20 *m*/*z* window, we still physically collect all the ions from the full window. These issues may partly explain why quantification, while improved relative to standard 20 *m*/*z* windows, still fell slightly short of 10 *m*/*z* windows.

Finally, we note that the reproducibility of the overlapping window approach may be slightly worse than a non-overlapped approach. We suggest that this could be due to non-uniformity in isolation across a wide isolation window. As previously demonstrated [11], the quadrupole mass filter on the Q-Exactive does not isolate precursor ions with the same efficiency at all *m*/*z* locations of a wide isolation window. Precursors with *m*/*z* nearing the edges of the isolation window are transmitted with lower efficiency than in the center. This non-uniformity in isolation should affect reproducibility of non-overlapping DIA approaches if the precursor is isolated near the edges of the isolation window. However, overlapping approaches are additionally affected due to the systematic sampling of a single peptide at two different locations of the isolation window throughout acquisition. We hypothesize that this reproducibility issue will be less pronounced or eliminated on more recent hardware (for example, the QE-Plus or QE-HF) with more uniform quadrupole isolation.

None of these limitations, except in extreme cases, are expected to produce worse performance than a comparable 20 *m*/*z* DIA workflow without overlap. We emphasize that the 20 *m*/*z* workflow with overlap is identical to a 20 *m*/*z* workflow without overlap except for the shifted position of half of the window cycles. Overlapping the isolation windows provides an improvement in precursor selectivity “for free,” without affecting experimental setup or results in any other way. The limitations described above, however, should determine whether precursor selectivity is improved to fully match that of a 10 *m*/*z* acquisition scheme covering half the total *m*/*z* range or whether the improvement falls short of this benchmark. Our data suggests a nearly ideal benefit for analysis in a yeast matrix.

The proposed workflow is useful for biological discovery. Here we have applied our acquisition technique and processing pipeline to the study of the response of yeast cells to growth in rapamycin and discovered evidence of increased abundance of the 20S proteasomal component in yeast cells grown in rapamycin. This observation suggests that rapamycin compensates for the age-associated decline of the ability of the cell to deal with oxidative stress. Organisms that rely on cellular respiration must be able to cope with the ongoing oxidative stress caused by mitochondrial metabolism which converts 1–2% of oxygen into the superoxide ion radical, a reactive oxygen species (ROS) [42]. ROS damage proteins and lipids in the cell despite the presence of antioxidant enzymes such as superoxide dismutase [43]. The 20S proteasome is the primary proteasomal mechanism cells use to degrade oxidatively damaged proteins [44–47]. Not only is it more efficient at degrading oxidatively damaged proteins than the 26S proteasome, it is also more resistant to inactivation by oxidative damage [48, 49]. As a cell ages, the mitochondria produce more ROS and the proteasomal degradation system is impaired [50–52]. Our results suggest that rapamycin may compensate for these changes by upregulating the core 20S proteasome components, thus increasing the cell’s capacity to degrade oxidatively damaged proteins before they form harmful aggregates, although further studies will be necessary to validate this claim.

The decrease in the abundance of ubiquitin, the yeast E1 ubiquitin ligase UBI1, and the 19S regulatory particle seems to indicate that there is less ubiquitin-mediated degradation occurring in the cells grown in rapamycin. Unfortunately, our technique was only sensitive enough to measure 1 of the 11 ubiquitin-conjugating enzymes present in the yeast cell. Additionally, none of the molecular chaperones which help assemble the proteasome were detected nor were proteasome regulators such as RPN4 and BLM10. Even with the improved sensitivity of our DIA method, entire groups of proteins are beyond the limit of detection. This is a caveat in virtually any proteomic study due to the large dynamic range and high complexity of the unenriched proteome.

In conclusion, we demonstrate a simple modification of standard DIA approaches, which leads to notable improvements in peptide detection and quantification when coupled with a novel and widely available demultiplexing algorithm implemented in Skyline and Proteowizard msconvert. Using the technique, we detect meaningful changes in the yeast proteasome in response to growth in rapamycin which could impact aging research pending more in-depth studies. The overlap technique coupled with demultiplexing should be applicable on any mass spectrometer capable of performing DIA acquisition, including FT-ICR, Orbitrap, quadrupole ion traps, and time-of-flight instruments. Finally, as discussed above, a wide variety of schemes based on different arrangements of overlapping windows may be possible. We expect that this versatile, general, and easy to implement technique coupled with demultiplexing will prove broadly useful for improving the quality of mass spectrometry data in complex backgrounds.

## Electronic supplementary material

ESM 1 (DOCX 3560 kb)

Supplementary Table 1 (XLS 42 kb)

Supplementary Table 2 (XLS 29 kb)

Supplementary Table 3 (XLSX 11 kb)

Supplementary Table 4 (XLS 29 kb)

Supplementary Table 5 (XLS 34 kb)

Supplementary Table 6 (XLS 34 kb)

Supplementary Table 7 (XLS 32.5 kb)

ESM 2 (DOCX 118 kb)

ESM 3 (DOCX 166 kb)

ESM 4 (PDF 1339 kb)

ESM 5 (PDF 1349 kb)

ESM 6 (PDF 143 kb)
