# Supplementary material for: Improving Precursor Selectivity in Data-Independent Acquisition Using Overlapping Windows
Source: J Am Soc Mass Spectrom. 2019 Jan 22;30(4):669–84. doi: 10.1007/s13361-018-2122-8 (PMC6445824; doi:10.1007/s13361-018-2122-8)
Supplement: Supplementary file 1 — (DOCX 3560 kb) [file 13361_2018_2122_MOESM1_ESM.docx]

Supplement: Improving Precursor Selectivity in Data Independent Acquisition Using Overlapping Windows

Dario Amodei*^1^, Jarrett Egertson*^2^, Brendan MacLean^2^, Richard Johnson^2^, Gennifer E Merrihew^2^, Olga Vitek^3^, Parag Mallick^&1^, Michael MacCoss^&2^

1. Department of Radiology, Stanford University, 3155 Porter Drive, Palo Alto, CA

2. Department of Genome Sciences, University of Washington, 3720 15^th^ Ave. NE, Seattle, WA

3. College of Computer and Information Science, Northeastern University, 440 Huntington Ave, Boston, MA

* These authors contributed equally to the work

& Corresponding Authors

Email Addresses:

DA: [damodei@gmail.com](mailto:damodei@gmail.com)

**JE:** [**jegertso@uw.edu**](mailto:jegertso@uw.edu)

**BM:** [brendanx@](mailto:brendanx@)**uw.edu**

**RJ:** [**rj8@uw.edu**](mailto:rj8@uw.edu)

**GM: genn@uw.edu**

**OV:** [o.vitek@northeastern.edu](mailto:o.vitek@northeastern.edu)

**PM:** [**paragm@stanford.edu**](mailto:paragm@stanford.edu)

**MM:** [**maccoss@uw.edu**](mailto:maccoss@uw.edu)

# Supplementary Methods

## Skyline Implementation

The demultiplexing algorithm, auxiliary tools for peak picking and chromatogram analysis, and feature scores from the mProphet algorithm are implemented in the open-source targeted proteomics software Skyline [^1^](#_ENREF_1), which is available at <http://skyline.maccosslab.org>. Skyline is part of the ProteoWizard project [^2^](#_ENREF_2), which can be checked out as an SVN repository at https://svn.code.sf.net/p/proteowizard/code/trunk/pwiz/.

Overlap-based demultiplexing is available under Settings -> Transition Settings -> Full Scan -> Isolation Window Scheme -> Deconvolution. This feature is capable of analyzing and demultiplexing arbitrary combinations of overlapped windows having any isolation width and relative offset and is not limited to the 20 *m/z* windows, half-overlapped windows, or alternating window cycles employed in the present manuscript. For instance, 25 *m/z* windows collected with an offset of 8 *m/z* from each consecutive window to the next, can be demultiplexed by Skyline as easily as the current acquisition scheme can.

For comparing peptide identifications, we used several feature scores and a decoy null model from the mProphet algorithm [^3^](#_ENREF_3) in Skyline which assigns confidence scores to peak identifications of peptides and also attempts to help pick the correct peak from a chromatogram. The use of this algorithm is described in the tutorial here: https://skyline.ms/_webdav/home/software/Skyline/%40files/tutorials/PeakPicking_2-5.pdf

# Supplementary Results and Discussion

## Bovine Spike-in Experiment

### Extended m/z Range Comparison of 20 m/z Isolation Methods for Identification and Quantification

As a further check of robustness and generality, we also extended the analysis of peptide identification and quantification in the bovine spike-in mix (see **Results**) beyond the 32 DDA-identified peptides in the 500-700 *m/z* range to the full 500-900 *m/z* range, which contained 42 DDA-identified peptides. This extension was only possible for the 20 *m/z* and 20 *m/z*/overlap (with and without demultiplexing) schemes, because the 700-900 *m/z* range is not covered in the 10 *m/z* scheme. The results were qualitatively similar to those observed for the 500-700 *m/z*.

### Individual Fragment Ion Lower Limit of Quantification

We measured the lower limit of quantification of the 10 *m/z*, 20 *m/z* and 20 *m/z* overlap workflows for individual fragment ions in addition to at the peptide level. This gives a lower-level view into the relative sensitivity of the analytical workflows, and also compares acquisition workflows in a way that does not depend on fragment ion selection or algorithmic choices in combining fragment ion measurements into a peptide measurement. We found roughly the same patterns at the fragment ion level as at the peptide level. The 10 *m/z* method generally had the largest number of fragment ions linear down to a given concentration, followed by the 20 *m/z* overlap/demultiplexing, and the standard 20 *m/z* method. Similarly, to the peptide-level results, this relative ordering is what would be expected given the precursor selectivity of the acquisition methods.

## Rapamycin Experiment

### Discrepancies in the Abundance Change of β7 between the DDA and DIA Results

The β7 subunit is the only case where the DIA (20 *m/z* overlap) and DDA techniques show statistically significant changes in opposite directions. The DDA method captures data on 4 peptides for this protein, and the DIA method captures three. DDA has more peptides because it covers a wider *m/z* range (400-2000 *m/z*) than the DIA technique (500-900 *m/z*). Looking at the signal from each peptide separately (Supplementary Figure 9A-B), it is clear that the discrepancy arises from the peptide NFSLAIIDK++ which is measured by both techniques. The DDA data shows the peptide decreasing in abundance, while DIA shows it increasing in abundance. The data from both techniques appear to be influenced by chemical noise for this peptide, with the DDA data being influenced to a greater extent (Supplementary Figure 9C, top panel). Additionally, the DDA data appears to have quite a bit of noise. This is likely due to the wide *m/z* range analyzed in each DDA scan. When a wider *m/z* range is analyzed it is more difficult to tune the ion optics of the instrument to isolate such a wide *m/z* range and the dynamic range in abundance of the isolated ion populations is greater. The middle panel of Supplementary Figure 9C shows the extracted MS signal from the DIA data which is a scan only analyzing the range from 500-900 *m/z*. In this case, the signal is much less noisy, yet chemical noise is still very apparent and the resulting inference from MSStats is still that β7 is downregulated (see “MS Data vs MS/MS Data for this Analysis” in this Supplement). Therefore, the discrepancy between DIA and DDA on the direction of the abundance change is most likely caused by chemical noise in the MS signal rather than the noise cause by the wide isolation of the MS scans in the DDA data.

### The Impact of Analyzing a Wider m/z Range with DDA than DIA

The DDA analysis acquires an MS scan on a 1600 *m/*z range (400-2,000 *m/z*) followed by MS/MS analysis of a subset of the peptides detected in the MS scan. The 20 *m/z* overlap workflow analyzes the range from 490-900 *m/z*, with the range from 500-890 *m/z* being the optimal range for analysis after demultiplexing (see Discussion in main text). The DDA analysis covers a roughly 4x wider *m/z* range than DIA and should therefore be able to include more peptides per protein in its analysis. Despite the wider *m/z* range analyzed, DDA averages 2.2 peptides per protein, while DIA averages 1.8 (Supplementary Figure 1). The additional *m/*z analyzed by DDA allowed it to detect a change in RPT5 which was not detectable by DIA.

We further analyzed the impact of covering a wider *m/z* by analyzing the DDA data using only peptides between 500 and 900 *m/z* (the same range covered by DIA) in **Supplementary Figure 7**: DDA Results Suggest that DIA Could Benefit from Analyzing a Wider m/z Range. The reduction in the number of peptides used in the analysis compromises the sensitivity of the DDA method. There are many examples of proteins where a significant change in abundance was detected using standard DDA, but after reducing the *m/z* range the change is no longer detected (ex. RPN 3, 5, 7, etc.).

Based on these observations, the DIA analysis workflow we have developed may be even more sensitive if the *m/z* range covered is expanded. However, to expand the *m/z* range covered, there would have to be tradeoff in precursor selectivity (isolation window width), duty cycle (number of scans), resolving power and/or scan fill time. Future instrumentation may be able to cover a wider *m/z* range without any of these tradeoffs.

### MS Data vs. MS/MS Data

To make a direct comparison between using MS and MS/MS data for quantification, we analyzed the MS data from our 20 *m/z* overlap DIA runs and compared it to the MS/MS data from the same runs. The MS scans were interlaced with the MS/MS scans in each DIA run with 1 MS scan every 10 MS/MS scans. The MS scans in the DIA runs only analyze the range from 500-900 *m/z* and should be more sensitive for quantification compared to using the DDA MS scans which analyze 400-2000 *m/*z. Using the MS data, the signal for the decreased abundance of the RPT proteins and increased abundance of the catalytic core proteins is not as strong as with the MS/MS data (Supplementary Figure 8). While the MS data on its own does not appear to be as sensitive as the MS/MS data from the DIA runs, future workflows may combine the MS and MS/MS data for comparative quantitation and generate even better results.

# Supplementary Figures

## Supplementary Figure 1: DDA Detects More Peptides Per Protein than DIA


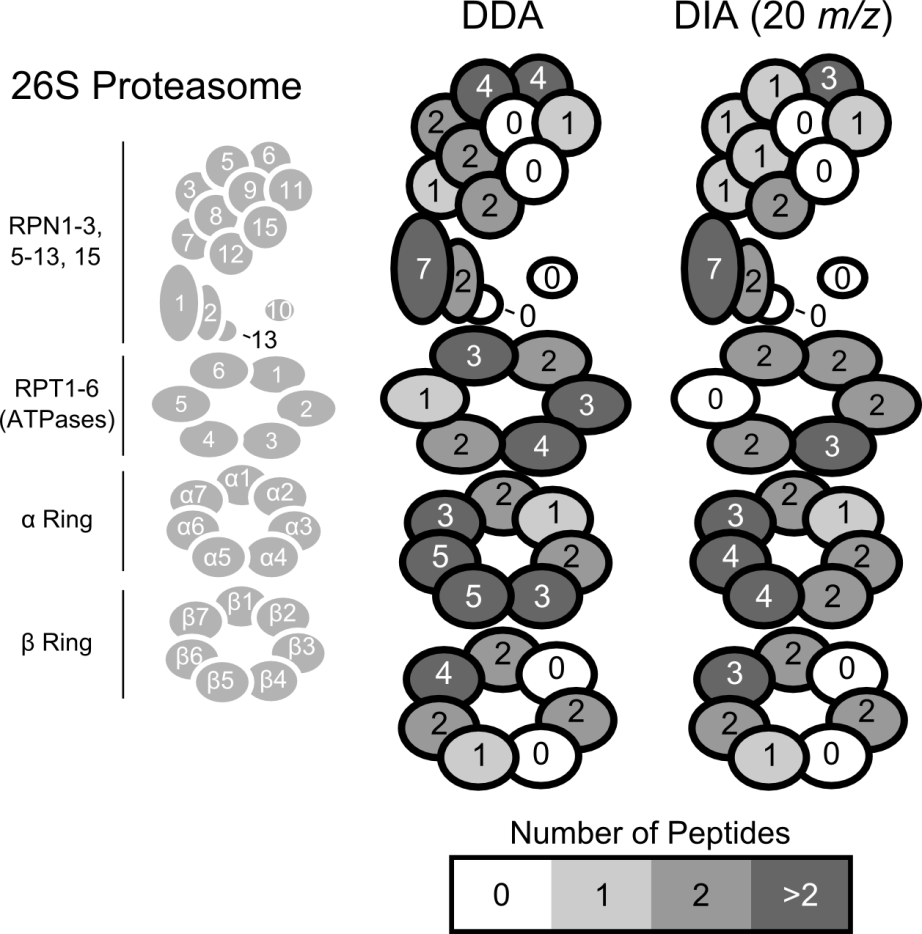


The number of peptides used for quantification of each protein in the proteasome by the DDA and DIA (20 *m/z* overlap) techniques are illustrated here. The identities of each protein are indicated in the legend on the left.

## Supplementary Figure 2: The Quantified Improvement in Precursor Selectivity is Robust to Changes in Spike-in Level


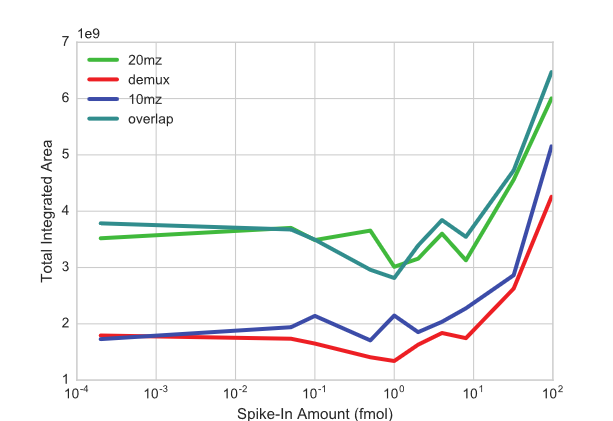


Chromatograms were extracted and integrated for all transitions of the 37 peptides of interest in the bovine spike-in dataset across multiple spike-in amounts. The integrated area for each transition, averaged over all peptides and replicates is plotted for each amount of spike-in protein and condition. At all spike-in points, the total integrated area for the 20 *m/z* demultiplexed DIA method is at the level of the 10 *m/z* DIA data despite analyzing twice the precursor *m/z* range. In this figure “overlap” is the 20 *m/z* overlapping windows acquisition without demultiplexing applied, and “demux” is with demultiplexing applied.

## Supplementary Figure 3: Overlapping Window DIA Improves Sensitivity on the Fragment Ion Level


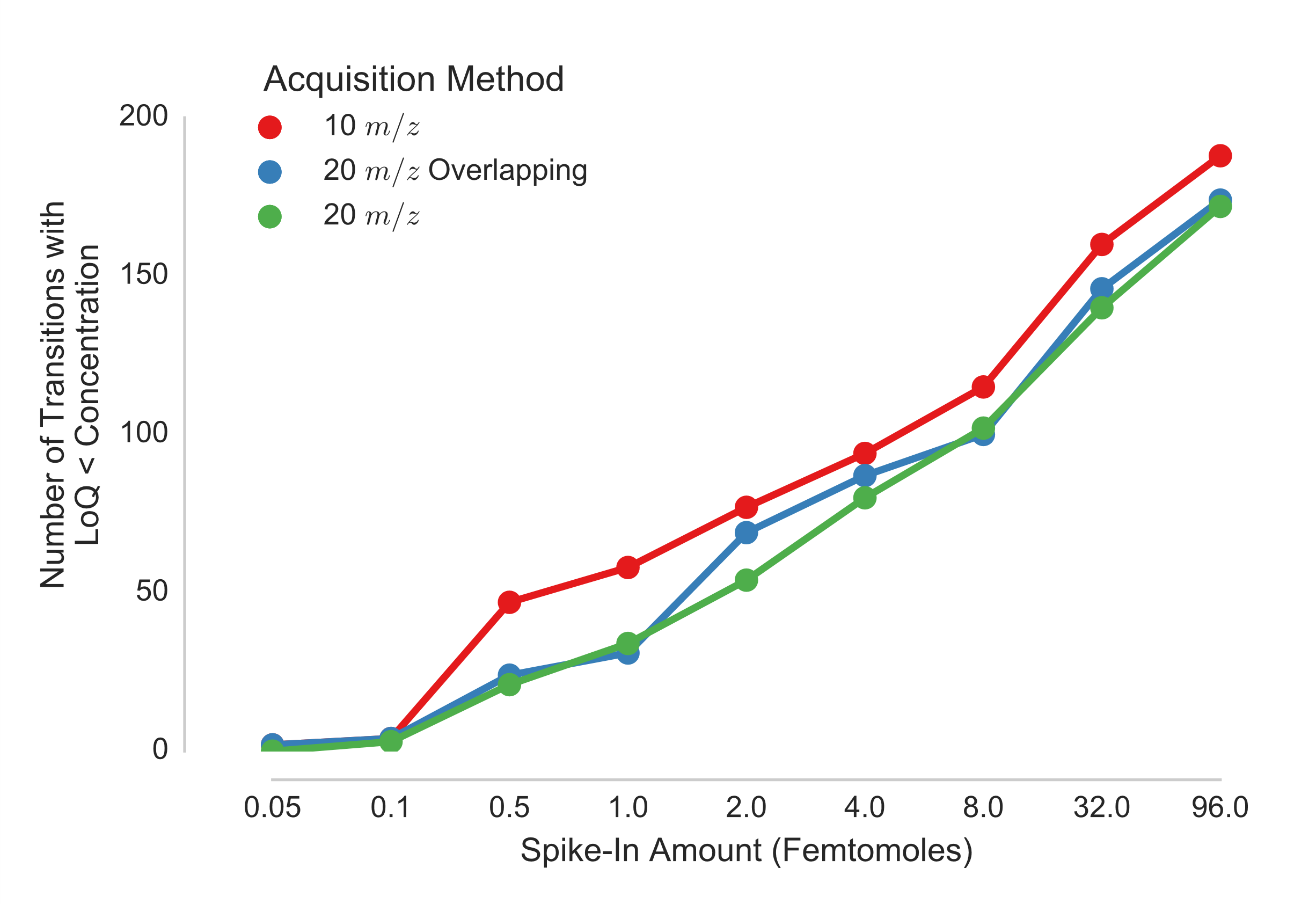


The lower limit of quantitation for each individual fragment ion measured in the bovine spike-in experiment was computed (see **Supplementary Methods**) and the number of fragment ions below various sensitivity thresholds is plotted for each acquisition method.

## Supplementary Figure 4: Overlapping Windows Improves Quantification in the Full 500-900 m/z Precursor Range Analyzed


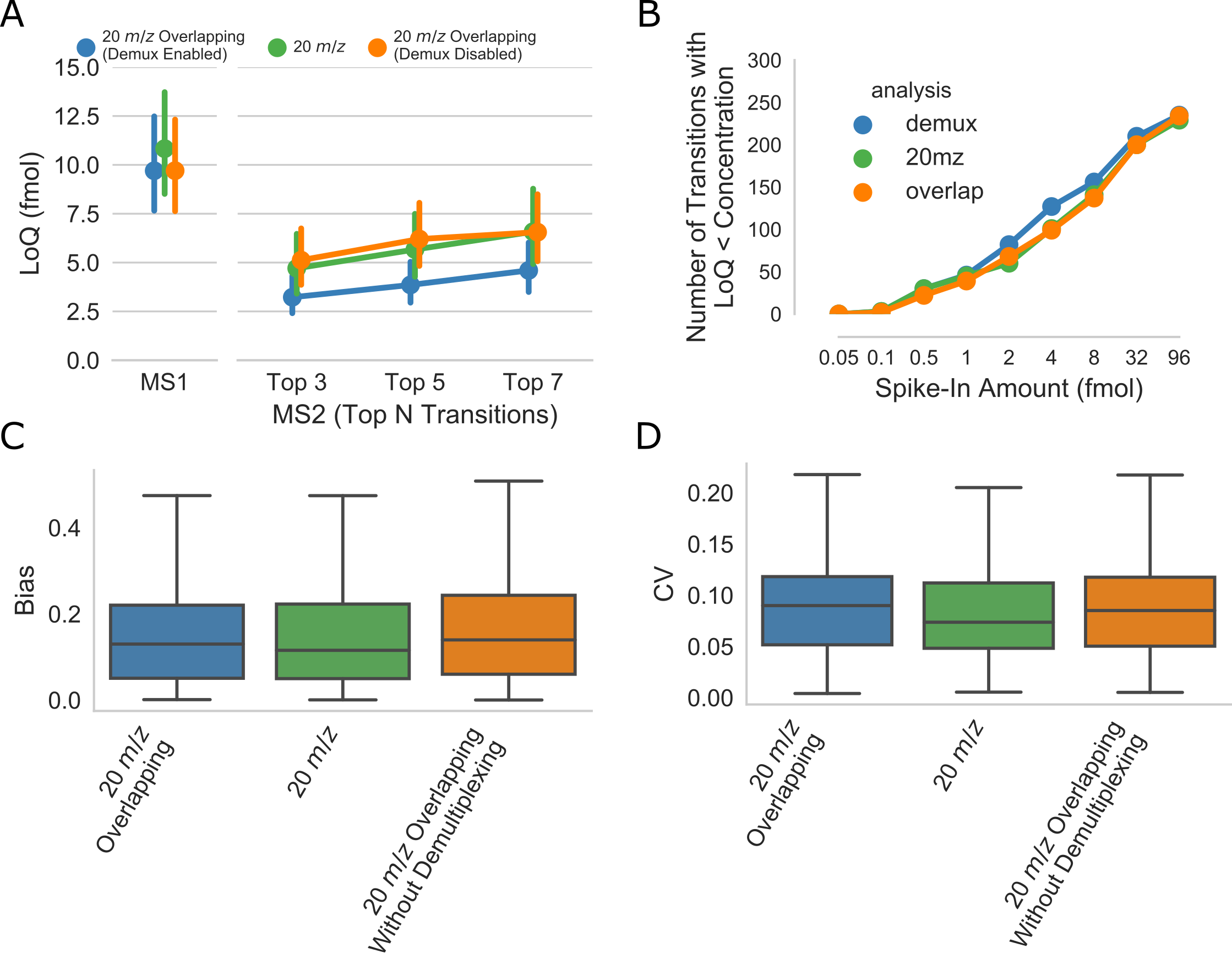


Because the 10 *m/z* window acquisition method does not (by design) cover peptides in the 700-900 *m/z* range, all comparisons display only the 20 *m/z* windows, 20 *m/z* windows with overlap, and 20 *m/z* windows with overlap and demultiplexing workflows. The panels display the same comparisons as those in the main text, but for all 42 precursors in the 500-900 *m/z* range rather than just the 32 precursors in the 500-700 *m/z* range. **(A**: The average lower limit of quantitation for the 42 precursors summing over the top-N transitions (based on DDA library spectra). **(B):** The lower limit of quantification on the transition-level. The number of transitions below various sensitivity thresholds is plotted for each acquisition method. **(C):** The accuracy for each method is plotted based on the difference in the observed concentration and the measured concentration averaged over each spiked-in analyte. Error bars are the standard error of the mean. The technique for determining the observed concentration can be found in the **Supplementary Methods** section. **(D):** The reproducibility for each method is plotted as the coefficient of variation (CV) in the intensity of each technique across replicates.

## Supplementary Figure 5: mProphet Score Distributions for Target and Decoy Peptides

A: (10 *m/z*)


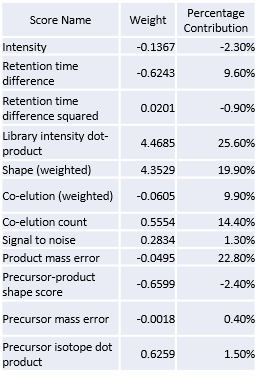


B: (20 *m/z*)


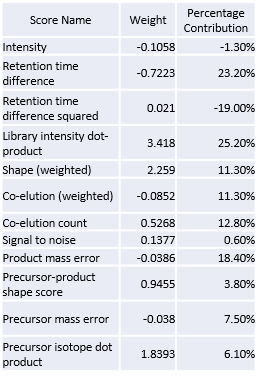


C: (20 *m/z* + overlap)


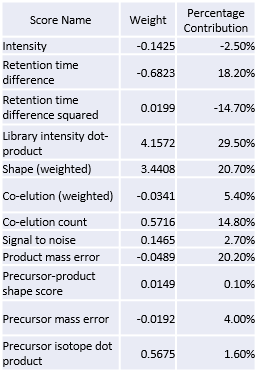


Plot of the mProphet scoring models used to score candidate peaks and determine peptide identifications. Target peaks (blue) and decoys (orange) from all concentrations are shown. Note that most very low abundance target peptides are not identified. The weight and percent contribution of each individual feature comprising the composite score are shown in the tables to the right of each composite score plot. (a): model for 10 *m/z* acquisition, (b): model for 20 *m/z* acquisition, (c): model for 20 *m/z* overlapping acquisition with computational demultiplexing.

## Supplementary Figure 6: mProphet with no Retention Time


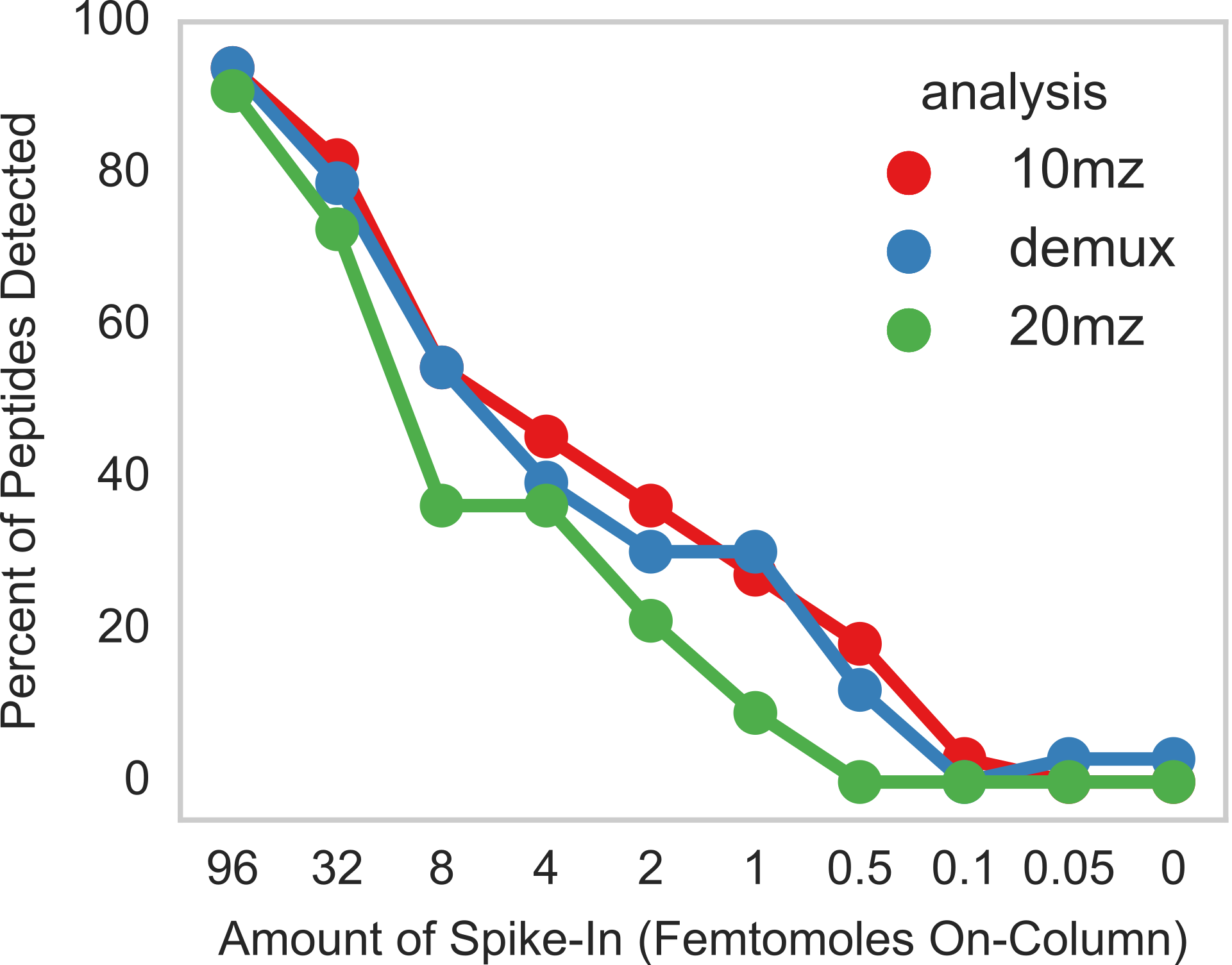


A comparison of mProphet detection as in main text Figure 2G except without using retention time features or information. Peak groups are not filtered by retention time for this analysis, and retention time-based scoring features are not included in the mProphet analysis (see “Peptide Detection” in main text methods). When no retention time information is available, the overlapping approach (“demux”) outperforms the 20 *m/z* approach by a greater margin than when retention time information is available. This is potentially due to the difference in precursor selectivity between the overlap and 20 *m/z* approach being partially compensated for by improved retention time selectivity.

## Supplementary Figure 7: DDA Results Suggest that DIA Could Benefit from Analyzing a Wider m/z Range


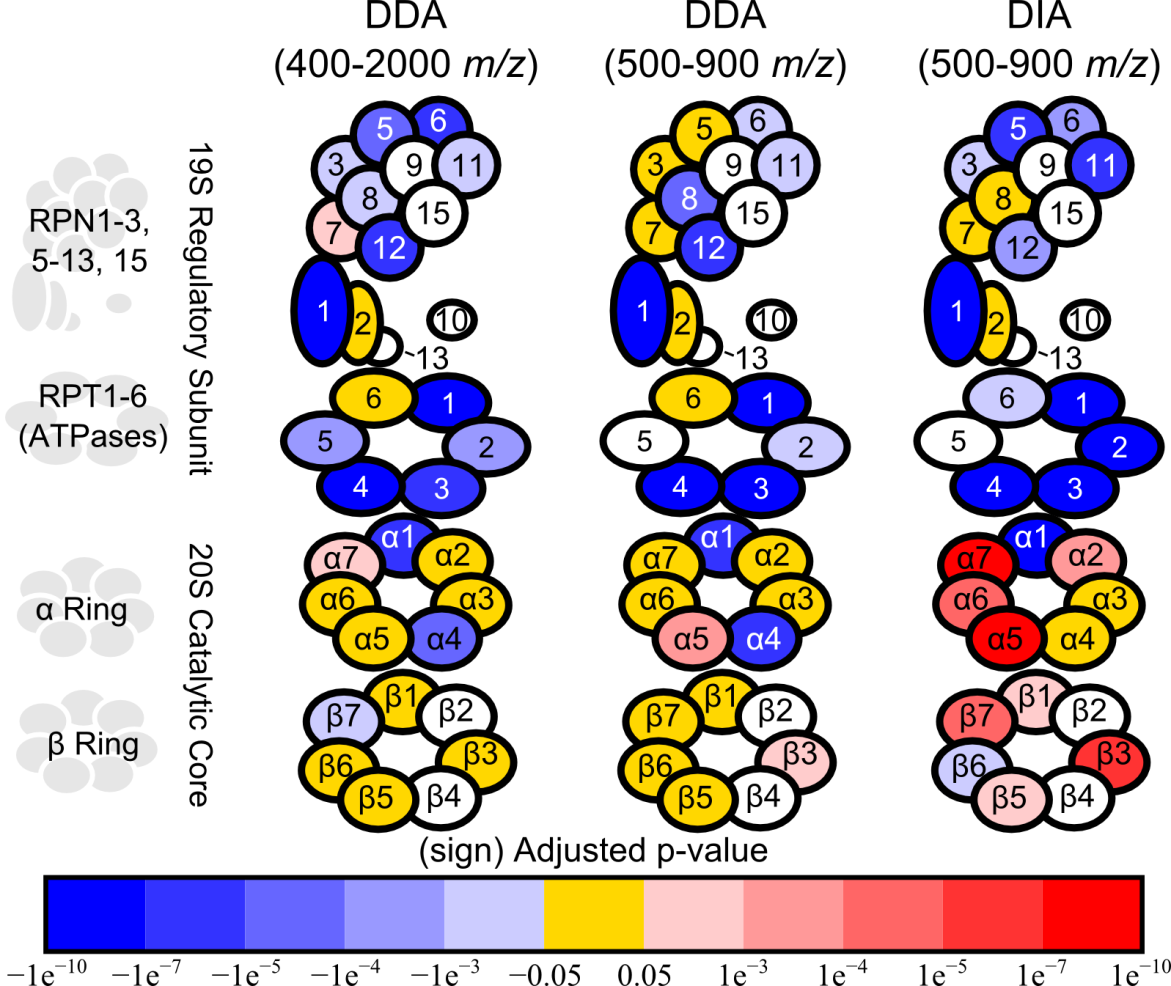


The proteins of the 26S proteasome with significantly increased or decreased abundance in response to growth in rapamycin (according to p-values calculated by MSstats) detected using all peptides in the DDA MS1 data (left), only peptides with precursor *m/z* 500-900 from the same DDA data (center), and peptides with precursor *m/z* 500-900 from 20 *m/z* overlap DIA data are plotted. Proteins in white had no peptides detected, gold had at least one peptide detected but no significant change in abundance, red had increased abundance, and blue had decreased abundance. Darker reds and blues indicate a more significant p-value. The DDA 400-2000 *m/z* (left) and DIA results (right) are identical to that in the main text.

## Supplementary Figure 8: MS/MS Data are More Sensitive than MS Data for Detection of Protein Changes in Response to Growth in Rapamycin


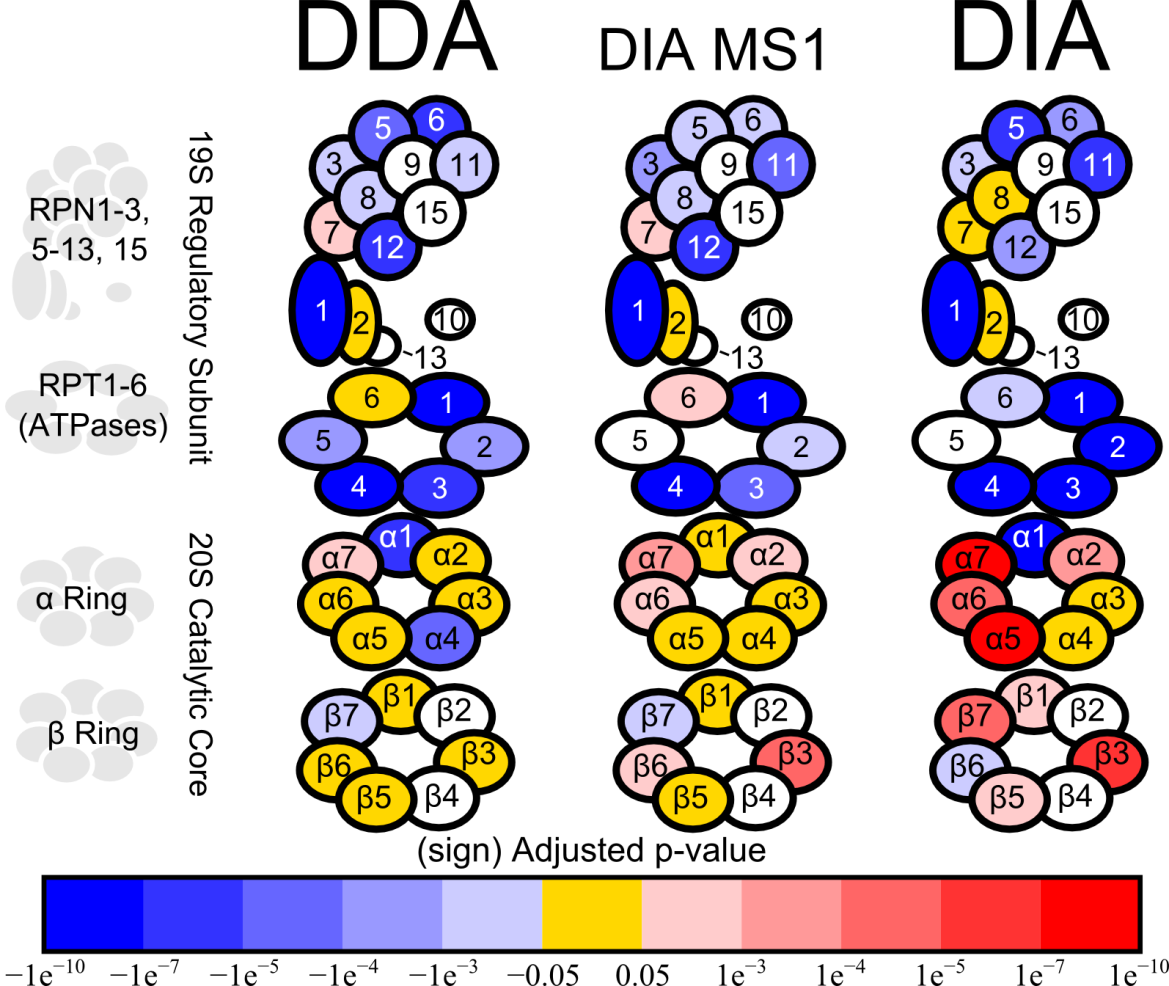


The proteins of the 26S proteasome with significantly increased or decreased abundance in response to growth in rapamycin (according to p-values calculated by MSstats) detected using peptides in the DDA MS1 data (left), the 20 m/z overlapped DIA MS1 data (center), and the 20 m/z overlapped DIA MS/MS data (right) are plotted. The DDA MS1 data covers a wider m/z range (400-2000 m/z) than the DIA MS1 data and MS/MS data (500-900 m/z). The MS1 scans in the DIA data were acquired once every 10 MS/MS scans. Proteins in white had no peptides detected, gold had at least one peptide detected but no significant change in abundance, red had increased abundance, and blue had decreased abundance. Darker reds and blues indicate a more significant p-value. The DDA 400-2000 m/z (left) and DIA results (right) are identical to that in the main text.

## Supplementary Figure 9: Chemical Noise Causes a Discrepancy in the Change in β7 Abundance in Response to Rapamycin Detected by DDA and DIA


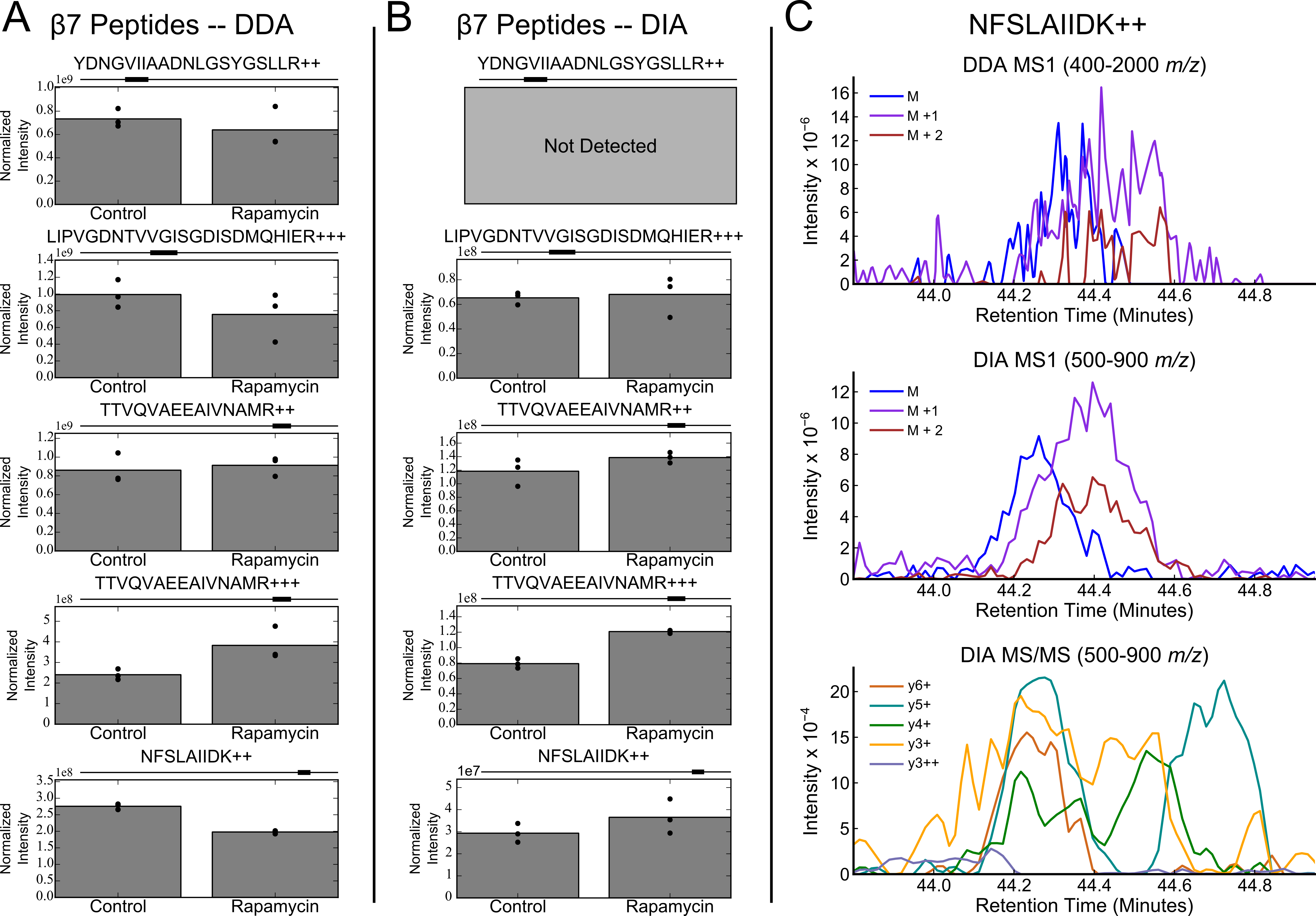


The signal (intensity normalized by MS1 TIC) for the 5 peptides of the β7 protein measured by DDA and DIA are plotted in panel A and B, respectively. For each peptide, the averaged signal from the control (wild-type) and rapamycin experiment are plotted as bars, with the signal from each individual replicate overlaid as dots. Just below the peptide label in each plot is a diagram indicating the location of the peptide (thick bar) on the protein (thin bar). Panel C shows the extracted chromatograms from one of the control samples. The top panel is the precursor ion chromatograms extracted from DDA MS1 data where each MS scan analyzes from 400-2000 *m/z*, the middle panel is precursor chromatograms from DIA MS data (500-900 *m/z*), and the bottom panel shows product ion chromatograms from the DIA data.

# Supplementary Tables

## Supplementary Table 1: Run Order for Bovine Spike-in Experiment

(SupplementaryTable1.xlsx, too large to embed here)

The run order and file names for all data acquired in the bovine spike-in experiment are listed in this table.

## Supplementary Table 2: Run Order for Rapamycin Experiment

(SupplementaryTable2.xlsx, too large to embed here)

The run order and file names for all data acquired in the rapamycin experiment are listed in this table.

## Supplementary Table 3: Peptides used in the Bovine Dilution Data Analysis

(SupplementaryTable3.xlsx, too large to embed here)

This table lists the tryptic peptides measured in the bovine spike in analysis. A subset of peptides were excluded from the analysis because they were not measured in a single LC-MS/MS run due to an acquisition error.

## Supplementary Table 4: Proteins Targeted for the Analysis of Proteasome-mediated Degradation in Yeast Grown in Rapamycin

(SupplementaryTable4.xls, too large to embed here)

This table lists the proteins related to proteasome mediated protein degradation that were chosen as targets for a comparative analysis of abundance between yeast grown with and without rapamycin. The number of DDA-identified peptides and precursors for each protein is shown. The number of these peptides/precursors (a subset) that were extracted from the DIA data is also listed. Proteins with zero peptides identified were not included in the MSstats analysis.

## Supplementary Table 5: MSstats Output from the Rapamycin Analysis using DDA

(SupplementaryTable5.xls, too large to embed here)

32 proteins with peptides identified by DDA relevant to proteasome-mediated protein degradation were processed by MSstats to determine which had significant changes in abundance in response to growth in rapamycin. This table contains the output from the MSstats analysis on the DDA data.

## Supplementary Table 6: MSstats Output from the Rapamycin Analysis using DIA

(SupplementaryTable6.xls, too large to embed here)

29 proteins with peptides identified by DDA were processed by MSstats to determine which proteins had significant changes in abundance in response to growth in rapamycin. This table contains the output from the MSstats analysis on the DIA data which analyzed 29 of the 32 proteins with peptide identified by DDA (some were only identified by peptides outside of the 500-900 *m/z* range covered by the DIA method).

# Supplementary Data

The archive supplementary_data.tar.gz includes dilution curves for the 32 spike-in peptides analyzed by standard 10 *m/z,* standard 20 *m/z*, and 20 *m/z* with overlap (with and without demultiplexing indicated as “demux” and “overlap”). Each plot shows the individual measurements (indicated as dots), as well as a bilinear curve fit calculated by fitting a curve with function:

$$f\left( x \right)=\left\{ \begin{aligned} s\left( x-c \right)+n, &for x>c \\ n, &for x<c \end{aligned} \right.$$

Where f(x) is the intensity of the fit curve, and x is concentration. *n, c,* and *s* are free parameters of the fit roughly corresponding to the intensity of a blank measurement, the “inflection point” between measurements with no signal and measurements with signal, and the slope of the intensity <-> concentration fit in the linear portion of the quantitative range. The LoQ is indicated as a circle at the concentration point of the LoQ calculated as described in the main text (see Methods – Peptide Quantification and Limit of Quantification Analysis).

# References

1 Maclean, B. *et al.* Skyline: an open source document editor for creating and analyzing targeted proteomics experiments. *Bioinformatics* **26**, 966-968, doi:10.1093/bioinformatics/btq054 (2010).

2 Chambers, M. C. *et al.* A cross-platform toolkit for mass spectrometry and proteomics. *Nature Biotechnology* **30**, 918-920, doi:10.1038/nbt.2377 (2012).

3 Reiter, L. *et al.* mProphet: automated data processing and statistical validation for large-scale SRM experiments. *Nature methods* **8**, 430-435, doi:10.1038/nmeth.1584 (2011).
