# Supplementary material for: Improving Precursor Selectivity in Data-Independent Acquisition Using Overlapping Windows
Source: J Am Soc Mass Spectrom. 2019 Jan 22;30(4):669–84. doi: 10.1007/s13361-018-2122-8 (PMC6445824; doi:10.1007/s13361-018-2122-8)
Supplement: Supplementary file 8 — (DOCX 118 kb) [file 13361_2018_2122_MOESM8_ESM.docx]

## Full Spectrum Demultiplexing using MSConvert

This is a quick demonstration on how to use msconvert to generate a demultiplexed dataset from an input dataset containing overlap multiplexed spectra. In the case of the overlapped window approach described in this manuscript, the output from msconvert will contain twice as many spectra as the input (two demultiplexed spectra are generated from each acquired MS/MS spectrum). This tutorial uses msconvert distributed with ProteoWizard version 3.0.11579 with vendor libraries downloadable here: http://proteowizard.sourceforge.net/downloads.shtml

**Open MSConvert and select the “Browse” button to select the input overlap-multiplexed files.**


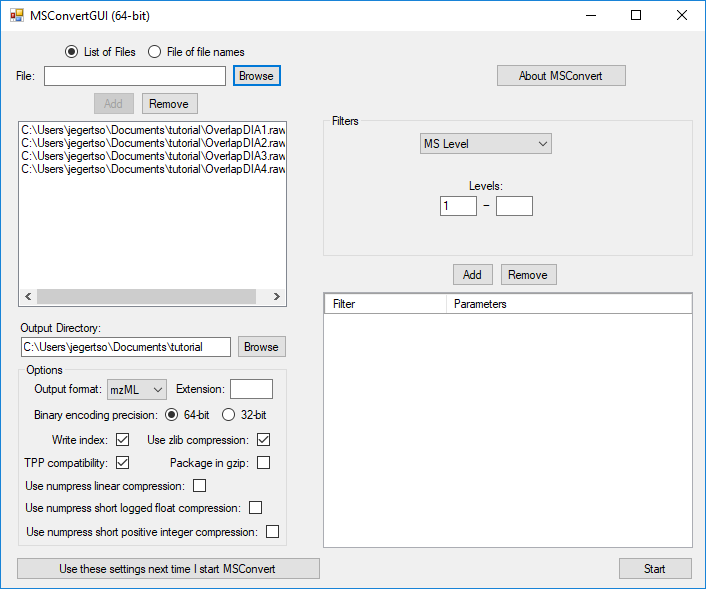


**OPTIONAL: Change the output format for the file using the “Output format” dropdown menu. This tutorial outputs mzML.**

**Add a “peak picking” filter to the data by selecting the options indicated below and clicking the “Add” button:**


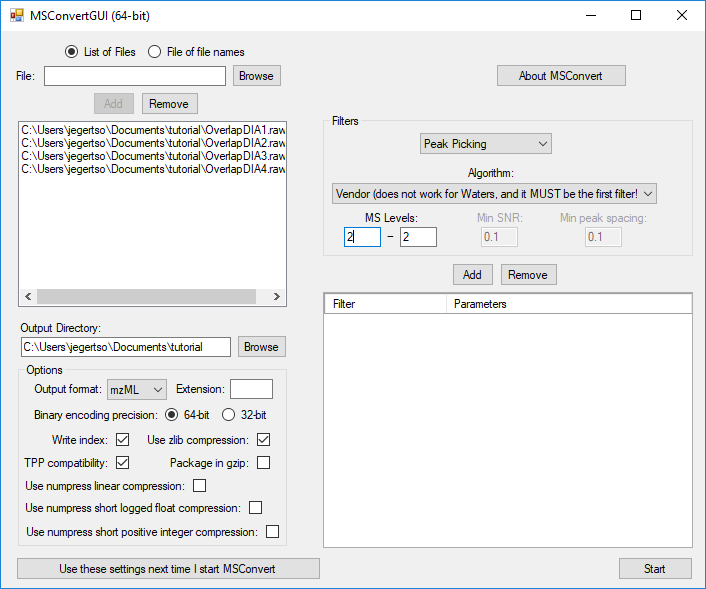


This causes the MS2 data to be centroided prior to demultiplexing, which is currently a requirement for full-spectrum demultiplexing using msconvert (but not for demultiplexing using Skyline). If the data were acquired with centroiding enabled, this step will have no effect and demultiplexing will proceed as expected.

**Add a “demultiplexing” filter with optimization set to “Overlap only” by selecting the options indicated below and clicking the “Add” button:**


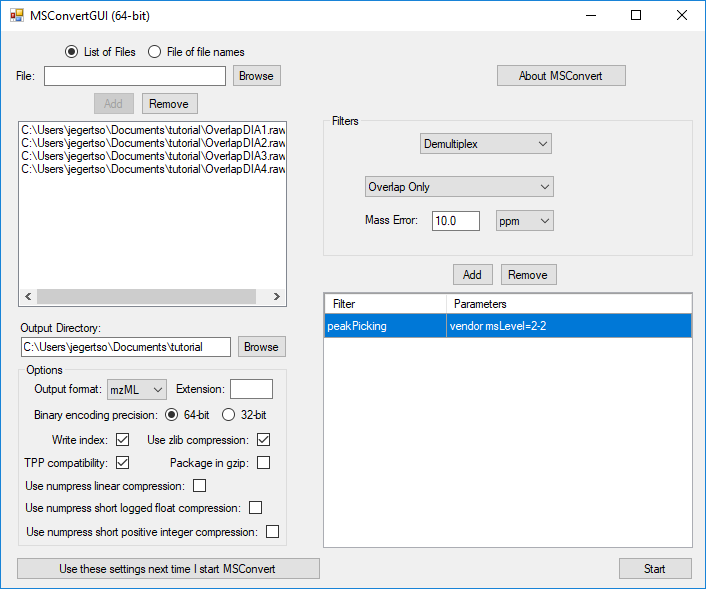


Note that the mass error may need to be adjusted depending on instrument platform. The mass error should be set to the maximum error expected in *m/*z measurement of the same analyte in subsequent spectra. Note that this measurement is of expected deviation of a measurement from spectrum to spectrum, not its deviation from the correct theoretical *m/z* (mass accuracy).

**Click “Start” to output demultiplexed files.**
