# Supplementary material for: Improving Precursor Selectivity in Data-Independent Acquisition Using Overlapping Windows
Source: J Am Soc Mass Spectrom. 2019 Jan 22;30(4):669–84. doi: 10.1007/s13361-018-2122-8 (PMC6445824; doi:10.1007/s13361-018-2122-8)
Supplement: Supplementary file 9 — (DOCX 166 kb) [file 13361_2018_2122_MOESM9_ESM.docx]

## Generating an Overlapping Window Isolation List using Skyline

This is a quick demonstration on how to use Skyline to generate an overlapping-window isolation window list suitable for acquisition using the approach described in this manuscript and downstream computational demultiplexing. This tutorial uses Skyline v 3.7 (<http://skyline.ms>).

**Start with a blank Skyline Document:**


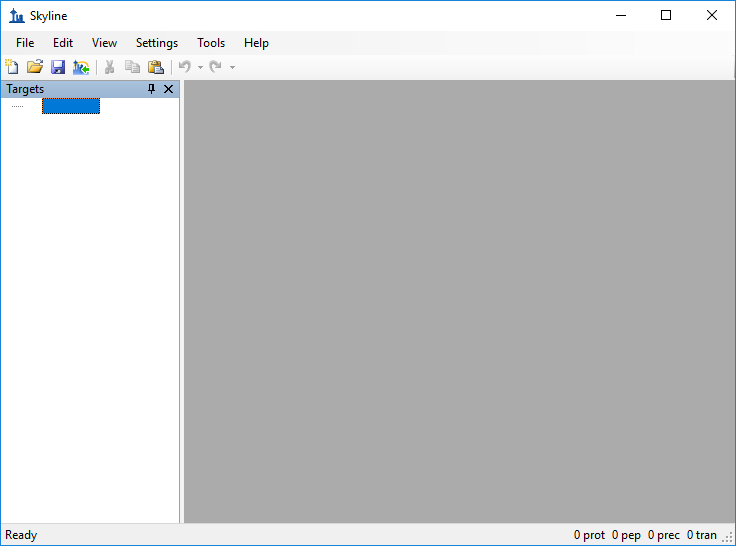


**Select Settings -> Transition Settings… and click on the “Full Scan” tab**


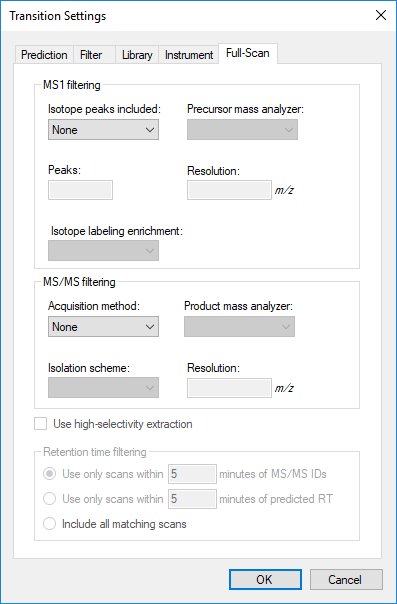


**Under MS/MS filtering Acquisition method select “DIA” and then click the drop-down menu under “Isolation scheme” and selecte “Add…”**


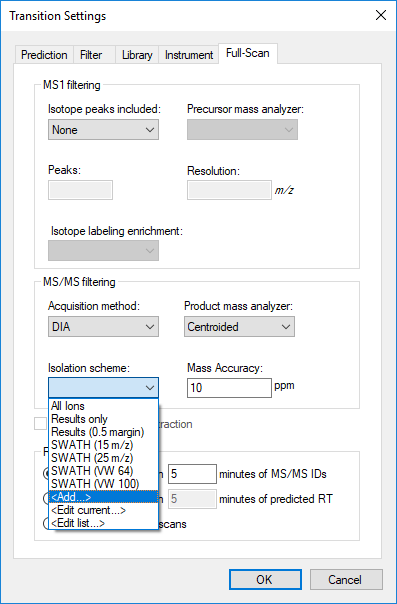


**Enter a name for the “isolation scheme” and select the “Prespecified Isolation Windows” radio button.**


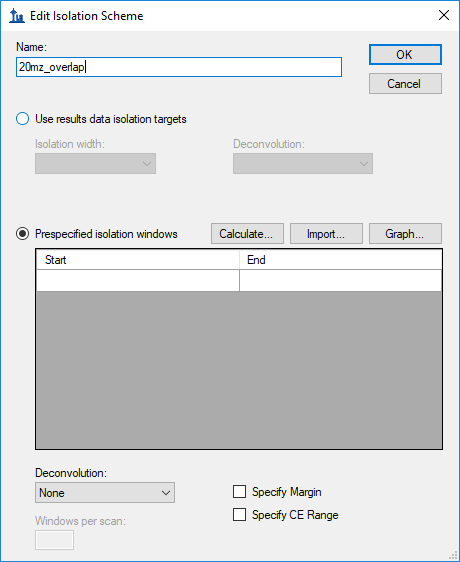


**Click on the “Calculate…” button and enter the parameters for the method you would like to generate (here, a 20 *m/z* overlapping window method covering 500 – 900 *m/z* is generated).**


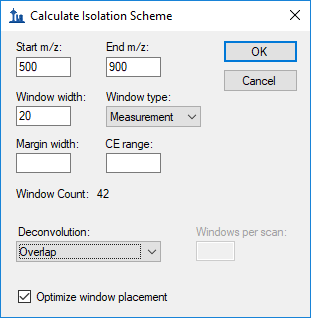


**Select OK to close the “Calculate Isolation Scheme Window” and selected “OK” again to close the “Edit Isolation Scheme” window. In the Transition Settings Window, set the Product Mass Analyzer to “Orbitrap”.**


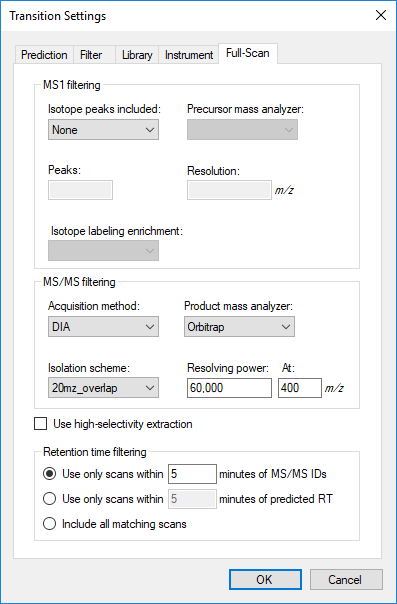


**Click the OK button to close the Transition Settings window and in the main Skyline window select File -> Export -> Isolation List**


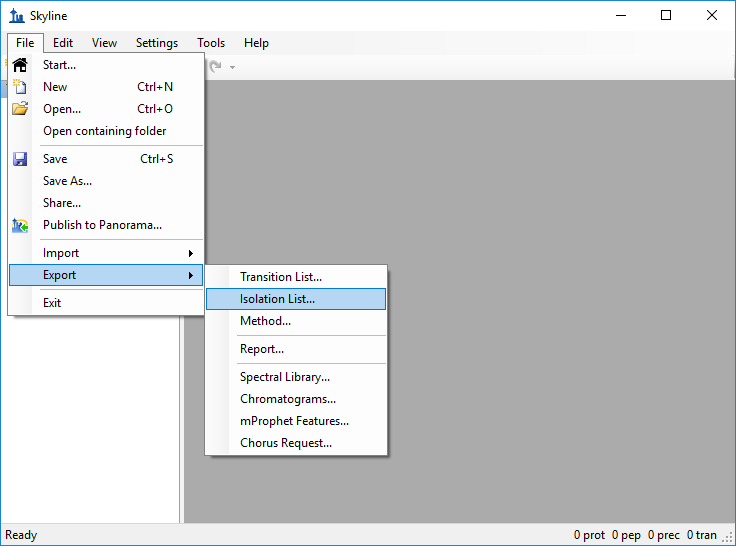


**The below window will appear. Select OK and choose a file location to save the list of isolation windows to.**


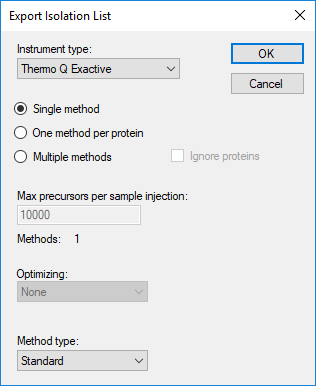


The resulting isolation list contains the isolation centers of each isolation window in the order that they should be acquired. Note that the isolation centers will need to be regenerated using this approach if any of the other acquisition parameters such as isolation width or *m/z* range covered are changed.
