# Supplementary material for: Improving Precursor Selectivity in Data-Independent Acquisition Using Overlapping Windows
Source: J Am Soc Mass Spectrom. 2019 Jan 22;30(4):669–84. doi: 10.1007/s13361-018-2122-8 (PMC6445824; doi:10.1007/s13361-018-2122-8)
Supplement: Supplementary file 10 — (PDF 1339 kb) [file 13361_2018_2122_MOESM10_ESM.pdf]

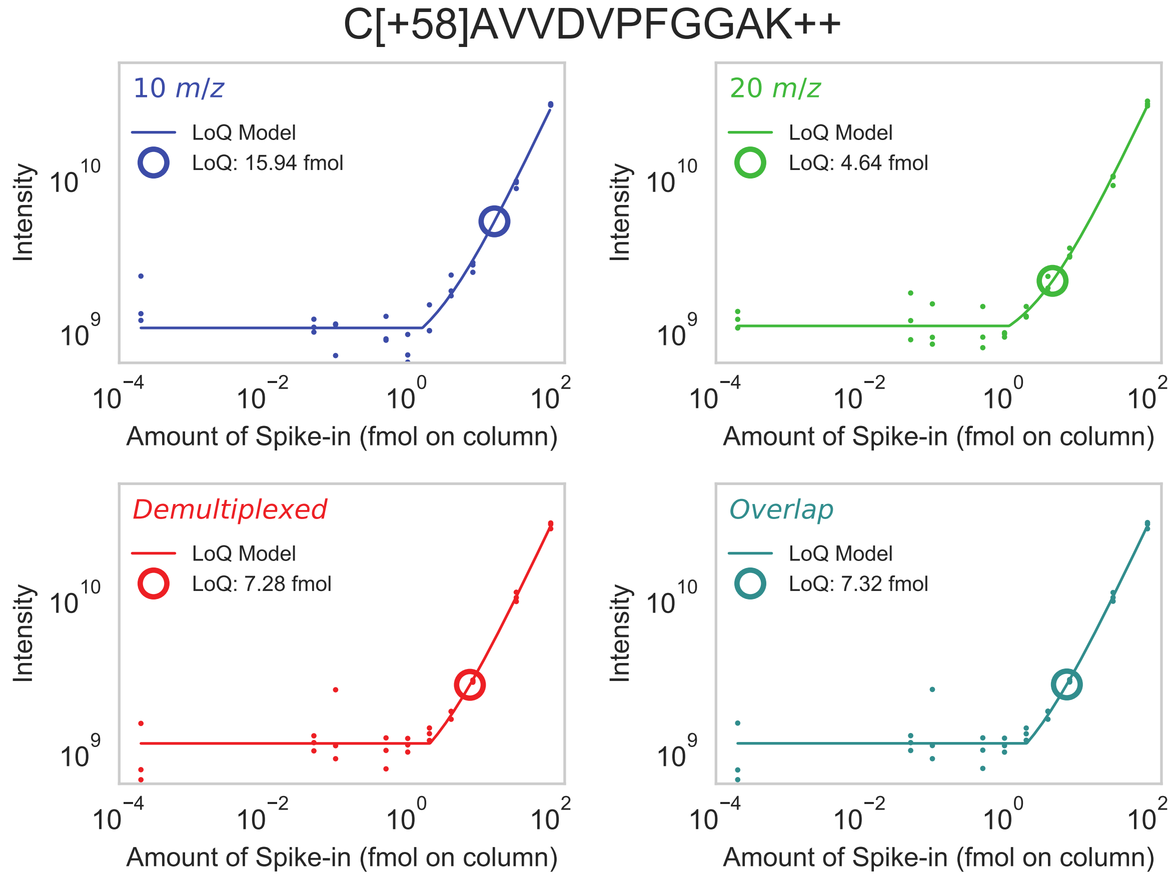
C[+58]AVVDVPFGGAK++
10 m/z
LoQ Model
LoQ: 15.94 fmol
20 m/z
LoQ Model
LoQ: 4.64 fmol
Demultiplexed
LoQ Model
LoQ: 7.28 fmol
Overlap
LoQ Model
LoQ: 7.32 fmol
Intensity
Amount of Spike-in (fmol on column)

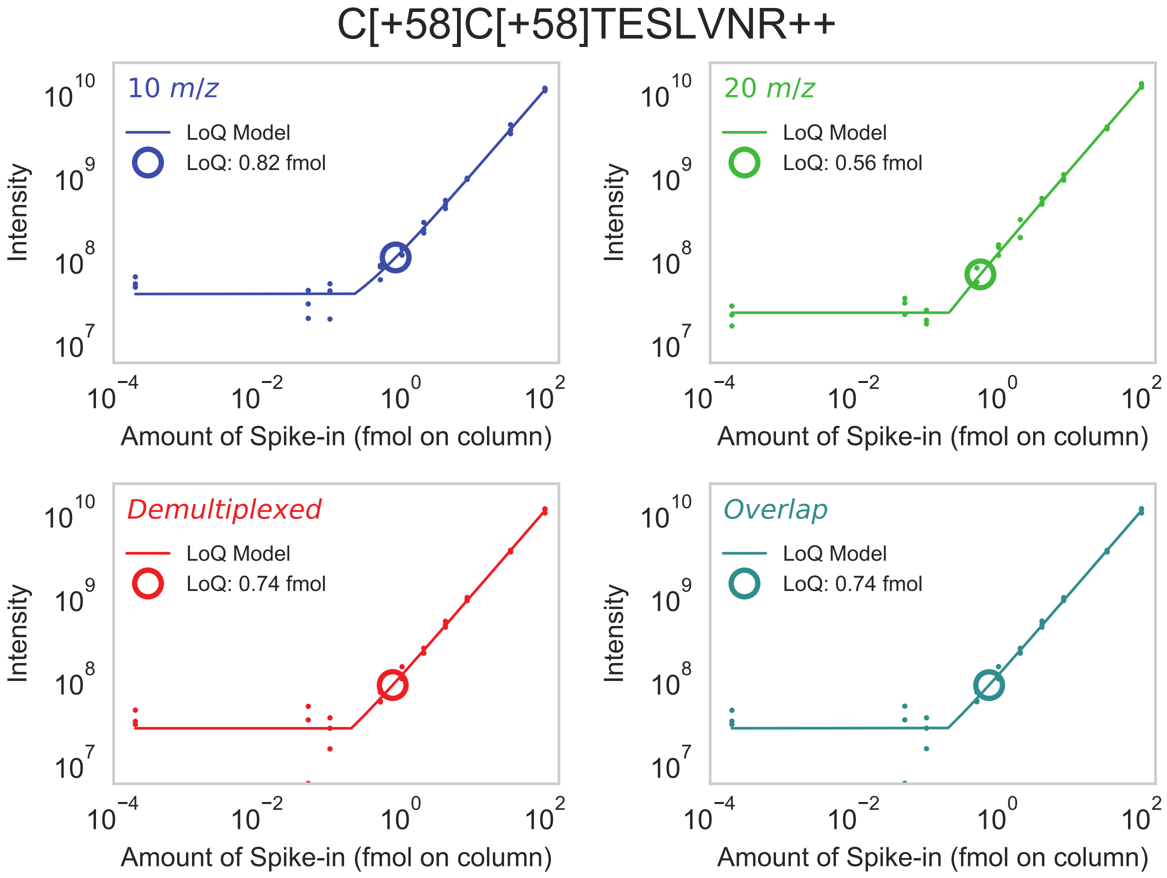

C[+58]C[+58]TESLVNR++
10 m/z
LoQ Model
LoQ: 0.82 fmol
20 m/z
LoQ Model
LoQ: 0.56 fmol
Demultiplexed
LoQ Model
LoQ: 0.74 fmol
Overlap
LoQ Model
LoQ: 0.74 fmol
Intensity
Amount of Spike-in (fmol on column)

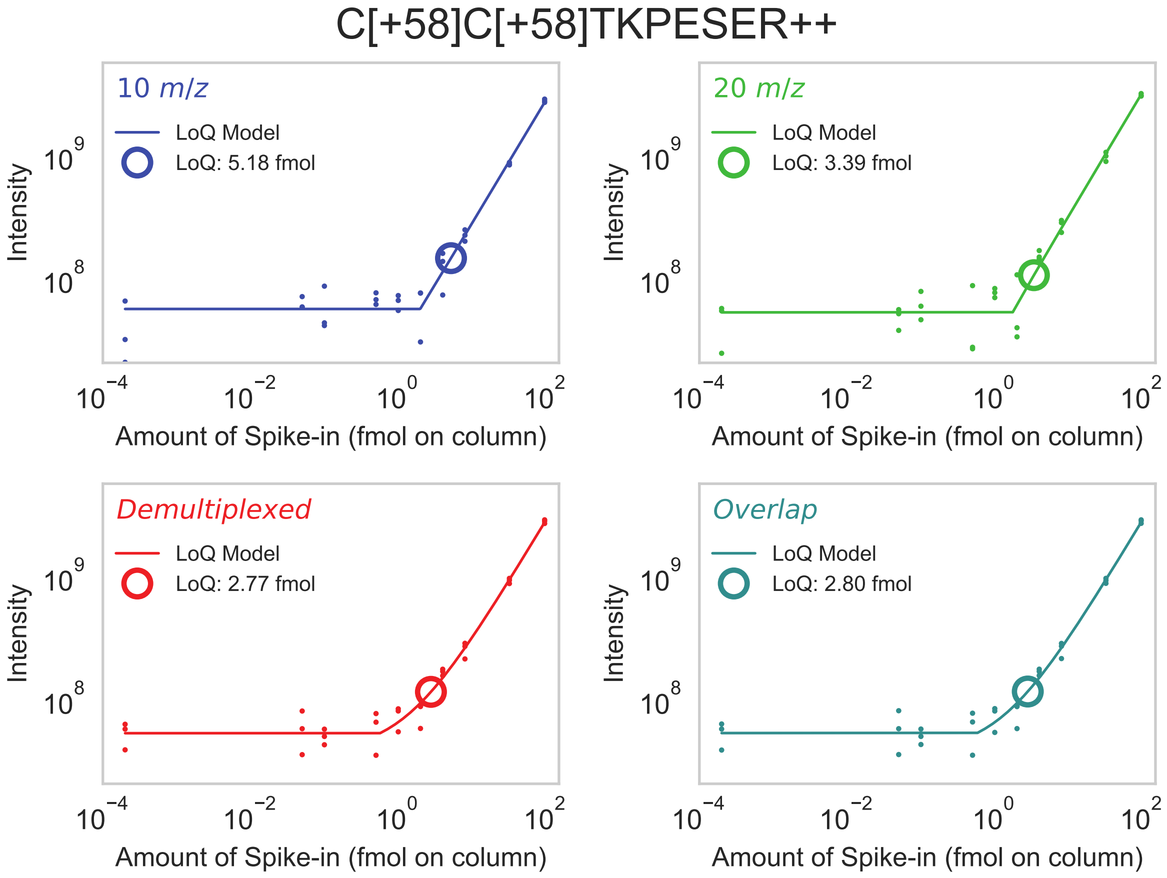
C[+58]C[+58]TKPESER++
10 m/z
LoQ Model
LoQ: 5.18 fmol
20 m/z
LoQ Model
LoQ: 3.39 fmol
Demultiplexed
LoQ Model
LoQ: 2.77 fmol
Overlap
LoQ Model
LoQ: 2.80 fmol
Intensity
10^8
10^9
Amount of Spike-in (fmol on column)
10^{-4}
10^{-2}
10^0
10^2

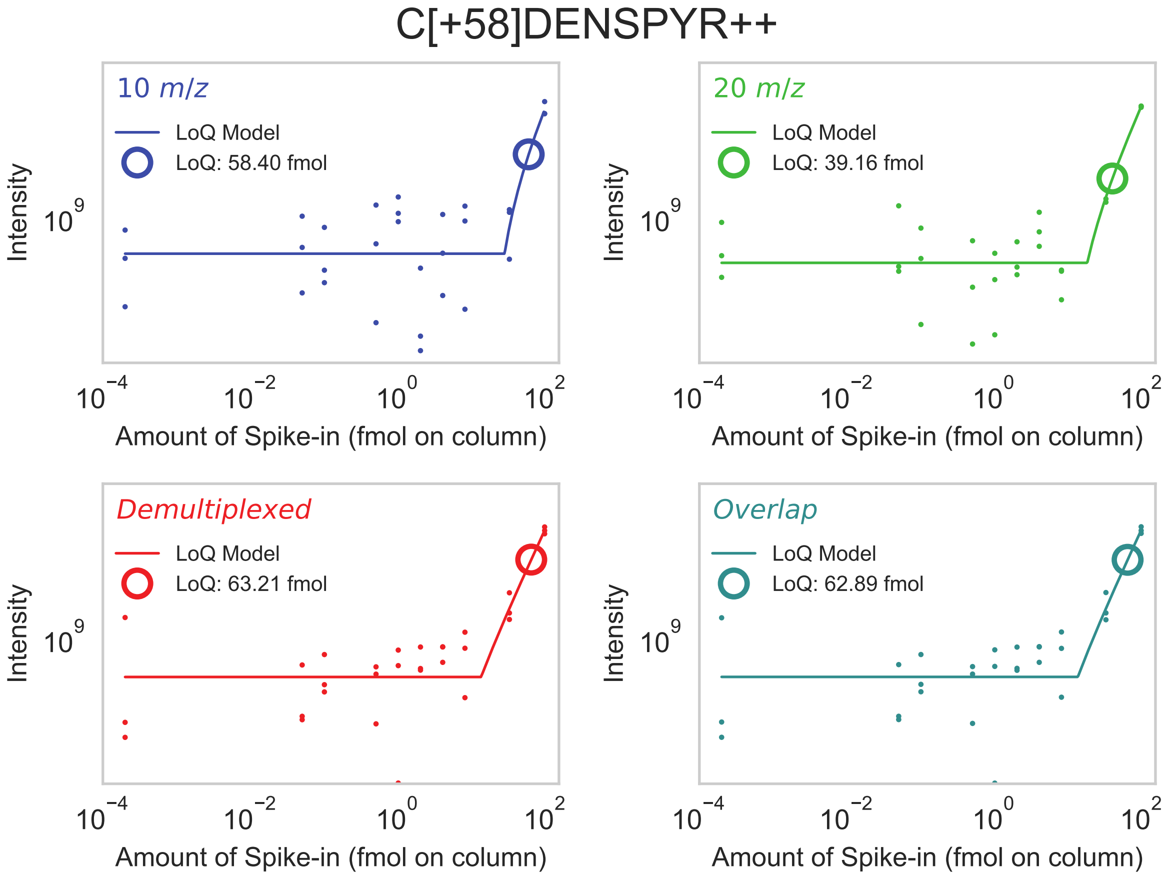
C[+58]DENSPYR++
10 m/z
LoQ Model
LoQ: 58.40 fmol
20 m/z
LoQ Model
LoQ: 39.16 fmol
Demultiplexed
LoQ Model
LoQ: 63.21 fmol
Overlap
LoQ Model
LoQ: 62.89 fmol
Intensity
10^9
10^{-4}
10^{-2}
10^0
10^2
Amount of Spike-in (fmol on column)

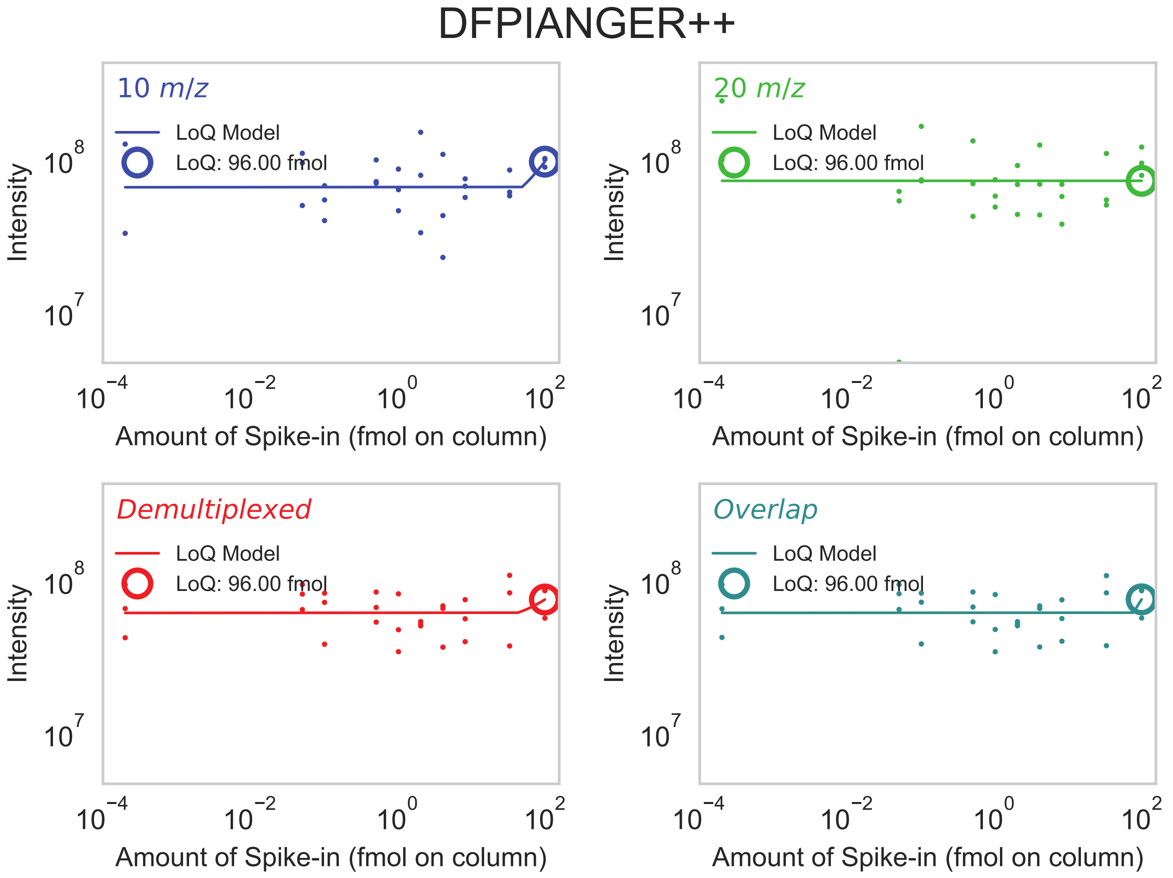
DFPIANGER++
10 m/z
20 m/z
Demultiplexed
Overlap
LoQ Model
LoQ: 96.00 fmol
Intensity
Amount of Spike-in (fmol on column)
10^{8}
10^{7}
10^{-4}
10^{-2}
10^{0}
10^{2}

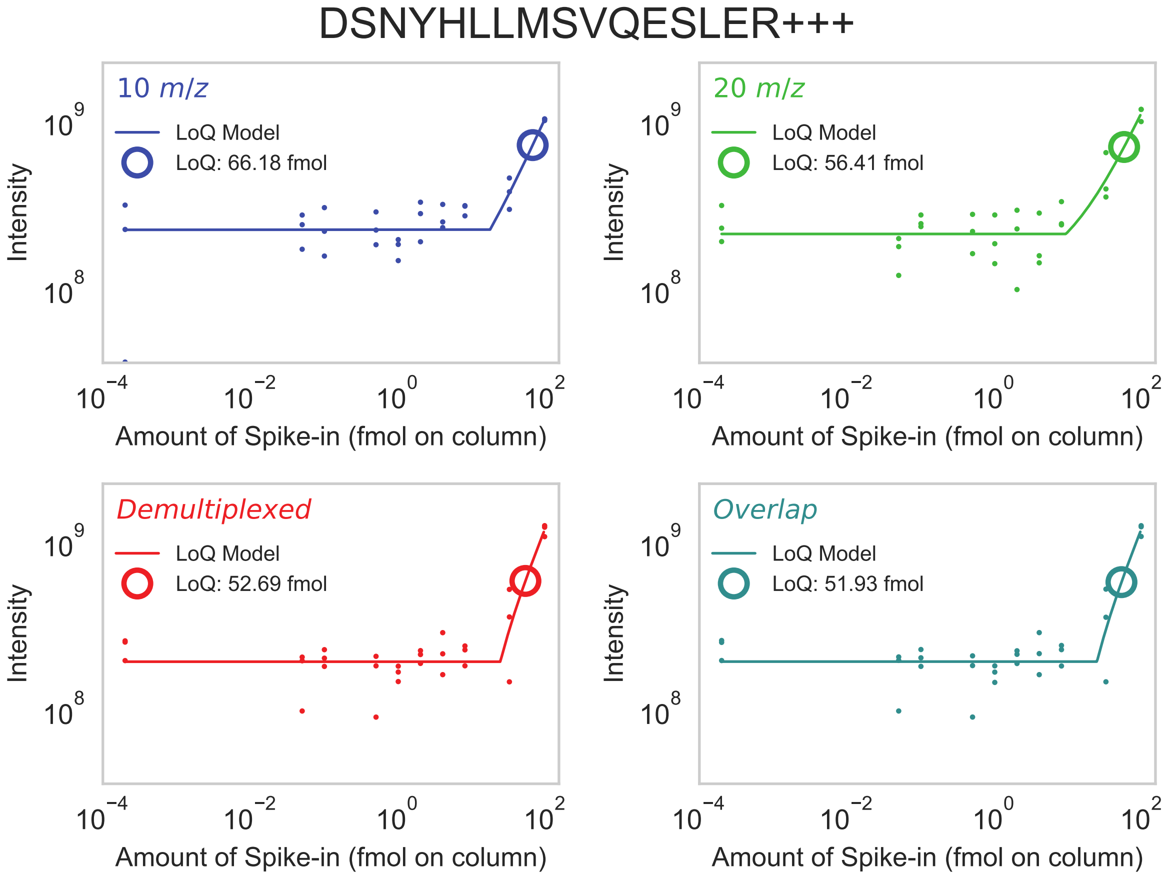

DSNYHLLMSVQESLER+++
10 m/z
LoQ Model
LoQ: 66.18 fmol
20 m/z
LoQ Model
LoQ: 56.41 fmol
Demultiplexed
LoQ Model
LoQ: 52.69 fmol
Overlap
LoQ Model
LoQ: 51.93 fmol
Intensity
Amount of Spike-in (fmol on column)

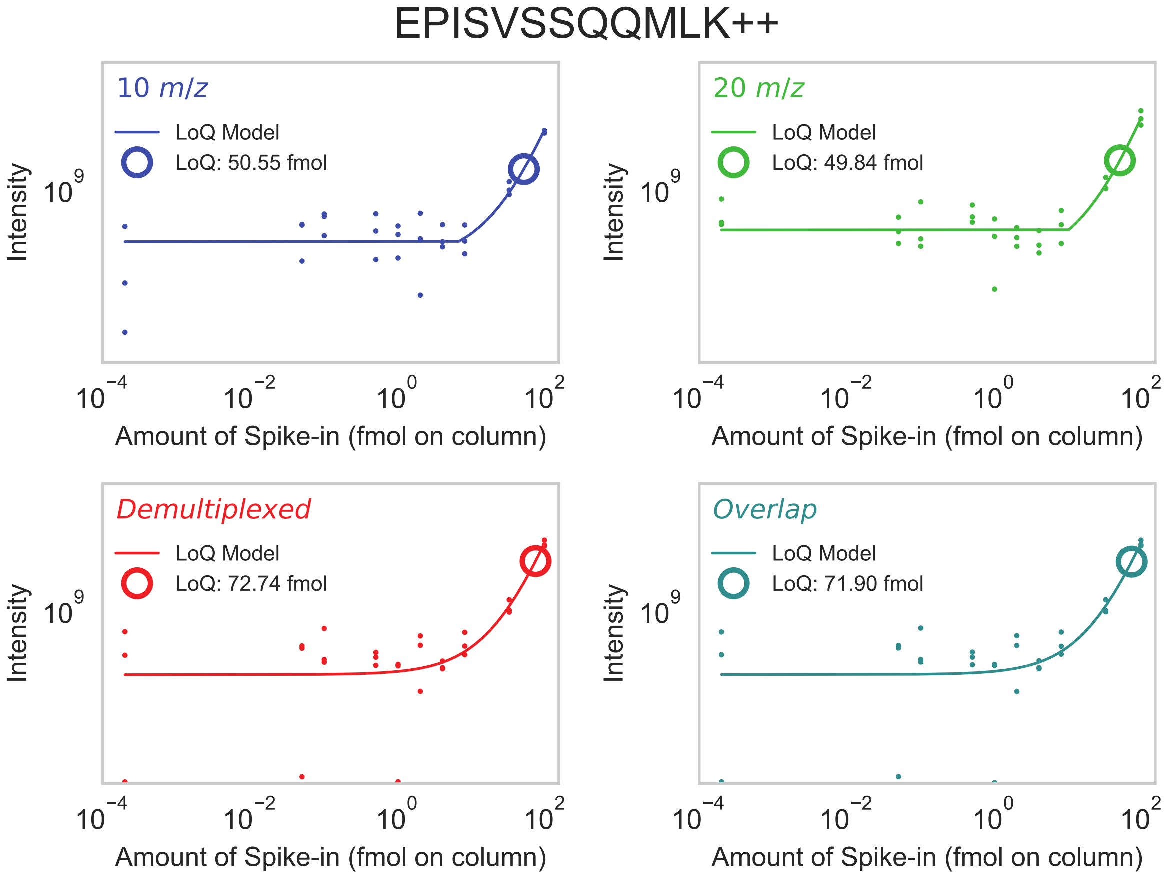
EPISVSSQQMLK++
10 m/z
LoQ Model
LoQ: 50.55 fmol
20 m/z
LoQ Model
LoQ: 49.84 fmol
Demultiplexed
LoQ Model
LoQ: 72.74 fmol
Overlap
LoQ Model
LoQ: 71.90 fmol
Intensity
10^9
10^-4 10^-2 10^0 10^2
Amount of Spike-in (fmol on column)

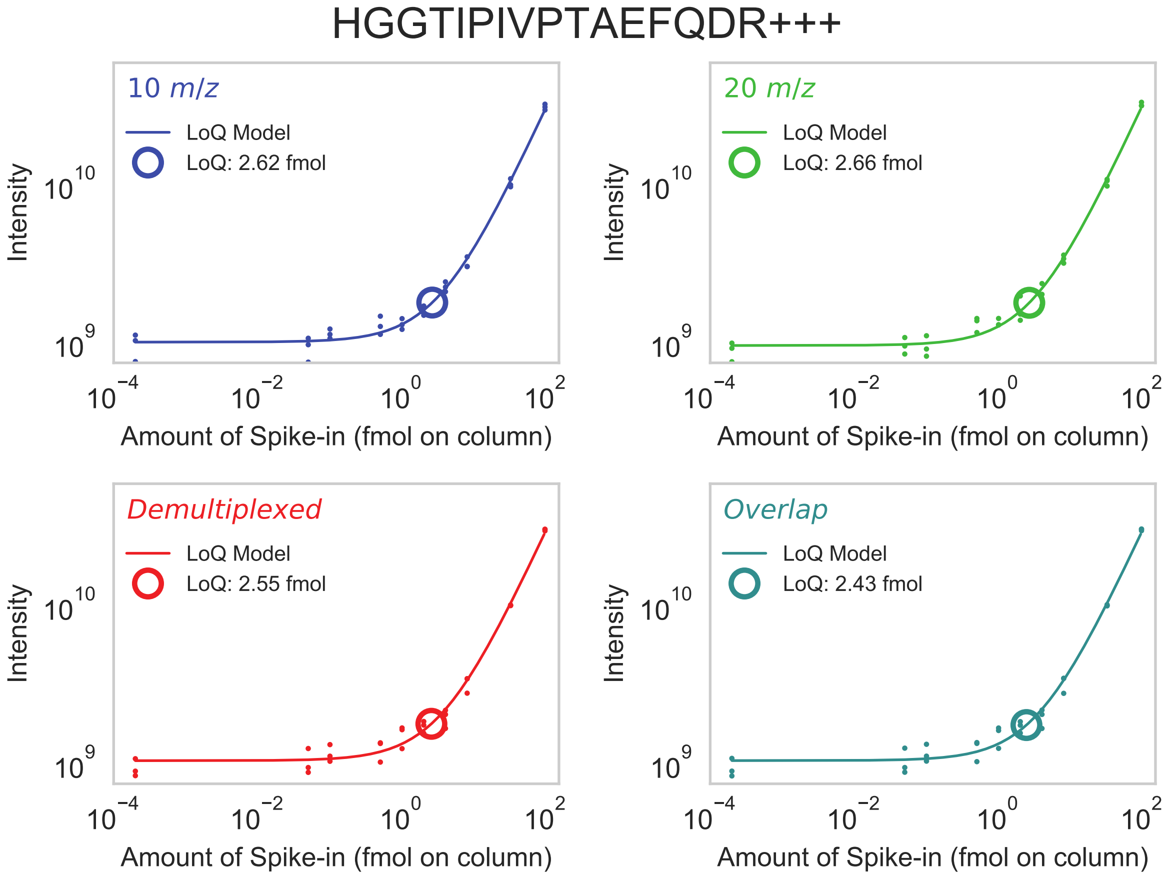
HGGTIPIVPTAEFQDR+++
10 m/z
LoQ Model
LoQ: 2.62 fmol
20 m/z
LoQ Model
LoQ: 2.66 fmol
Demultiplexed
LoQ Model
LoQ: 2.55 fmol
Overlap
LoQ Model
LoQ: 2.43 fmol
Intensity
10^{9}
10^{10}
10^{-4}
10^{-2}
10^{0}
10^{2}
Amount of Spike-in (fmol on column)

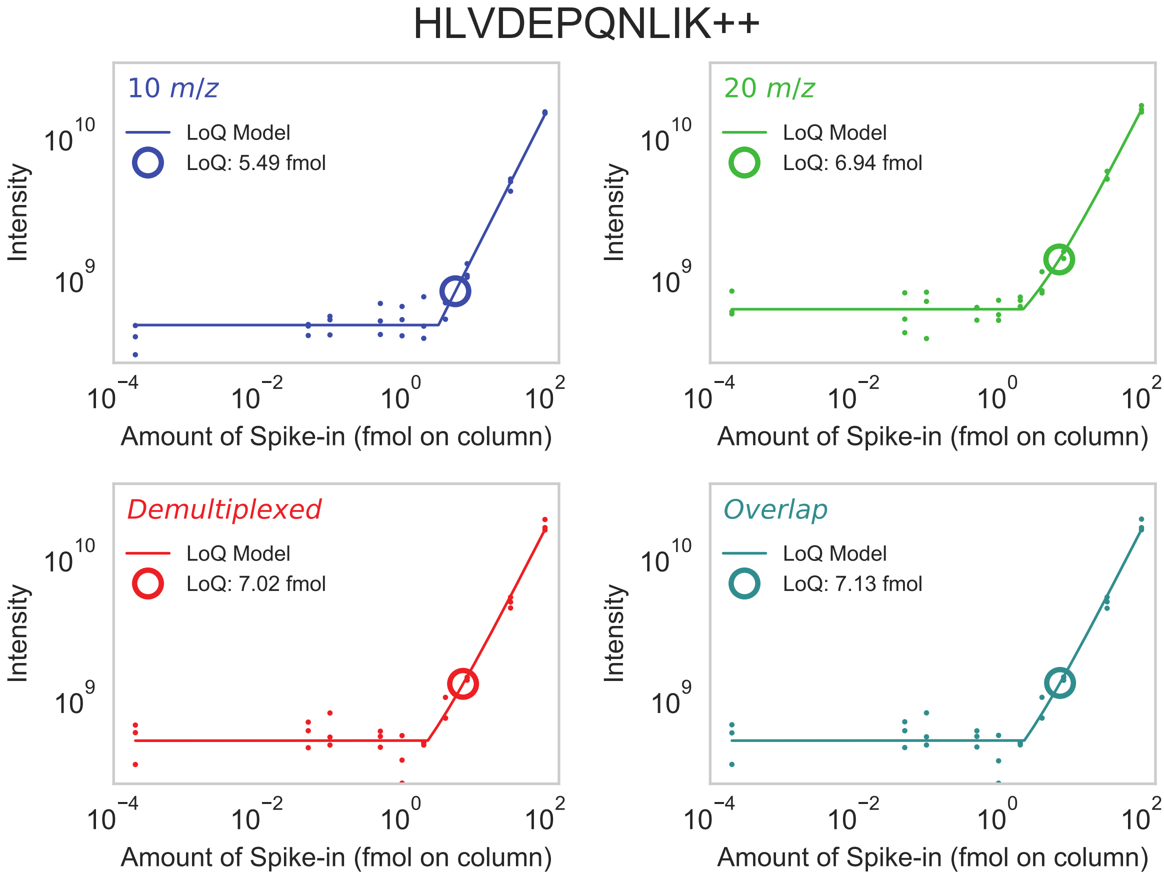

HLVDEPQNLIK++
10 m/z
LoQ Model
LoQ: 5.49 fmol
20 m/z
LoQ Model
LoQ: 6.94 fmol
Demultiplexed
LoQ Model
LoQ: 7.02 fmol
Overlap
LoQ Model
LoQ: 7.13 fmol
Intensity
Amount of Spike-in (fmol on column)

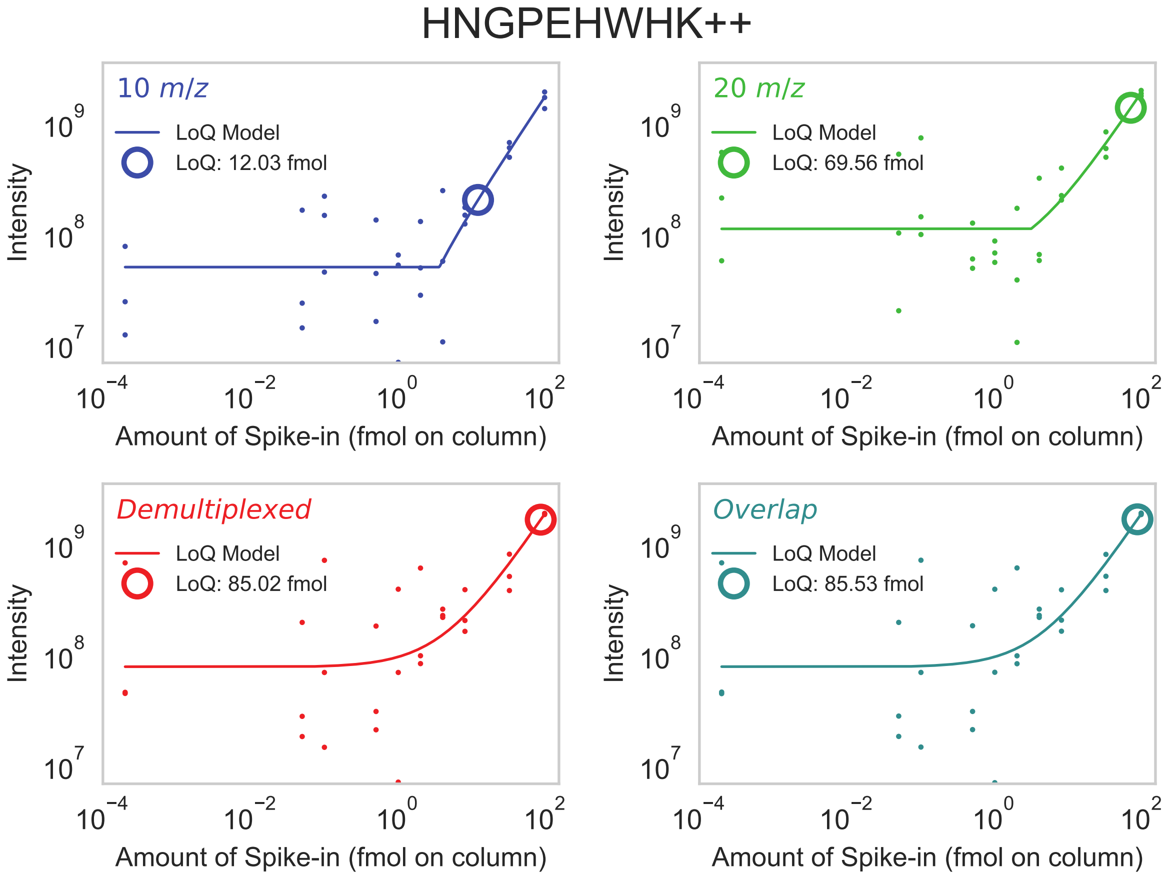

HNGPEHWHK++
10 m/z
LoQ Model
LoQ: 12.03 fmol
20 m/z
LoQ Model
LoQ: 69.56 fmol
Demultiplexed
LoQ Model
LoQ: 85.02 fmol
Overlap
LoQ Model
LoQ: 85.53 fmol
Intensity
Amount of Spike-in (fmol on column)

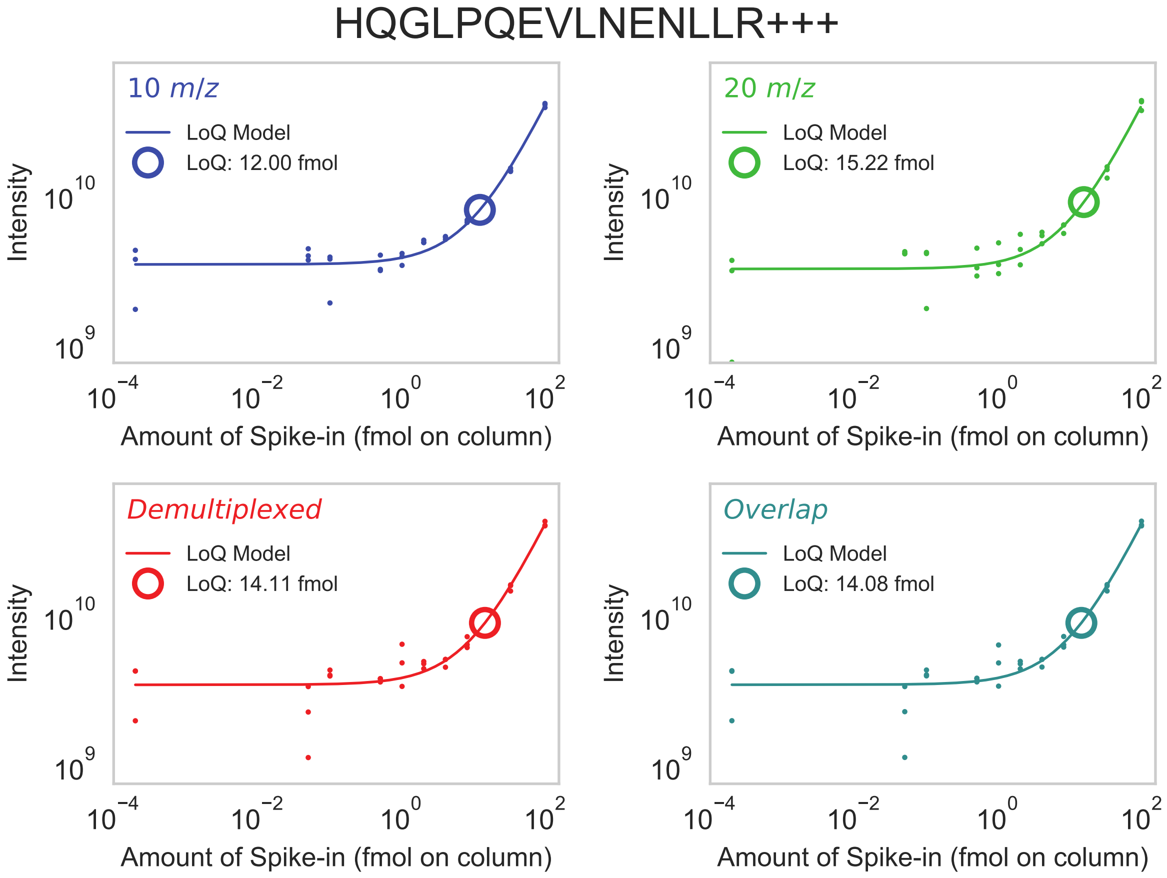

HQGLPQEVLNENLLR+++
10 m/z
LoQ Model
LoQ: 12.00 fmol
20 m/z
LoQ Model
LoQ: 15.22 fmol
Demultiplexed
LoQ Model
LoQ: 14.11 fmol
Overlap
LoQ Model
LoQ: 14.08 fmol
Intensity
10^{9}
10^{10}
10^{-4}
10^{-2}
10^{0}
10^{2}
Amount of Spike-in (fmol on column)

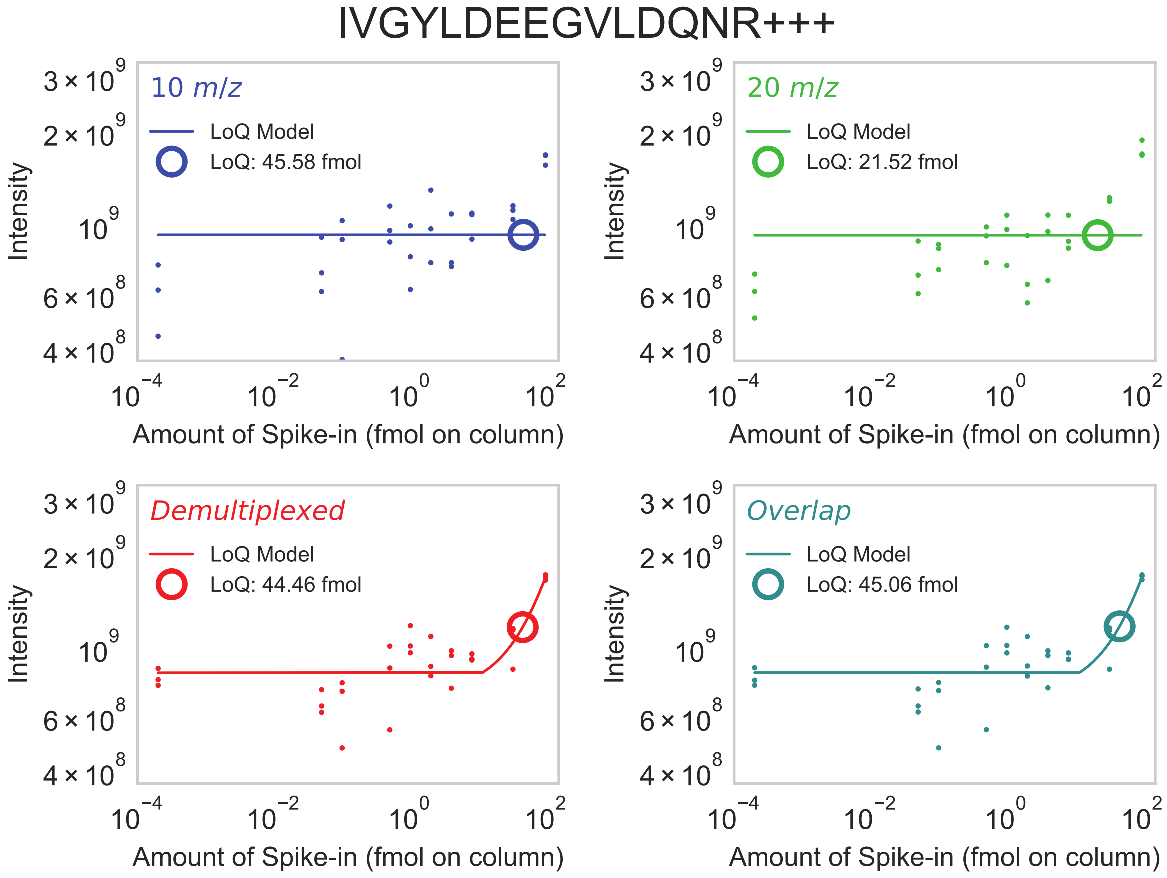

IVGYLDEEGVLDQNR+++
10 m/z
LoQ Model
LoQ: 45.58 fmol
20 m/z
LoQ Model
LoQ: 21.52 fmol
Demultiplexed
LoQ Model
LoQ: 44.46 fmol
Overlap
LoQ Model
LoQ: 45.06 fmol
Intensity
Amount of Spike-in (fmol on column)

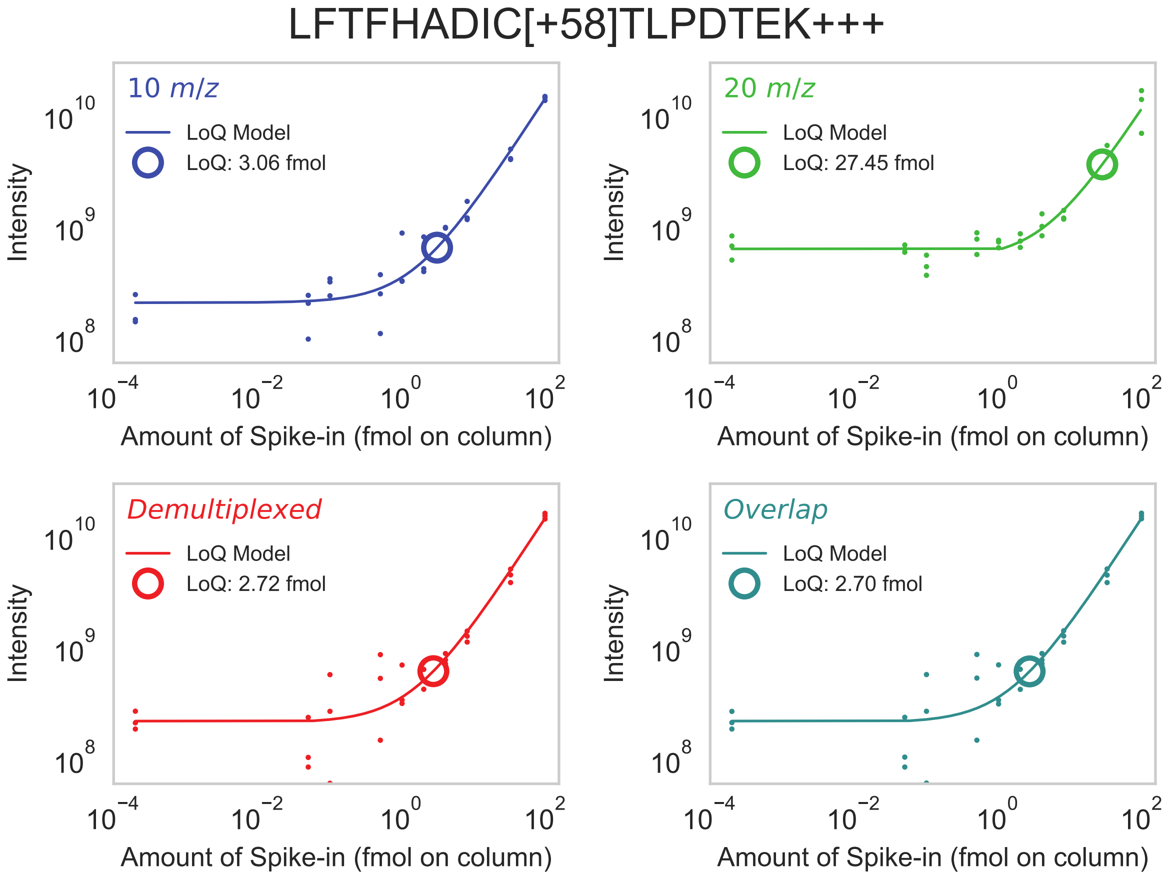
LFTFHADIC[+58]TLPDTEK+++
10 m/z
LoQ Model
LoQ: 3.06 fmol
20 m/z
LoQ Model
LoQ: 27.45 fmol
Demultiplexed
LoQ Model
LoQ: 2.72 fmol
Overlap
LoQ Model
LoQ: 2.70 fmol
Intensity
Amount of Spike-in (fmol on column)

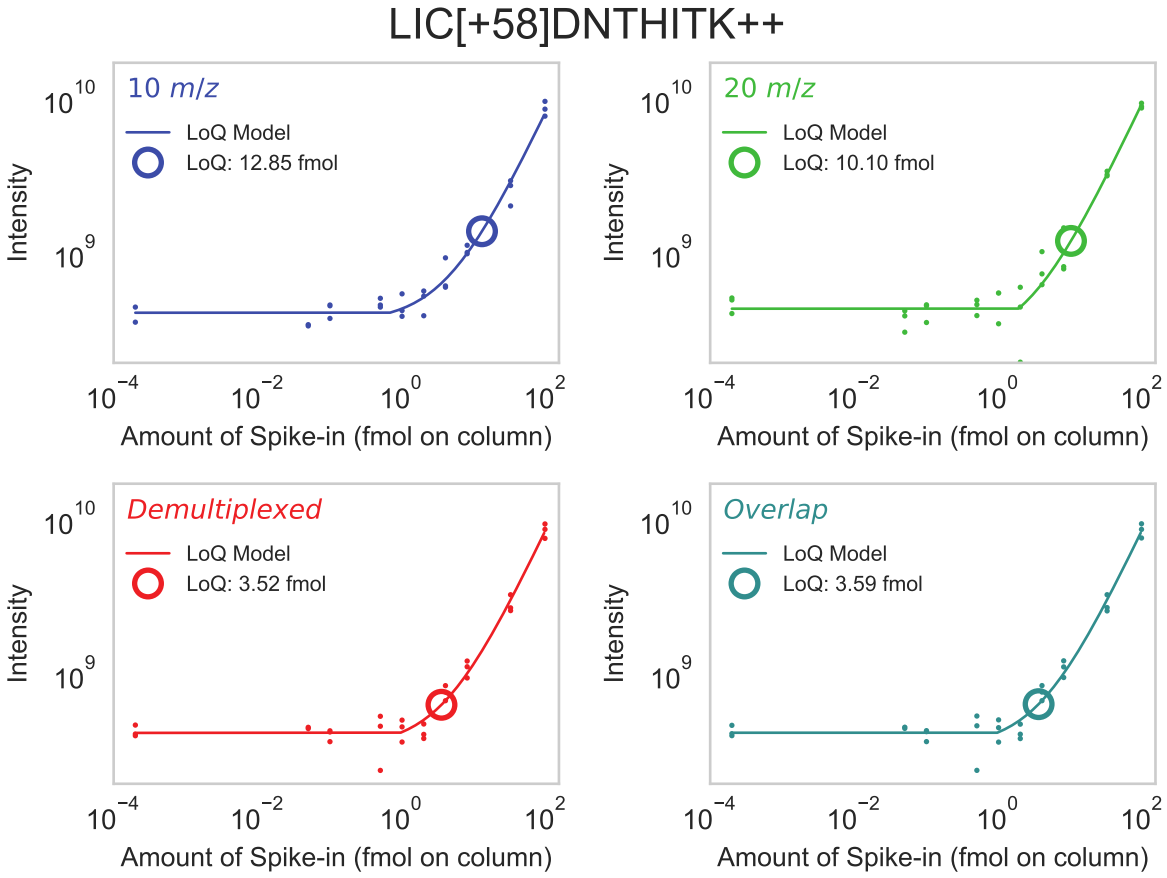

LIC[+58]DNTHITK++
10 m/z
LoQ Model
LoQ: 12.85 fmol
20 m/z
LoQ Model
LoQ: 10.10 fmol
Demultiplexed
LoQ Model
LoQ: 3.52 fmol
Overlap
LoQ Model
LoQ: 3.59 fmol
Intensity
Amount of Spike-in (fmol on column)

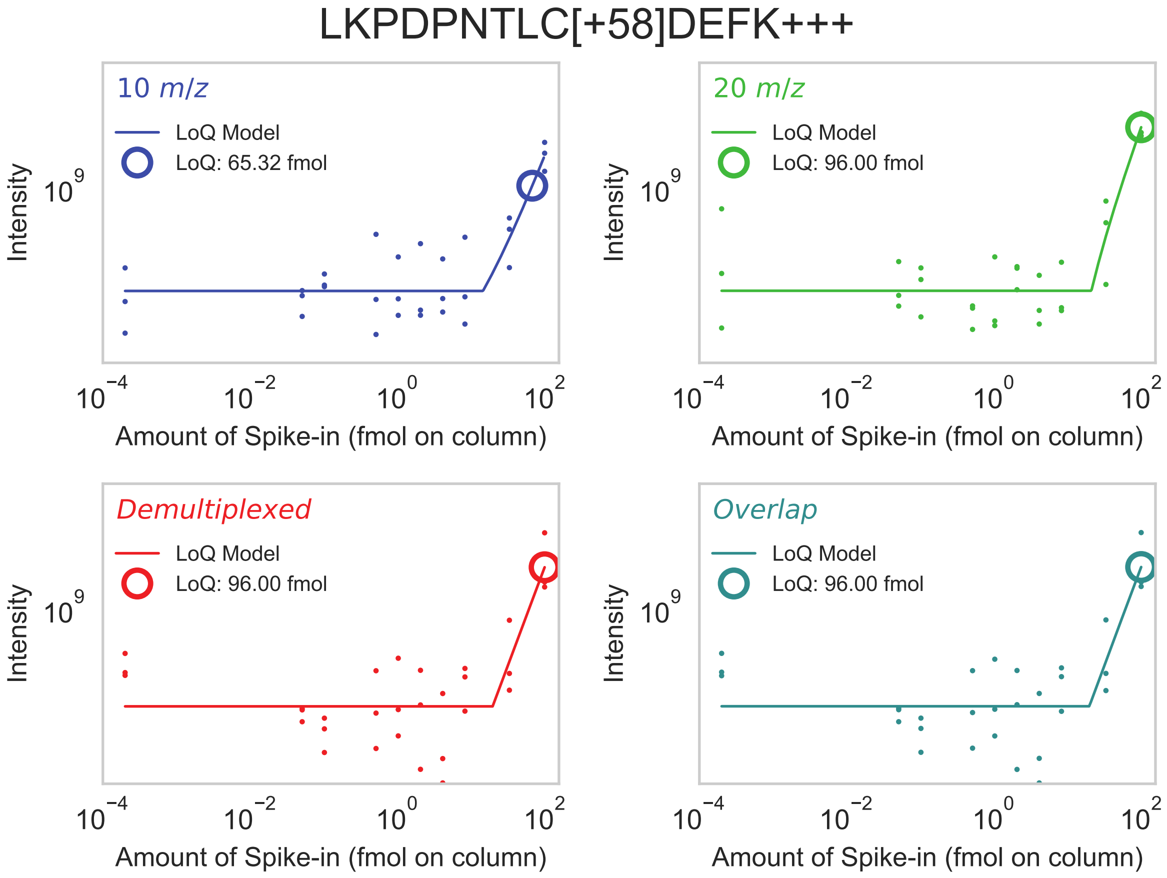

LKPDPNTLC[+58]DEFK+++
10 m/z
LoQ Model
LoQ: 65.32 fmol
20 m/z
LoQ Model
LoQ: 96.00 fmol
Demultiplexed
LoQ Model
LoQ: 96.00 fmol
Overlap
LoQ Model
LoQ: 96.00 fmol
Intensity
10^9
10^{-4} 10^{-2} 10^0 10^2
Amount of Spike-in (fmol on column)

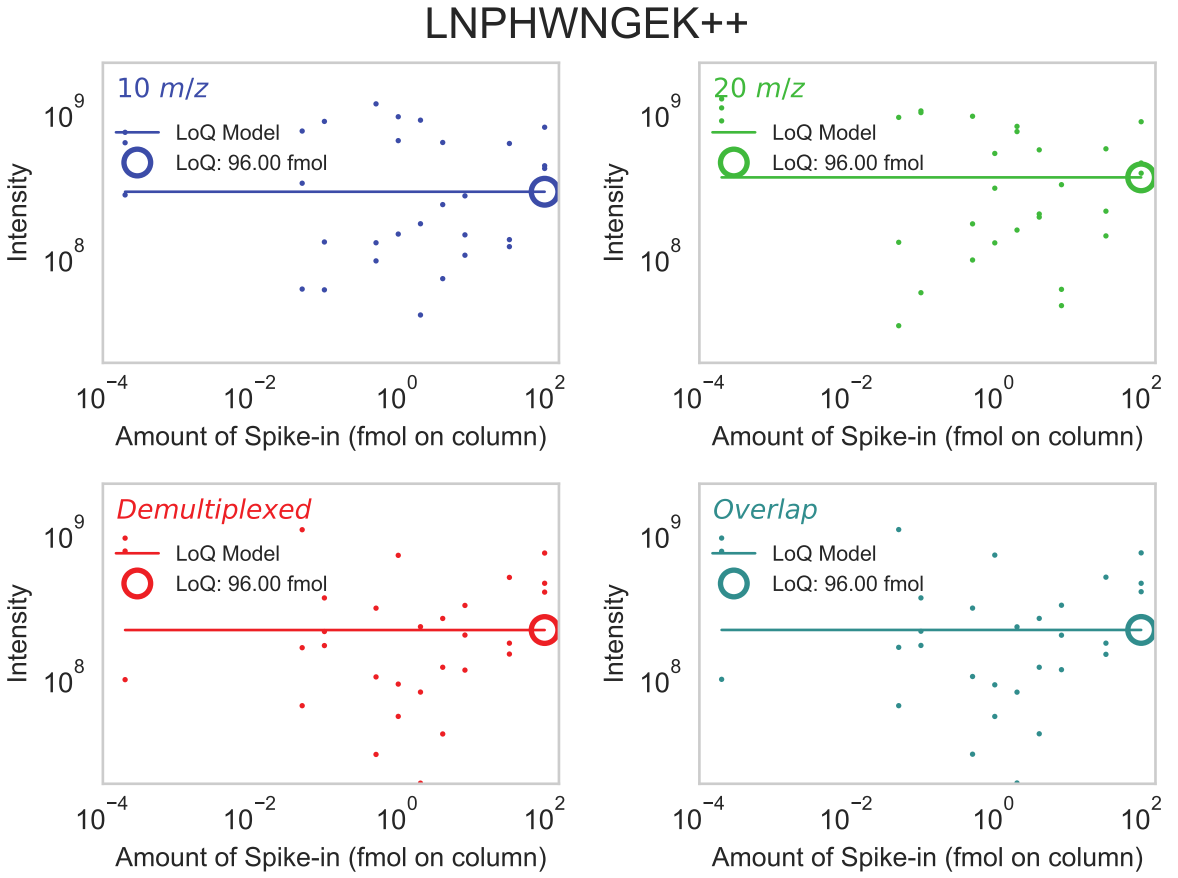

LNPHWNGEK++
10 m/z
20 m/z
Demultiplexed
Overlap
LoQ Model
LoQ: 96.00 fmol
Intensity
10^8
10^9
10^{-4}
10^{-2}
10^0
10^2
Amount of Spike-in (fmol on column)

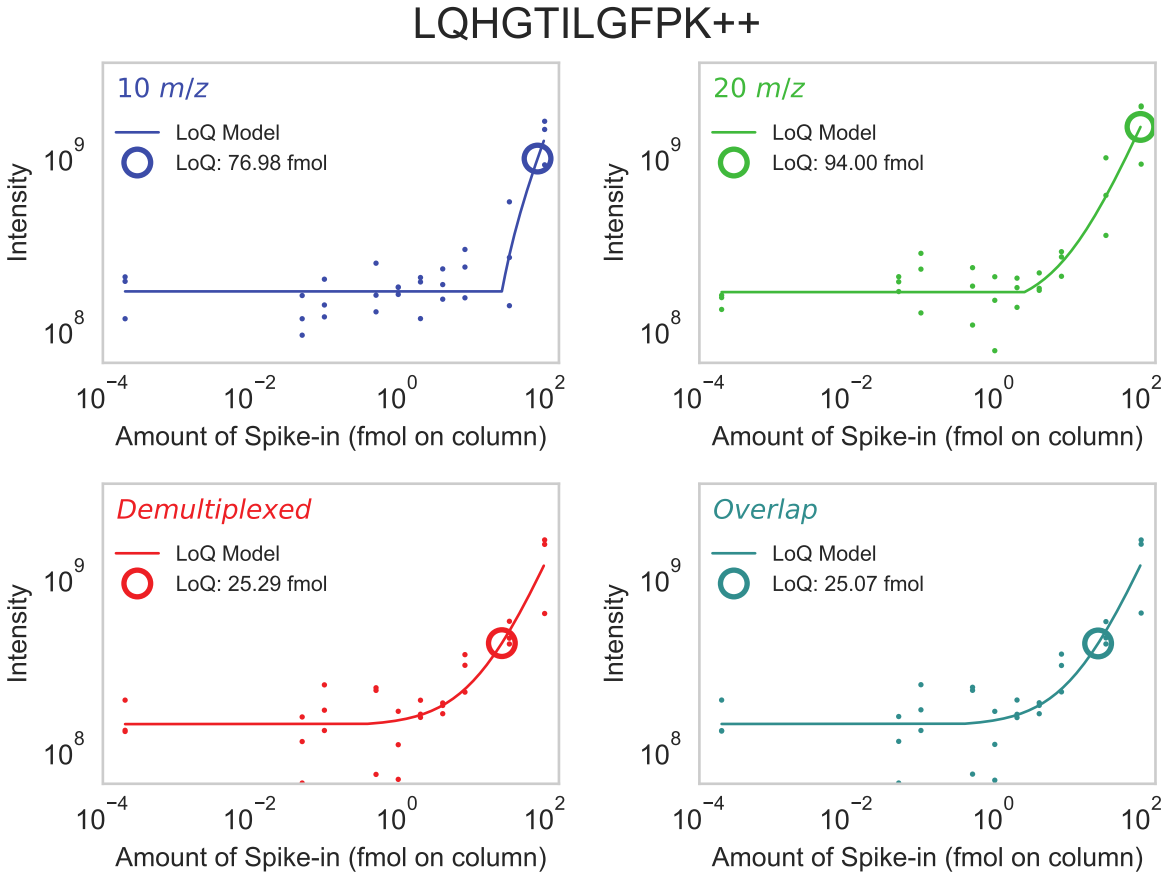

LQHGTILGFPK++
10 m/z
LoQ Model
LoQ: 76.98 fmol
20 m/z
LoQ Model
LoQ: 94.00 fmol
Demultiplexed
LoQ Model
LoQ: 25.29 fmol
Overlap
LoQ Model
LoQ: 25.07 fmol
Intensity
Amount of Spike-in (fmol on column)

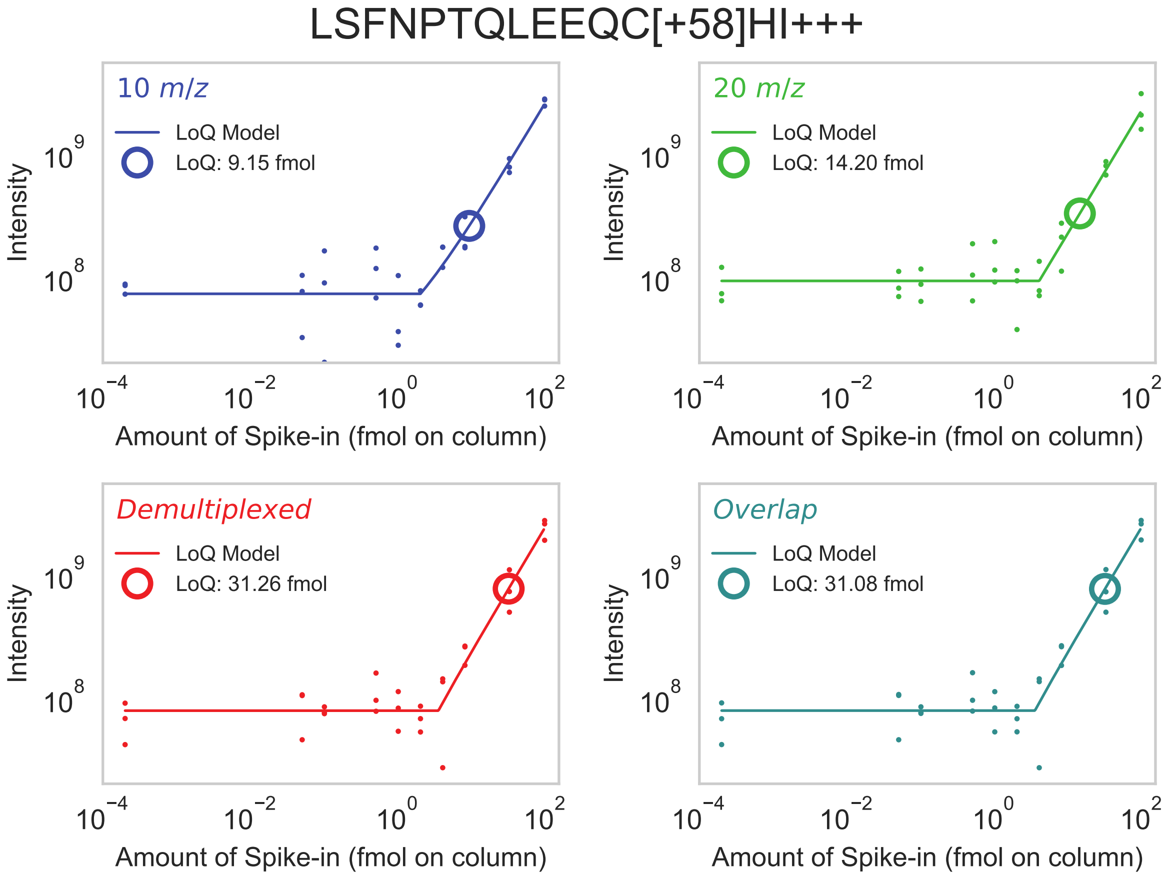
LSFNPTQLEEQC[+58]HI+++
10 m/z
LoQ Model
LoQ: 9.15 fmol
20 m/z
LoQ Model
LoQ: 14.20 fmol
Demultiplexed
LoQ Model
LoQ: 31.26 fmol
Overlap
LoQ Model
LoQ: 31.08 fmol
Intensity
Amount of Spike-in (fmol on column)
10^{8}
10^{9}
10^{-4}
10^{-2}
10^{0}
10^{2}

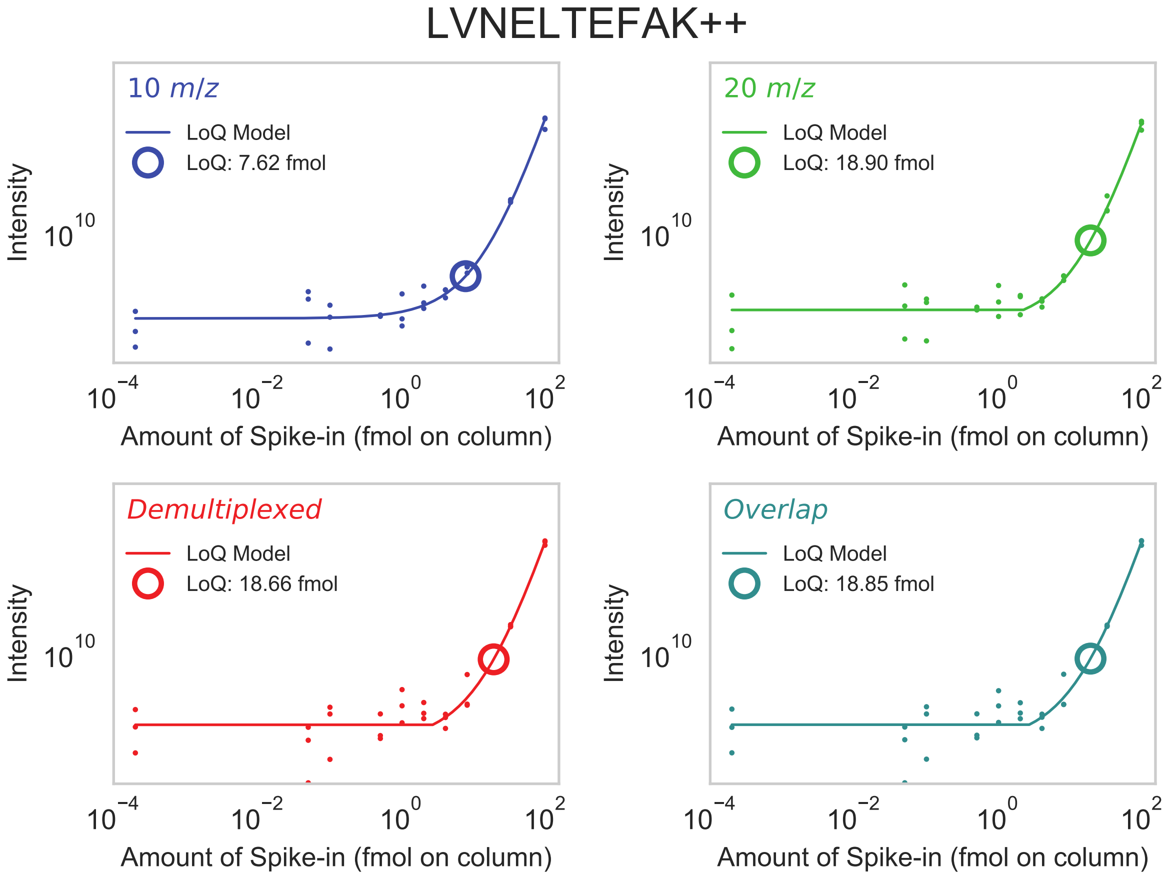
LVNELTEFAK++
10 m/z
LoQ Model
LoQ: 7.62 fmol
20 m/z
LoQ Model
LoQ: 18.90 fmol
Demultiplexed
LoQ Model
LoQ: 18.66 fmol
Overlap
LoQ Model
LoQ: 18.85 fmol
Intensity
10^{10}
10^{-4}
10^{-2}
10^{0}
10^{2}
Amount of Spike-in (fmol on column)

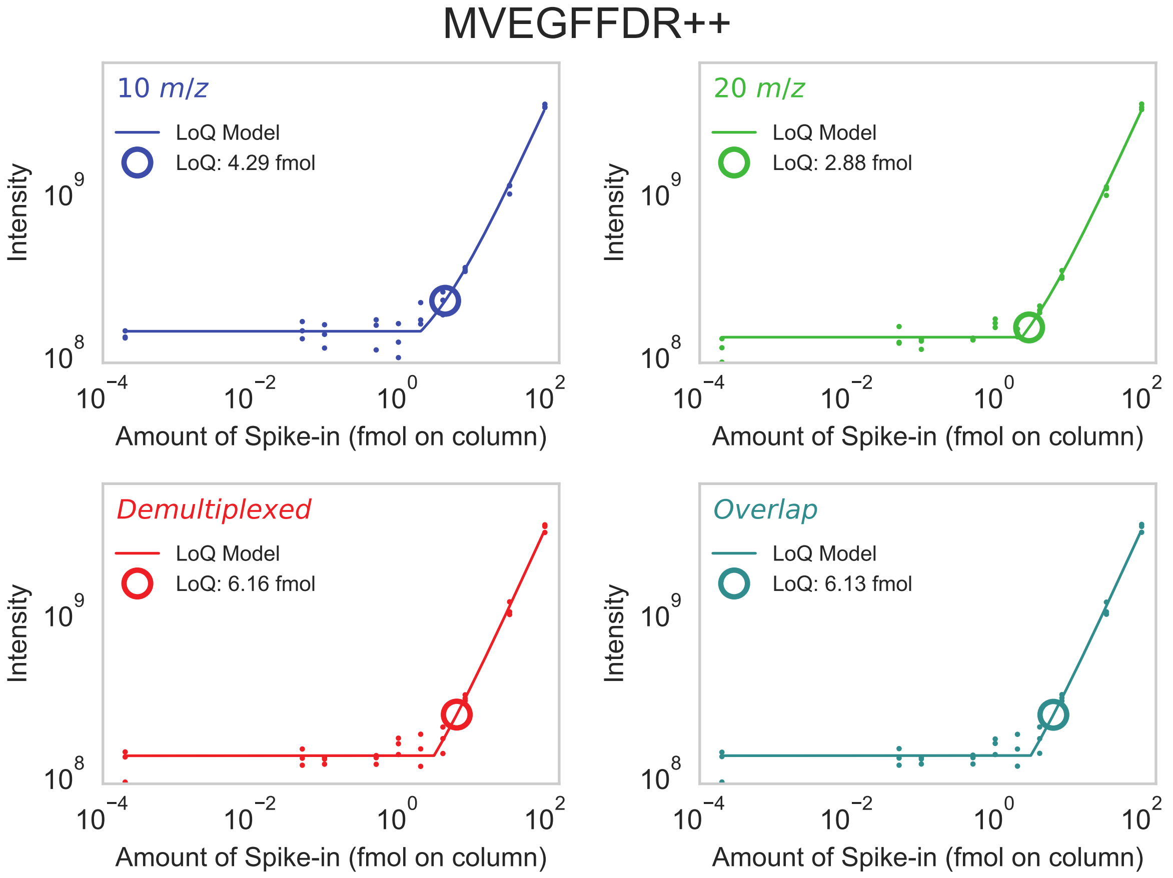
MVEGFFDR++
10 m/z
LoQ Model
LoQ: 4.29 fmol
20 m/z
LoQ Model
LoQ: 2.88 fmol
Demultiplexed
LoQ Model
LoQ: 6.16 fmol
Overlap
LoQ Model
LoQ: 6.13 fmol
Intensity
Amount of Spike-in (fmol on column)

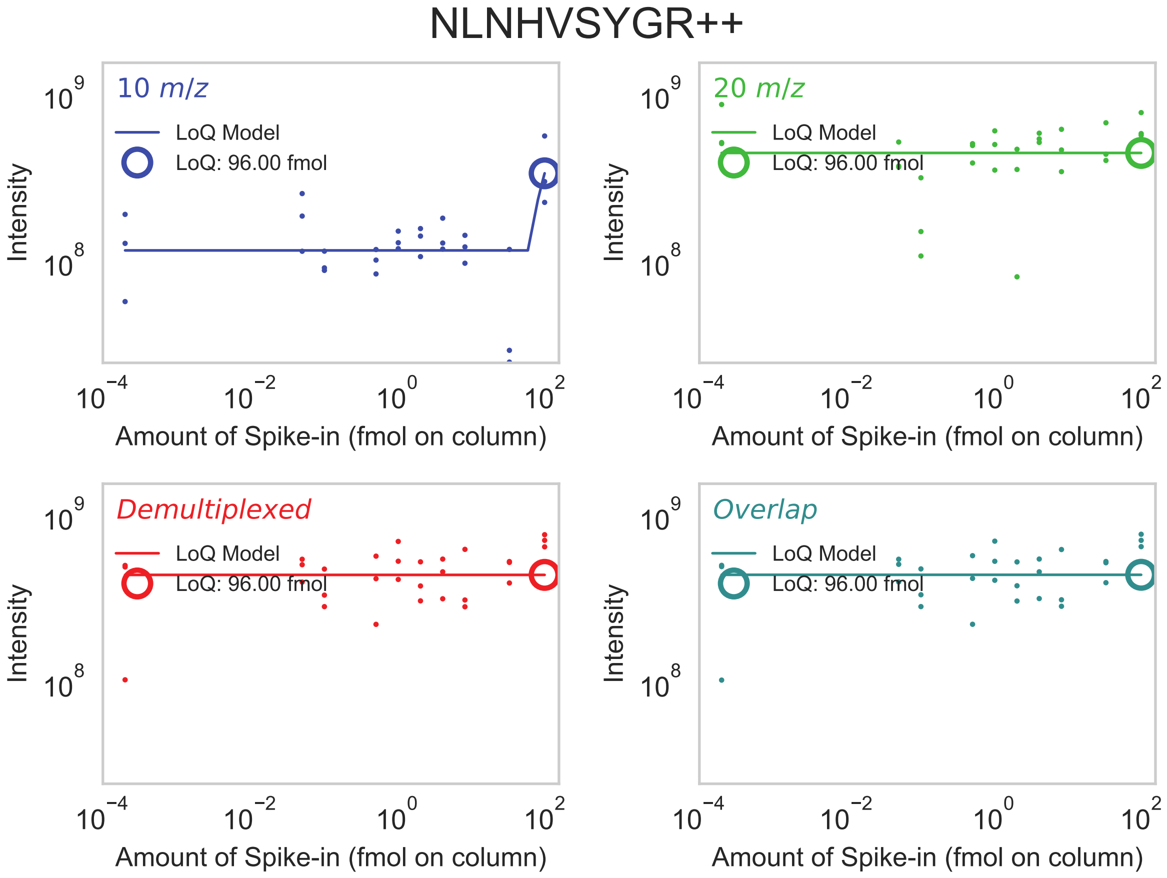
NLNHVSYGR++
10 m/z
20 m/z
Demultiplexed
Overlap
LoQ Model
LoQ: 96.00 fmol
Intensity
Amount of Spike-in (fmol on column)
10^{8}
10^{9}
10^{-4}
10^{-2}
10^{0}
10^{2}

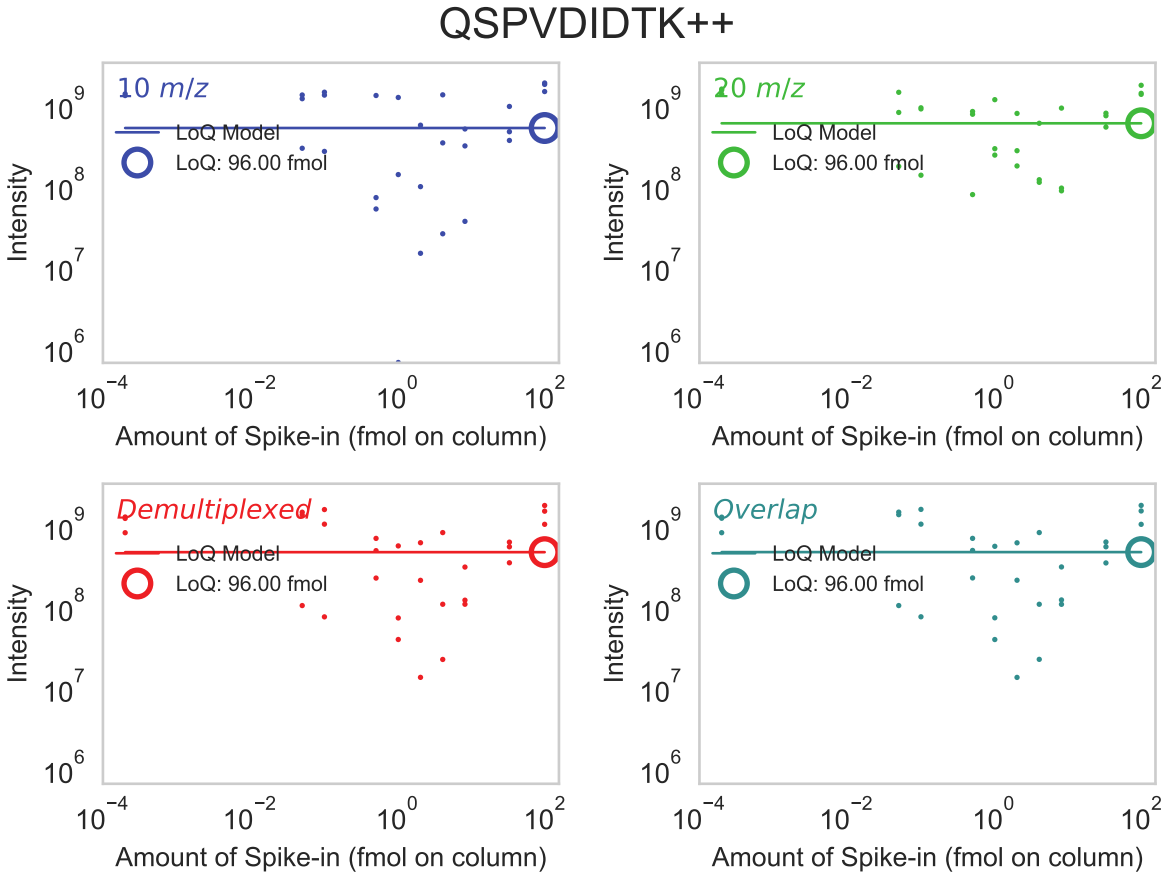
QSPVDIDTK++
10 m/z
20 m/z
Demultiplexed
Overlap
LoQ Model
LoQ: 96.00 fmol
Intensity
Amount of Spike-in (fmol on column)
10^{6}
10^{7}
10^{8}
10^{9}
10^{-4}
10^{-2}
10^{0}
10^{2}

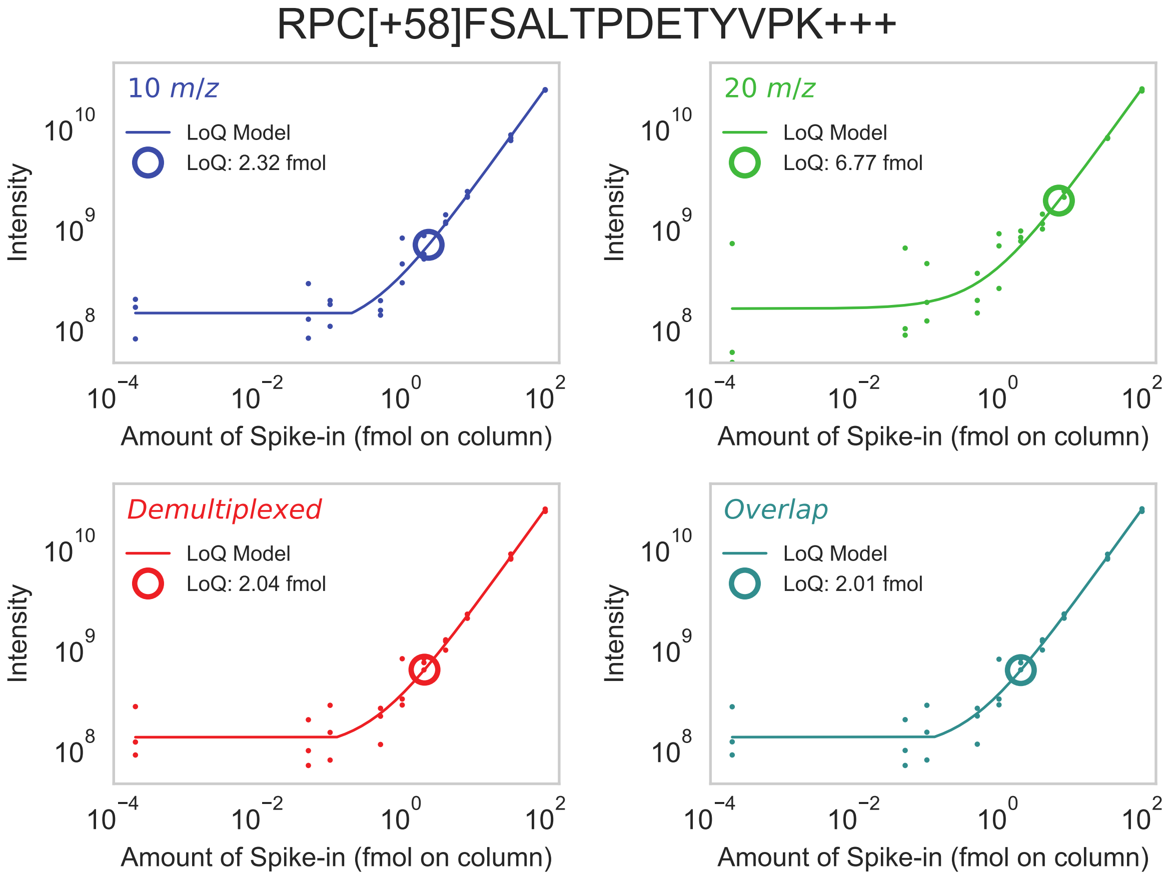
RPC[+58]FSALTPDETYVPK+++
10 m/z
LoQ Model
LoQ: 2.32 fmol
20 m/z
LoQ Model
LoQ: 6.77 fmol
Demultiplexed
LoQ Model
LoQ: 2.04 fmol
Overlap
LoQ Model
LoQ: 2.01 fmol
Intensity
Amount of Spike-in (fmol on column)
10^{8}
10^{9}
10^{10}
10^{-4}
10^{-2}
10^{0}
10^{2}

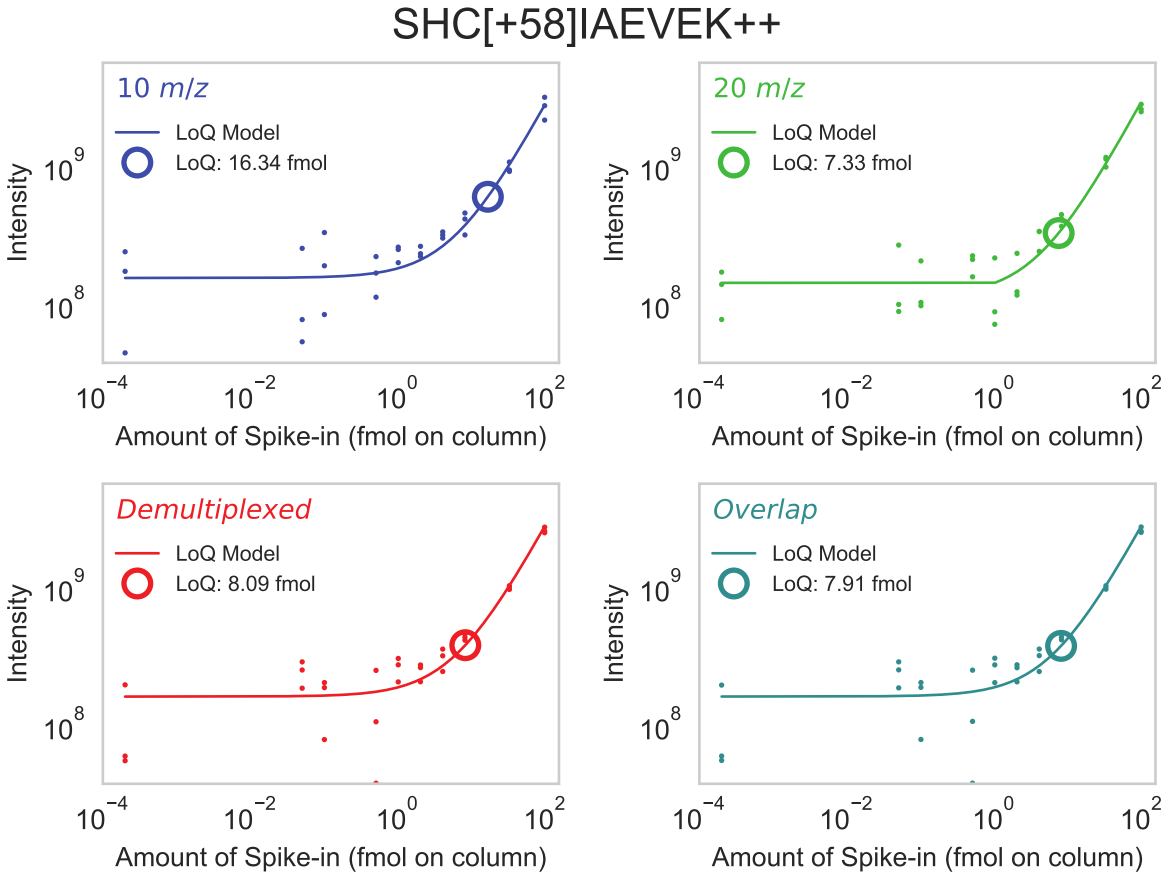
SHC[+58]IAEVEK++
10 m/z
LoQ Model
LoQ: 16.34 fmol
20 m/z
LoQ Model
LoQ: 7.33 fmol
Demultiplexed
LoQ Model
LoQ: 8.09 fmol
Overlap
LoQ Model
LoQ: 7.91 fmol
Intensity
Amount of Spike-in (fmol on column)

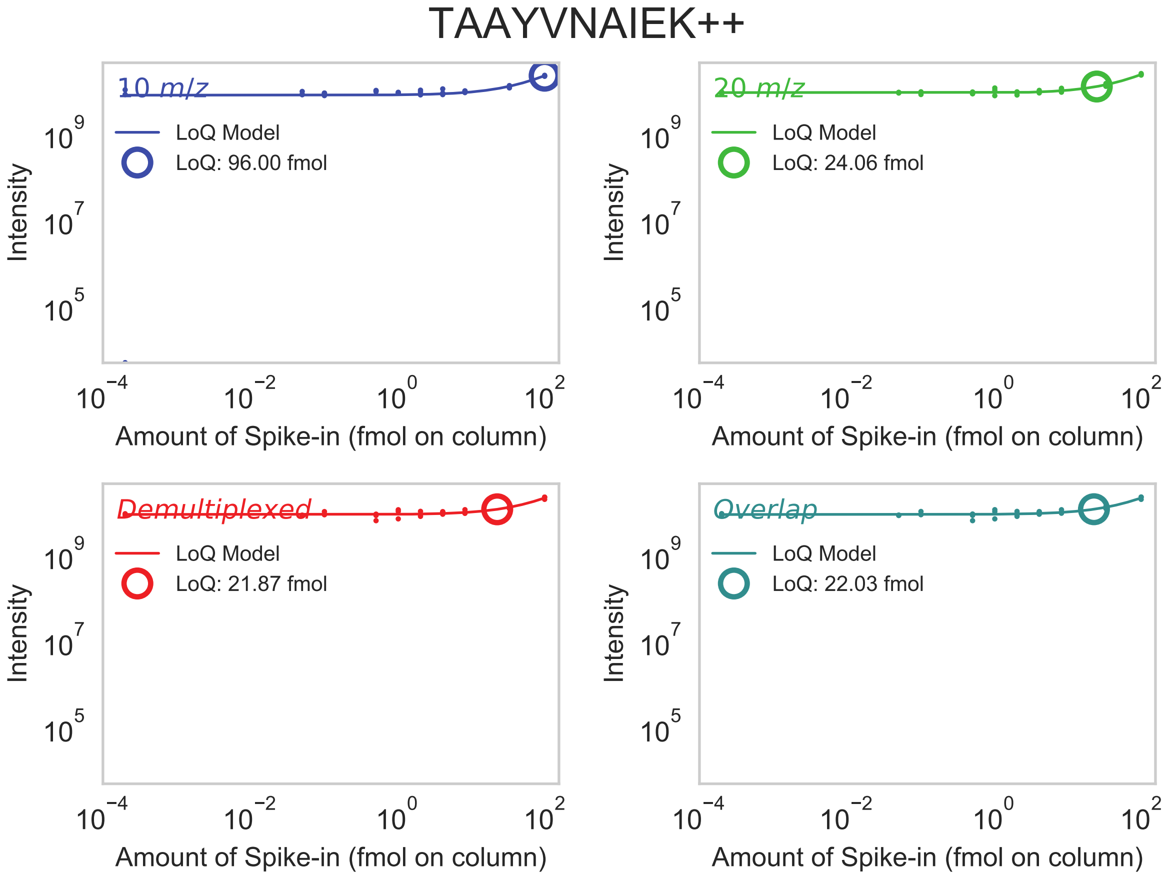

TAAYVNAIEK++
10 m/z
LoQ Model
LoQ: 96.00 fmol
20 m/z
LoQ Model
LoQ: 24.06 fmol
Demultiplexed
LoQ Model
LoQ: 21.87 fmol
Overlap
LoQ Model
LoQ: 22.03 fmol
Intensity
Amount of Spike-in (fmol on column)

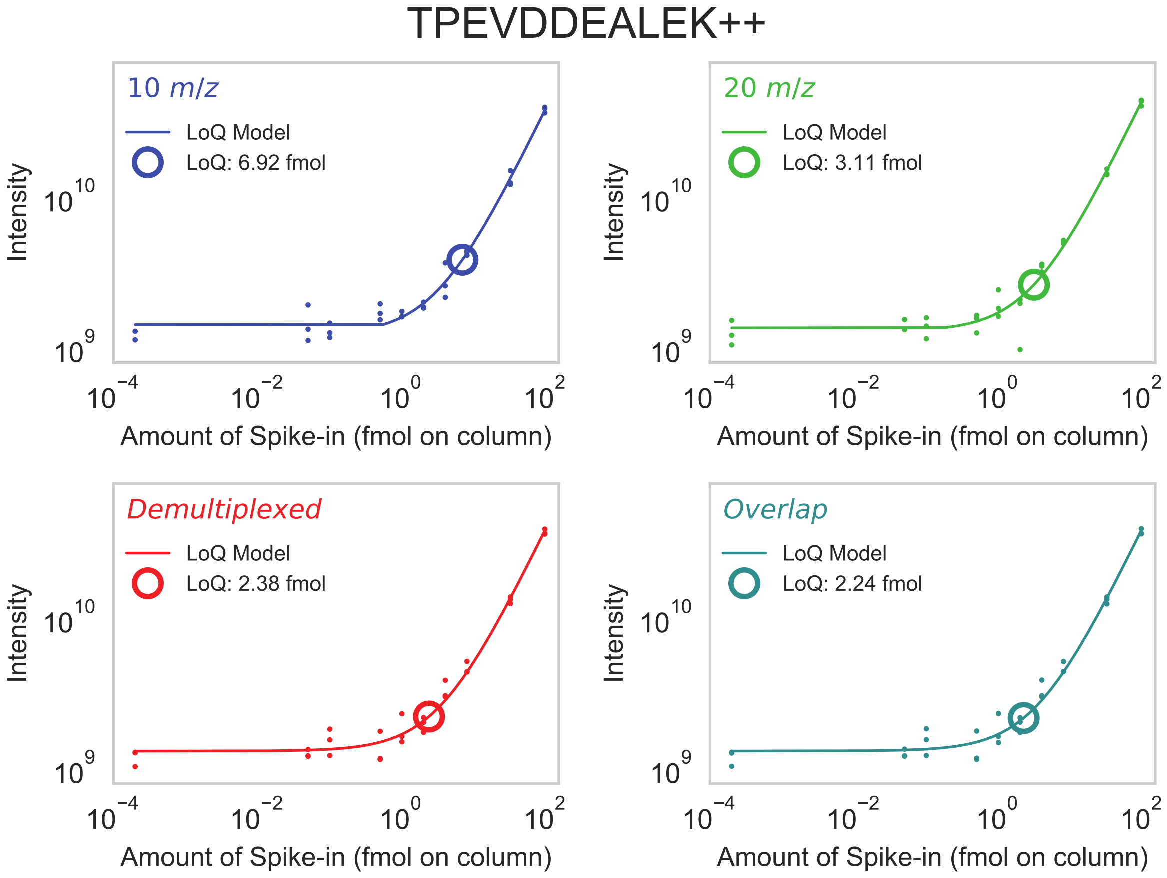

TPEVDDEALEK++
10 m/z
LoQ Model
LoQ: 6.92 fmol
20 m/z
LoQ Model
LoQ: 3.11 fmol
Demultiplexed
LoQ Model
LoQ: 2.38 fmol
Overlap
LoQ Model
LoQ: 2.24 fmol
Intensity
Amount of Spike-in (fmol on column)

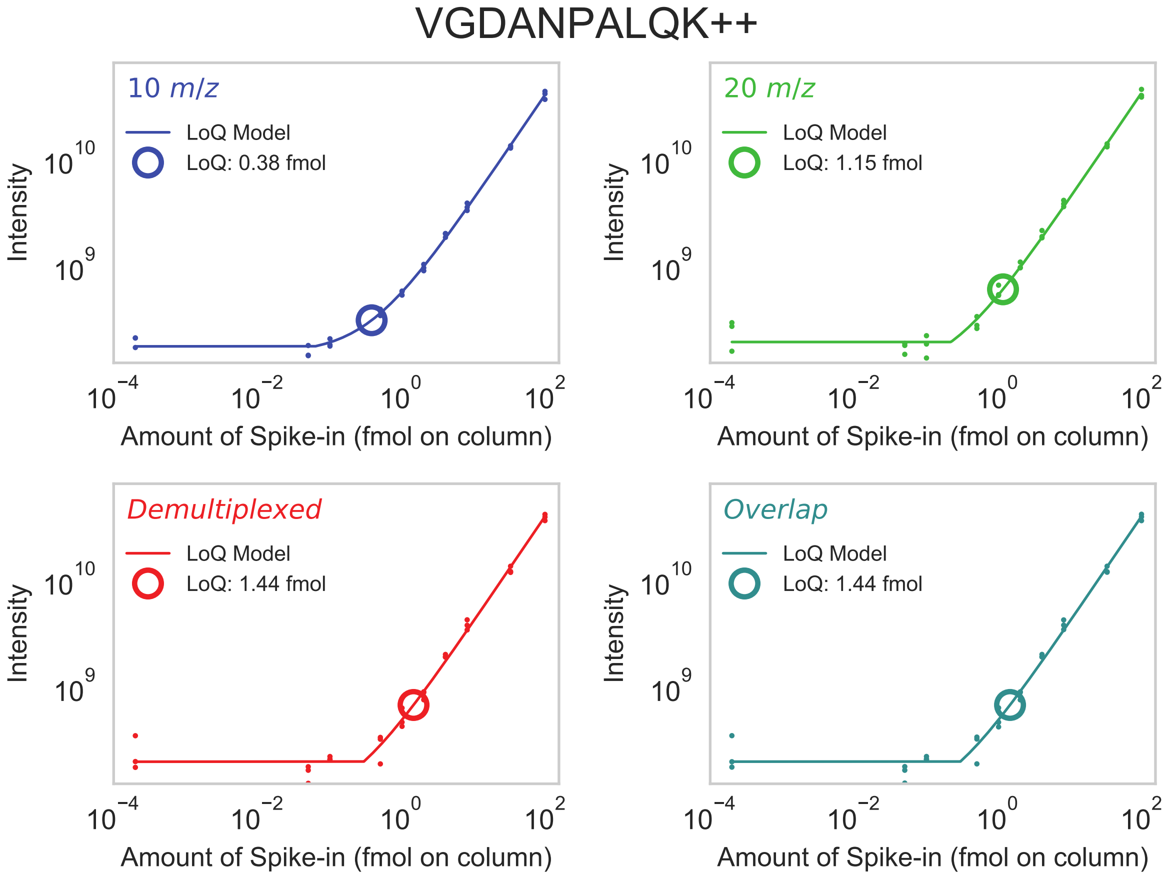
VGDANPALQK++
10 m/z
LoQ Model
LoQ: 0.38 fmol
20 m/z
LoQ Model
LoQ: 1.15 fmol
Demultiplexed
LoQ Model
LoQ: 1.44 fmol
Overlap
LoQ Model
LoQ: 1.44 fmol
Intensity
Amount of Spike-in (fmol on column)
10^{9}
10^{10}
10^{-4}
10^{-2}
10^{0}
10^{2}

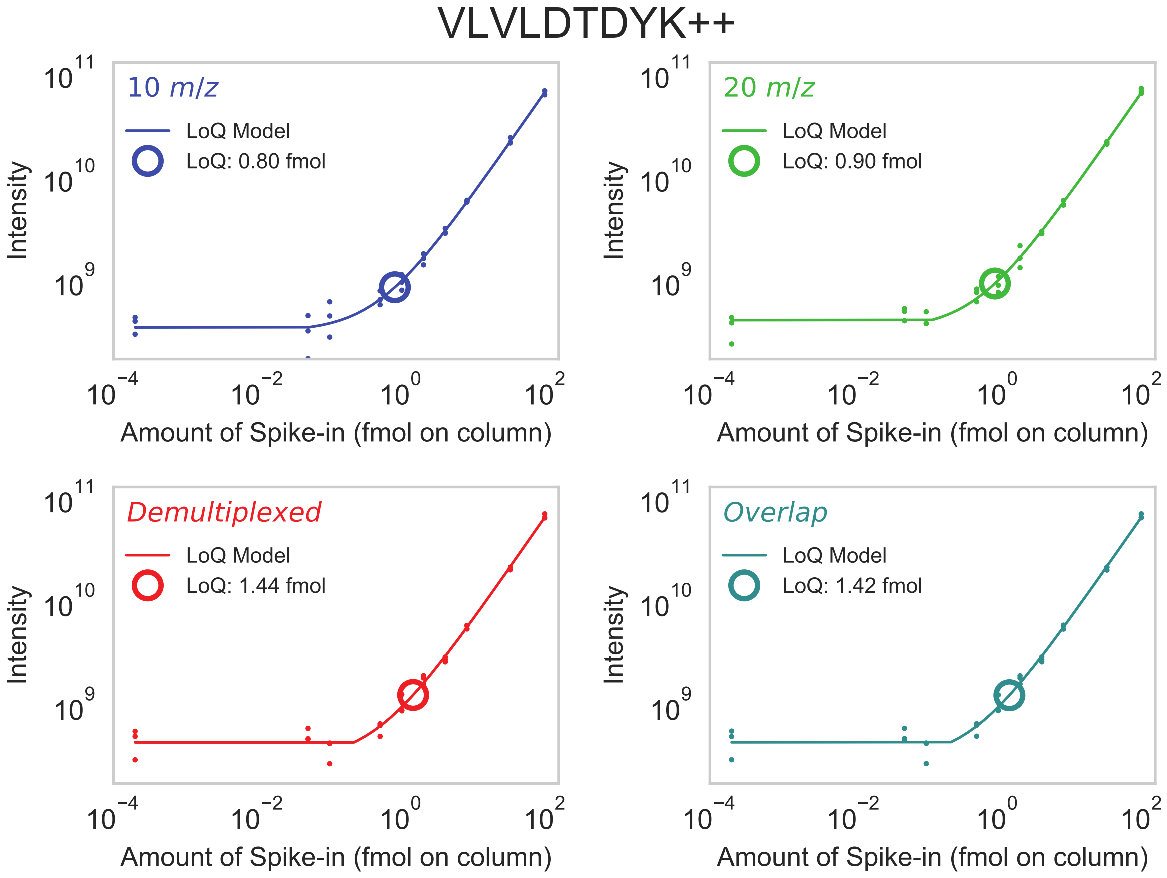
VLVLDTDYK++
10 m/z
LoQ Model
LoQ: 0.80 fmol
20 m/z
LoQ Model
LoQ: 0.90 fmol
Demultiplexed
LoQ Model
LoQ: 1.44 fmol
Overlap
LoQ Model
LoQ: 1.42 fmol
Intensity
Amount of Spike-in (fmol on column)

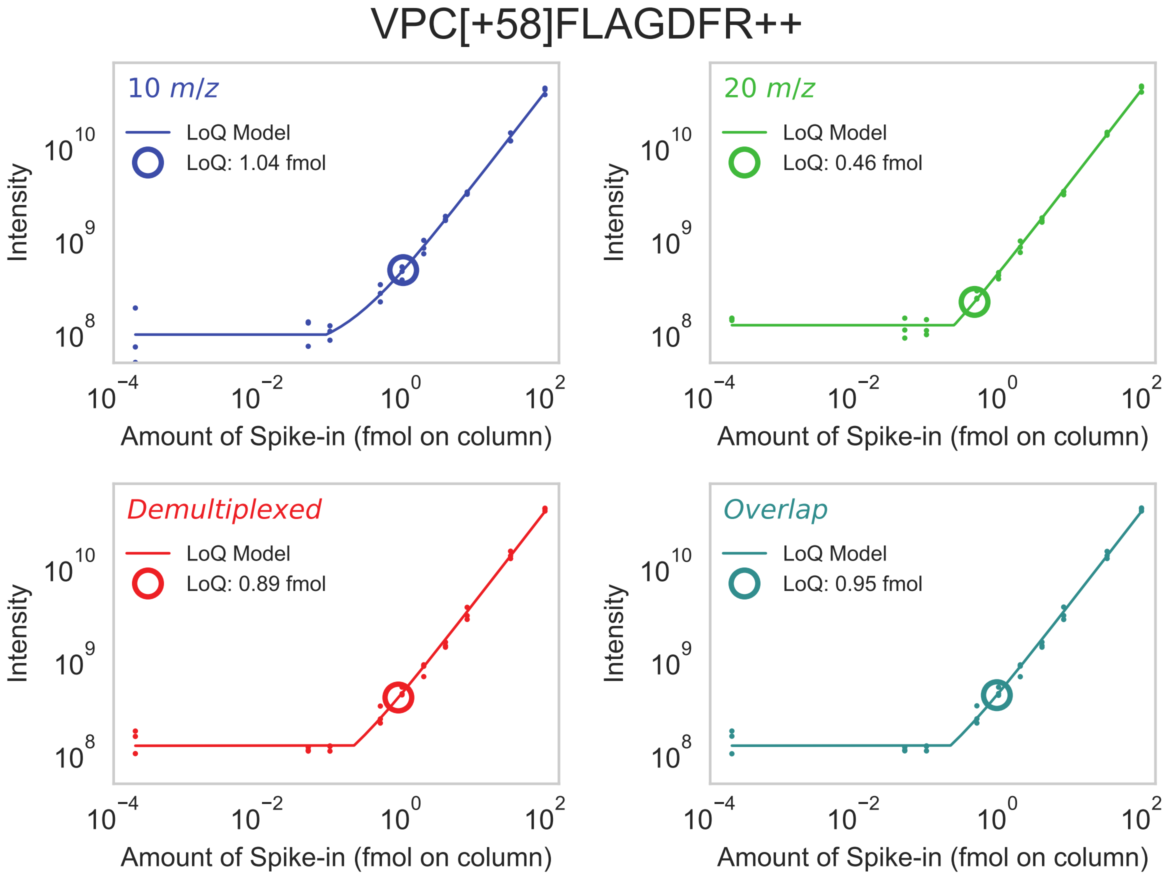

VPC[+58]FLAGDFR++
10 m/z
LoQ Model
LoQ: 1.04 fmol
20 m/z
LoQ Model
LoQ: 0.46 fmol
Demultiplexed
LoQ Model
LoQ: 0.89 fmol
Overlap
LoQ Model
LoQ: 0.95 fmol
Intensity
Amount of Spike-in (fmol on column)

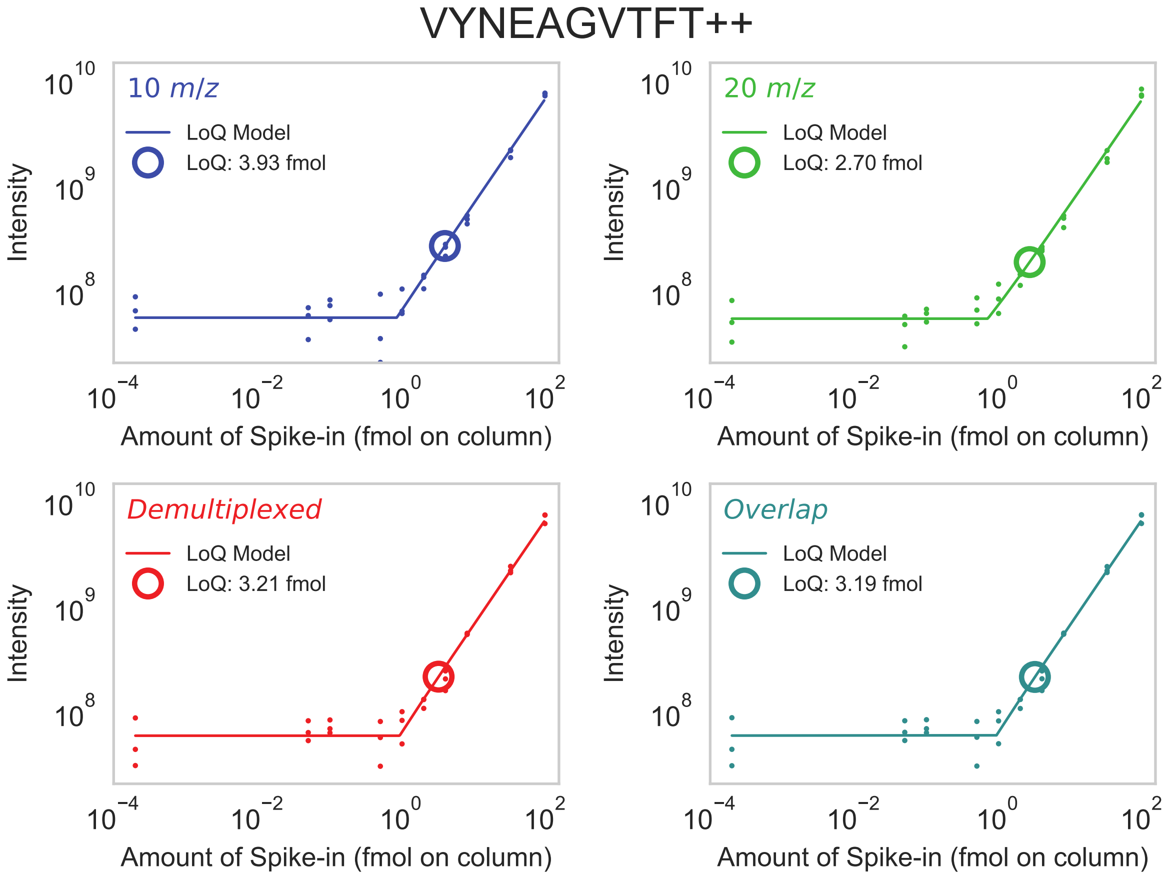
VYNEAGVTFT++
10 m/z
LoQ Model
LoQ: 3.93 fmol
20 m/z
LoQ Model
LoQ: 2.70 fmol
Demultiplexed
LoQ Model
LoQ: 3.21 fmol
Overlap
LoQ Model
LoQ: 3.19 fmol
Intensity
Amount of Spike-in (fmol on column)
10^8
10^9
10^10
10^-4
10^-2
10^0
10^2

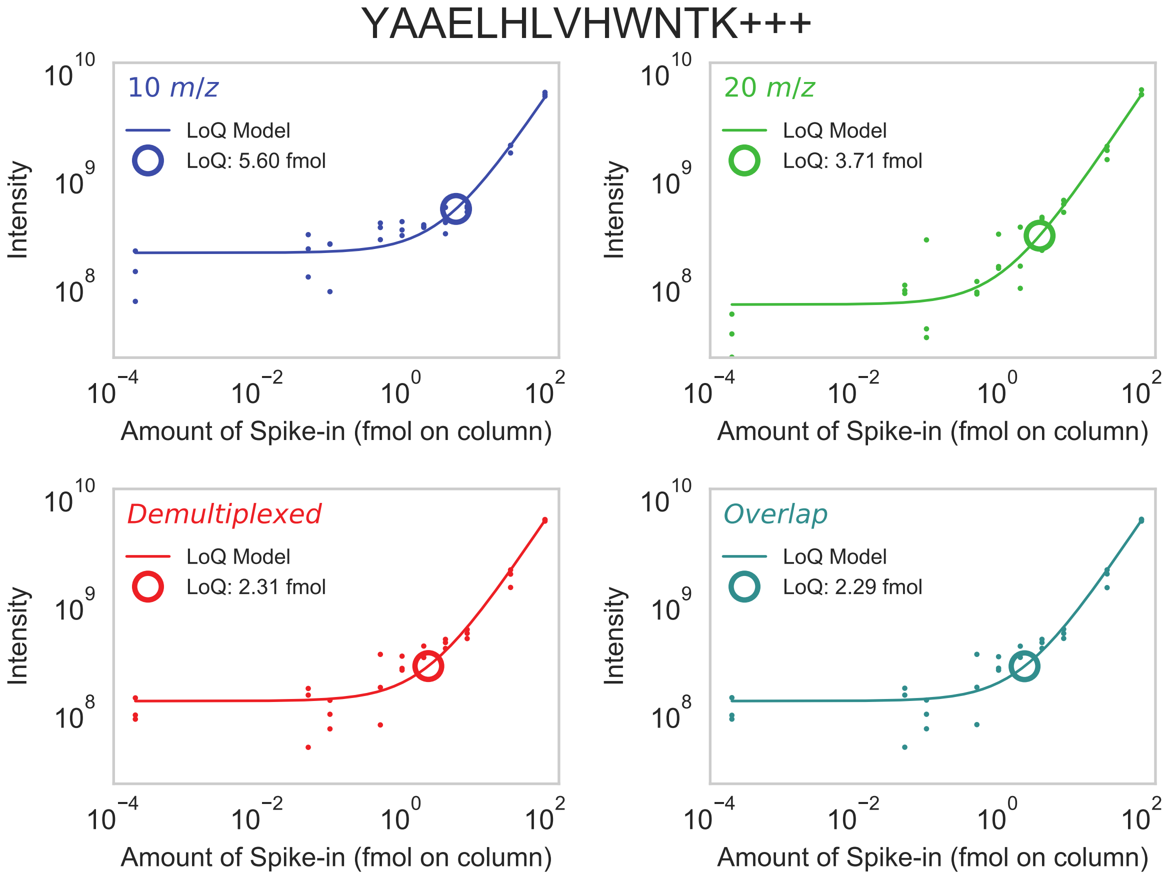

YAAELHLVHWNTK+++
10 m/z
LoQ Model
LoQ: 5.60 fmol
20 m/z
LoQ Model
LoQ: 3.71 fmol
Demultiplexed
LoQ Model
LoQ: 2.31 fmol
Overlap
LoQ Model
LoQ: 2.29 fmol
Intensity
Amount of Spike-in (fmol on column)
10^{10}
10^{9}
10^{8}
10^{-4}
10^{-2}
10^{0}
10^{2}

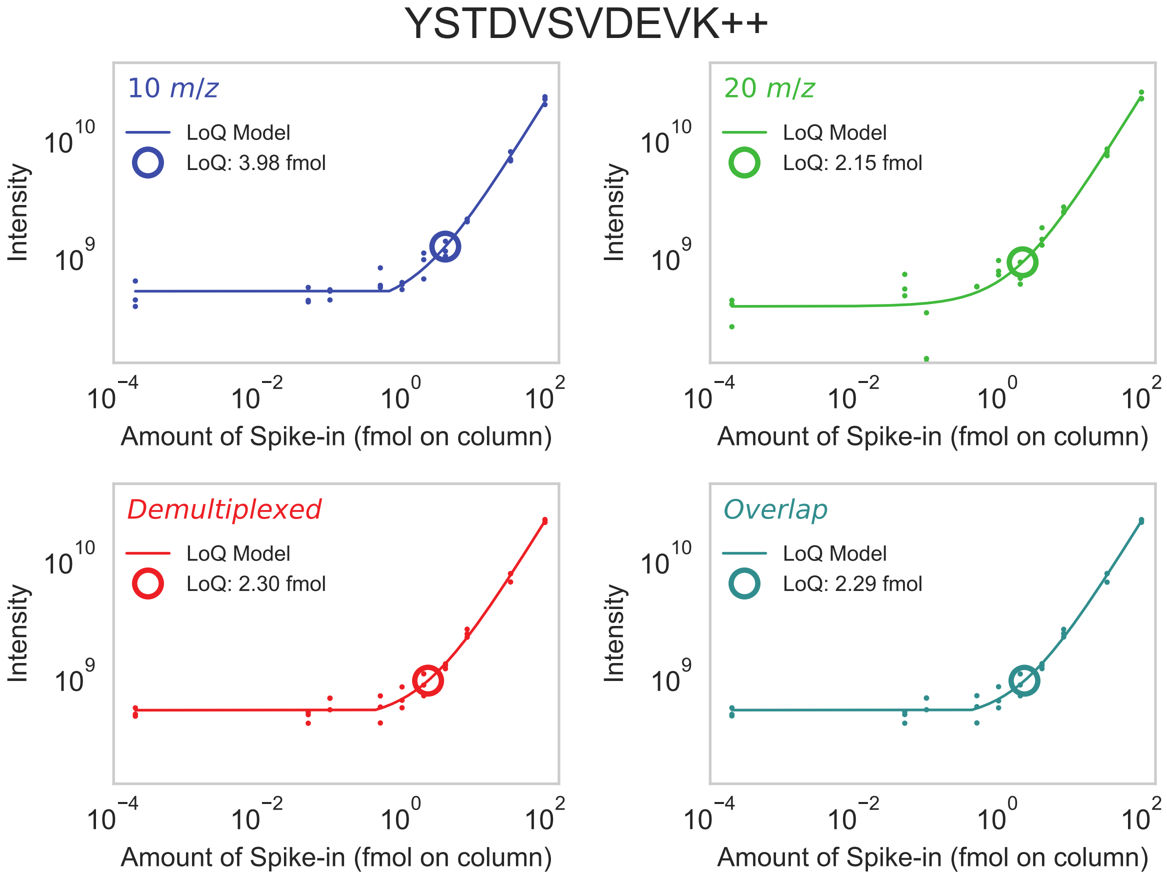

YSTDVSVDEVK++
10 m/z
LoQ Model
LoQ: 3.98 fmol
20 m/z
LoQ Model
LoQ: 2.15 fmol
Demultiplexed
LoQ Model
LoQ: 2.30 fmol
Overlap
LoQ Model
LoQ: 2.29 fmol
Intensity
Amount of Spike-in (fmol on column)
