# Supplementary material for: Improving Precursor Selectivity in Data-Independent Acquisition Using Overlapping Windows
Source: J Am Soc Mass Spectrom. 2019 Jan 22;30(4):669–84. doi: 10.1007/s13361-018-2122-8 (PMC6445824; doi:10.1007/s13361-018-2122-8)
Supplement: Supplementary file 11 — (PDF 1349 kb) [file 13361_2018_2122_MOESM11_ESM.pdf]

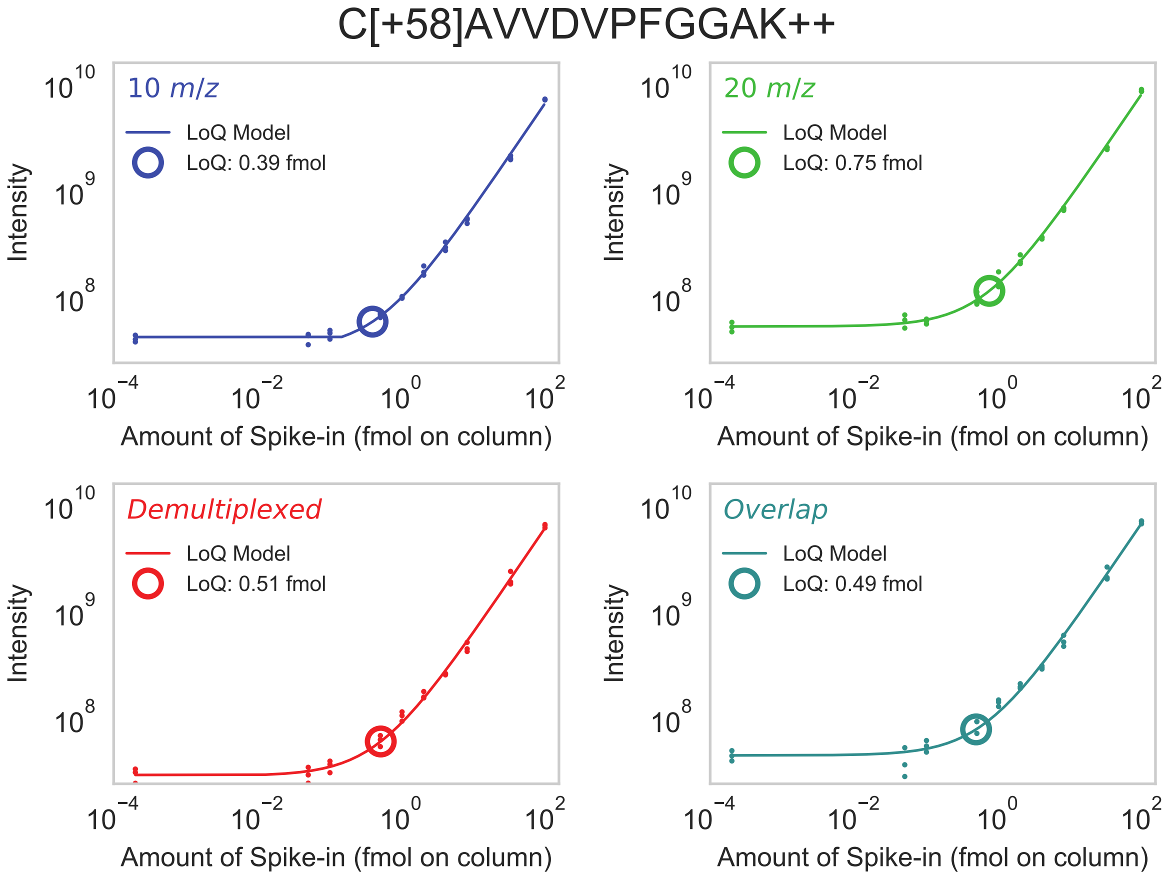

C[+58]AVVDVPFGGAK++
10 m/z
LoQ Model
LoQ: 0.39 fmol
20 m/z
LoQ Model
LoQ: 0.75 fmol
Demultiplexed
LoQ Model
LoQ: 0.51 fmol
Overlap
LoQ Model
LoQ: 0.49 fmol
Intensity
Amount of Spike-in (fmol on column)

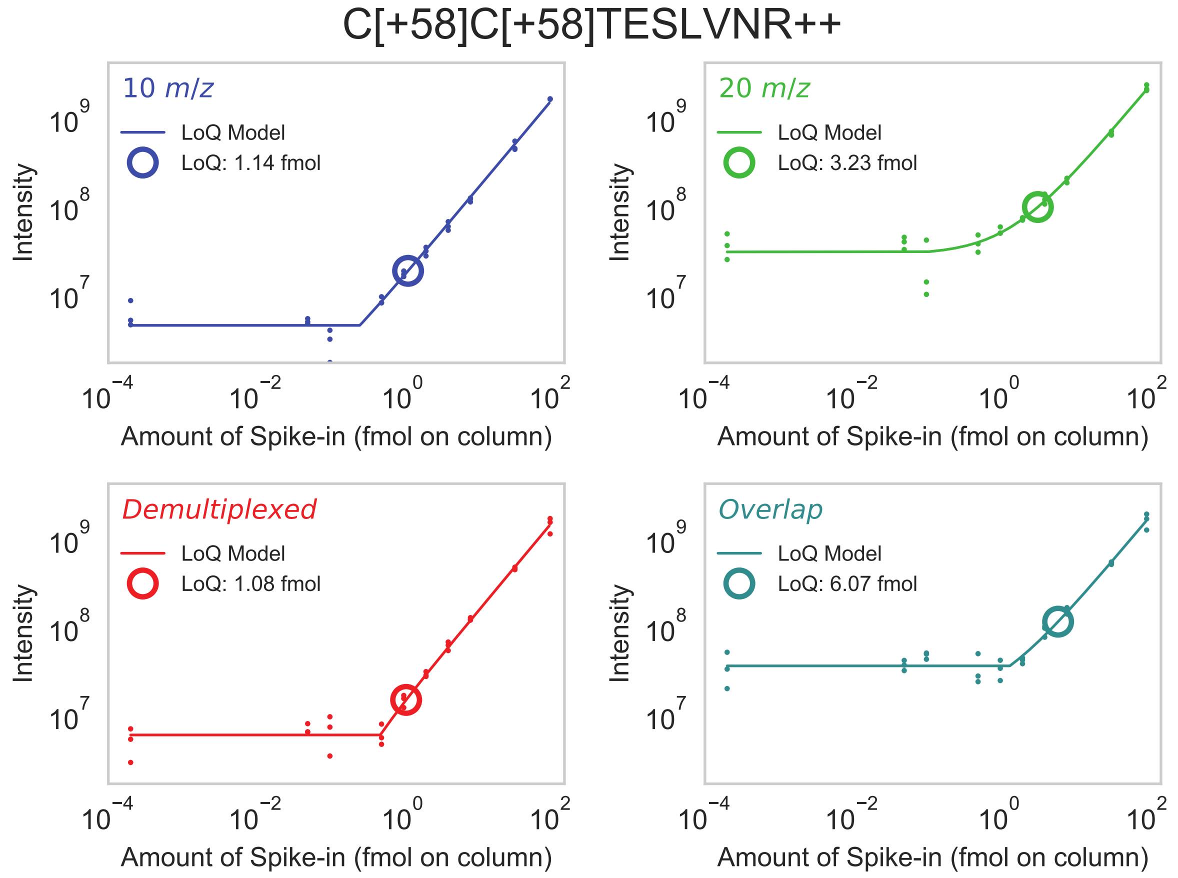
C[+58]C[+58]TESLVNR++
10 m/z
LoQ Model
LoQ: 1.14 fmol
20 m/z
LoQ Model
LoQ: 3.23 fmol
Demultiplexed
LoQ Model
LoQ: 1.08 fmol
Overlap
LoQ Model
LoQ: 6.07 fmol
Intensity
Amount of Spike-in (fmol on column)

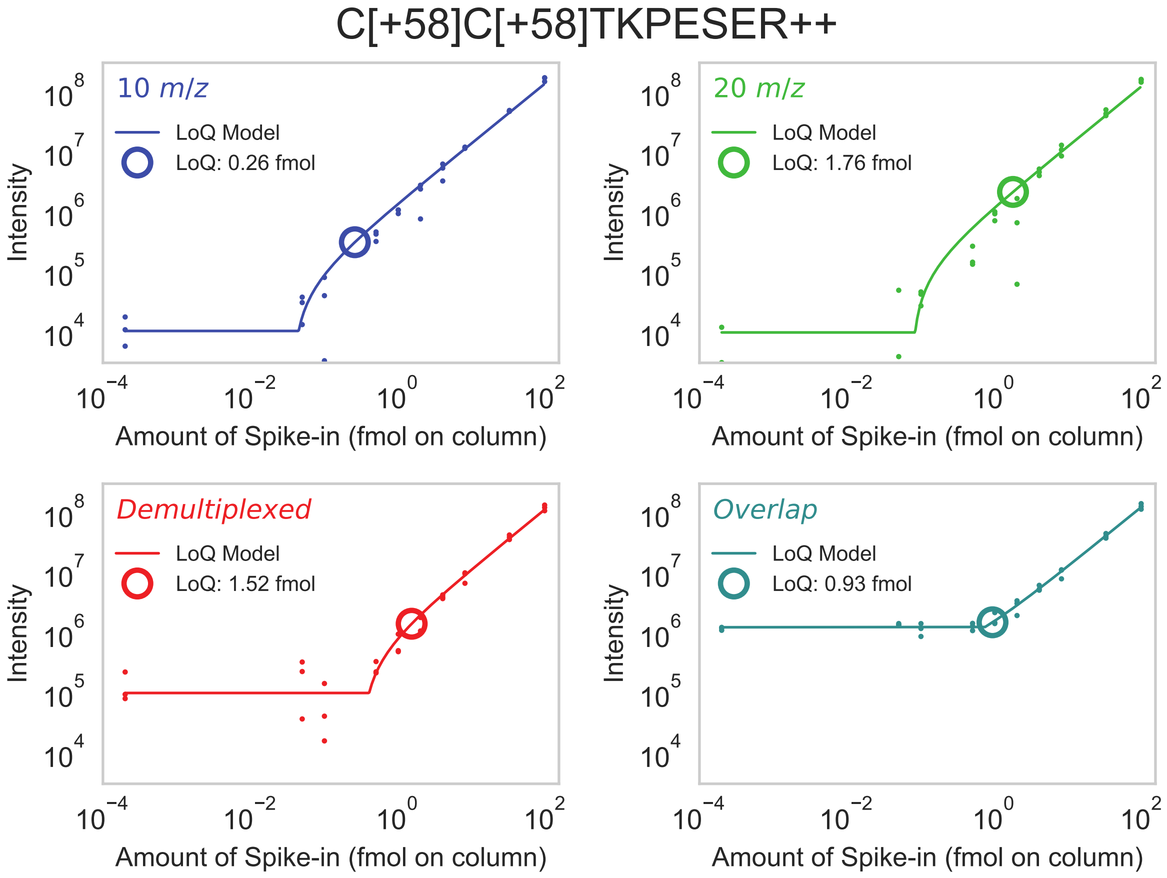
C[+58]C[+58]TKPESER++
10 m/z
LoQ Model
LoQ: 0.26 fmol
20 m/z
LoQ Model
LoQ: 1.76 fmol
Demultiplexed
LoQ Model
LoQ: 1.52 fmol
Overlap
LoQ Model
LoQ: 0.93 fmol
Intensity
Amount of Spike-in (fmol on column)

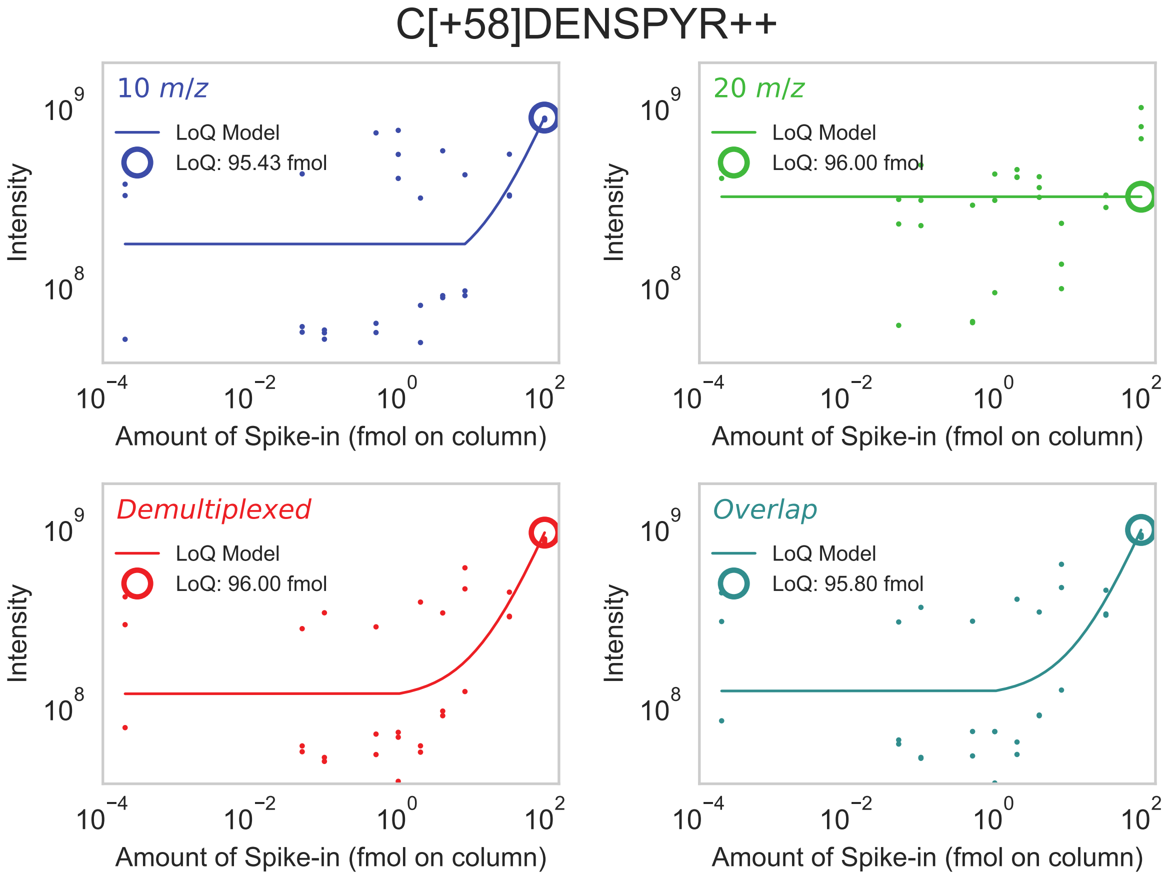

C[+58]DENSPYR++
10 m/z
LoQ Model
LoQ: 95.43 fmol
20 m/z
LoQ Model
LoQ: 96.00 fmol
Demultiplexed
LoQ Model
LoQ: 96.00 fmol
Overlap
LoQ Model
LoQ: 95.80 fmol
Intensity
Amount of Spike-in (fmol on column)

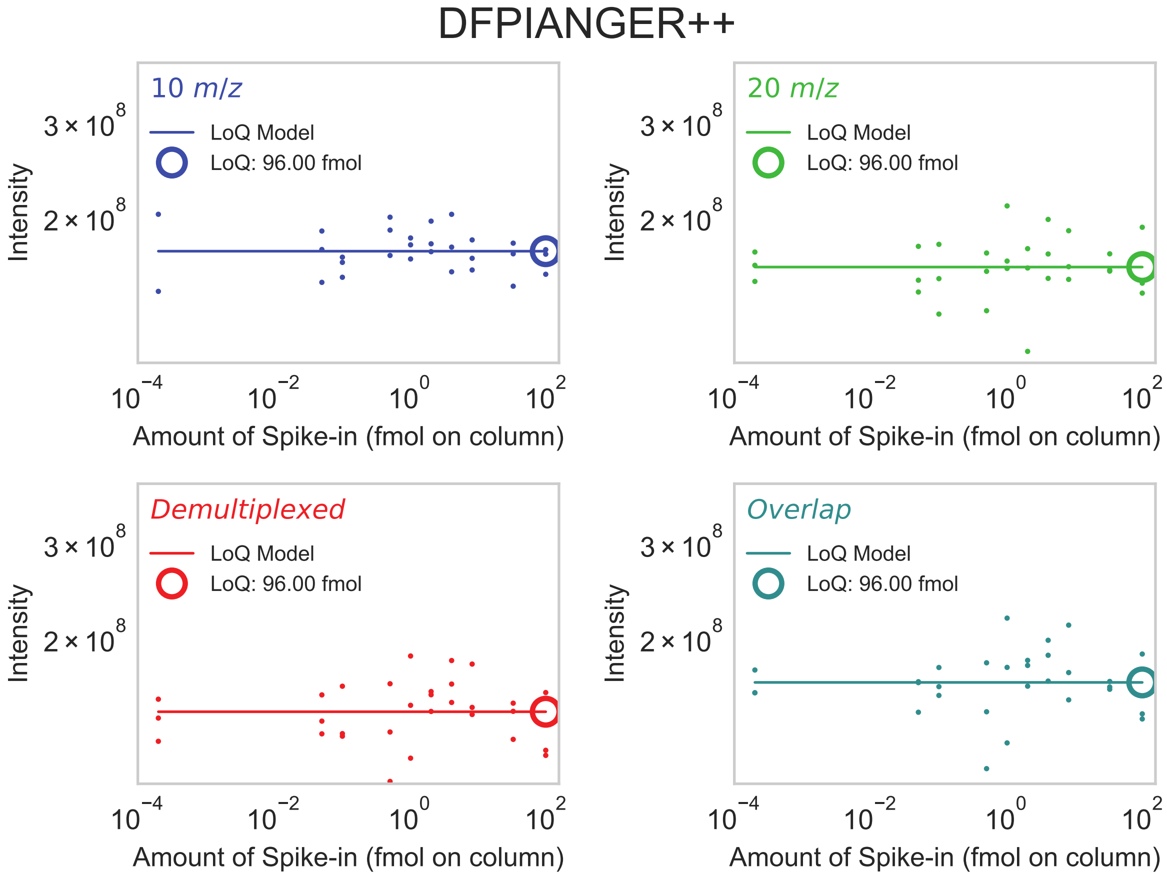
DFPIANGER++
10 m/z
LoQ Model
LoQ: 96.00 fmol
20 m/z
LoQ Model
LoQ: 96.00 fmol
Demultiplexed
LoQ Model
LoQ: 96.00 fmol
Overlap
LoQ Model
LoQ: 96.00 fmol
Intensity
Amount of Spike-in (fmol on column)

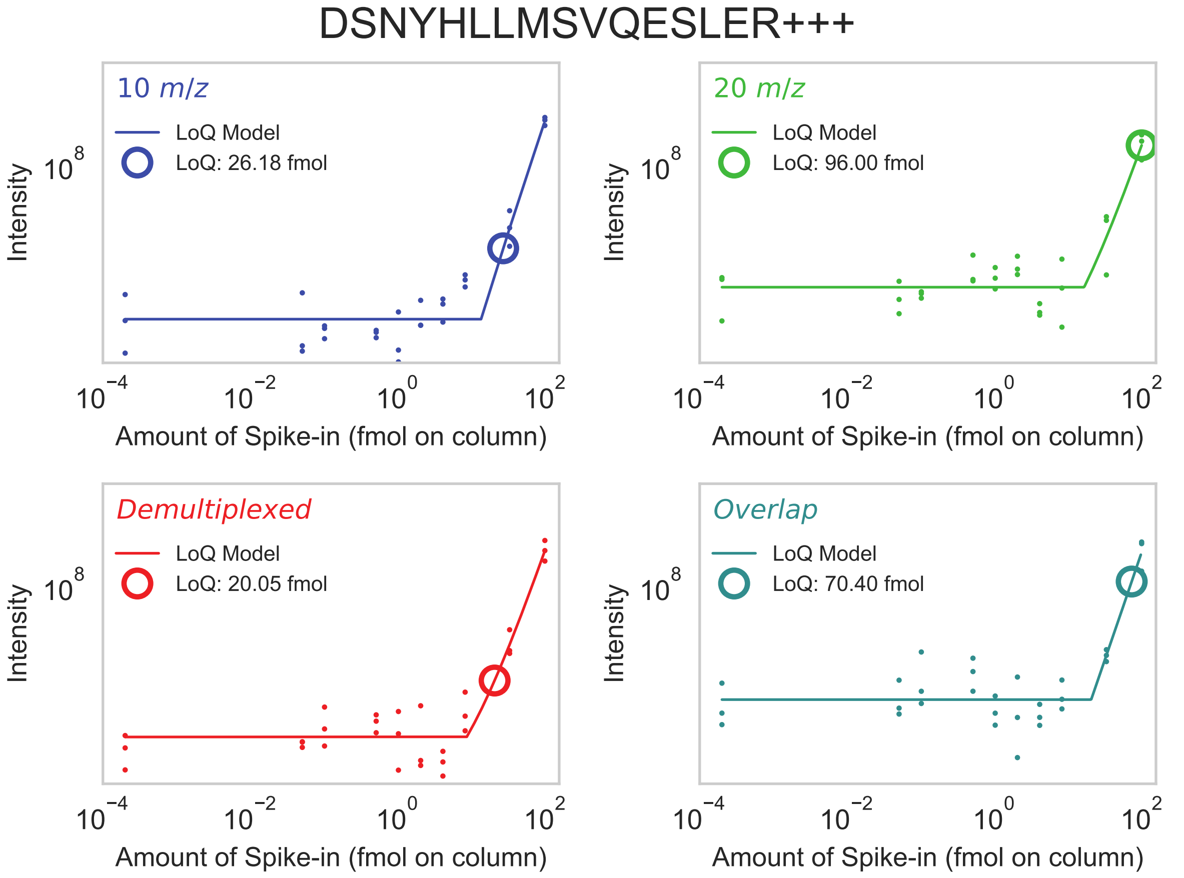
DSNYHLLMSVQESLER+++
10 m/z
LoQ Model
LoQ: 26.18 fmol
20 m/z
LoQ Model
LoQ: 96.00 fmol
Demultiplexed
LoQ Model
LoQ: 20.05 fmol
Overlap
LoQ Model
LoQ: 70.40 fmol
Intensity
10^8
10^-4
10^-2
10^0
10^2
Amount of Spike-in (fmol on column)

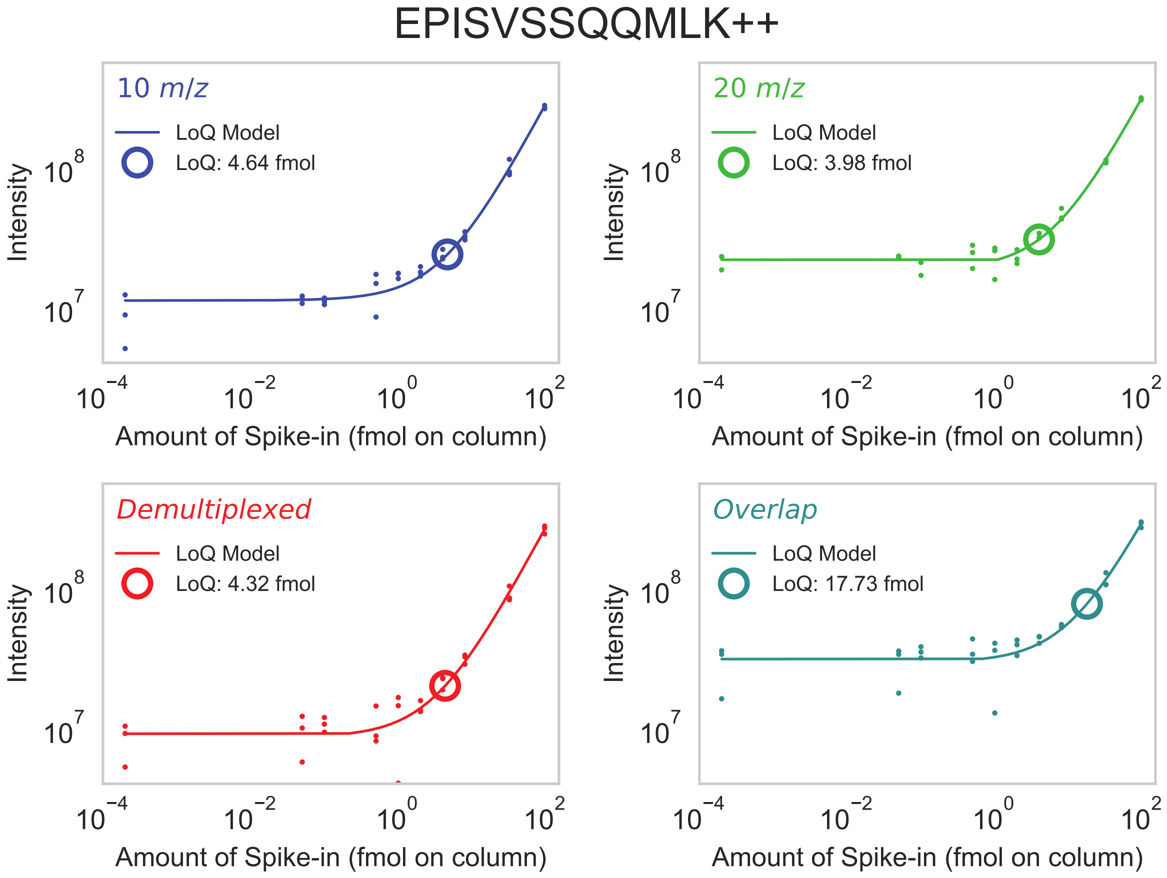
EPISVSSQQMLK++
10 m/z
LoQ Model
LoQ: 4.64 fmol
20 m/z
LoQ Model
LoQ: 3.98 fmol
Demultiplexed
LoQ Model
LoQ: 4.32 fmol
Overlap
LoQ Model
LoQ: 17.73 fmol
Intensity
Amount of Spike-in (fmol on column)

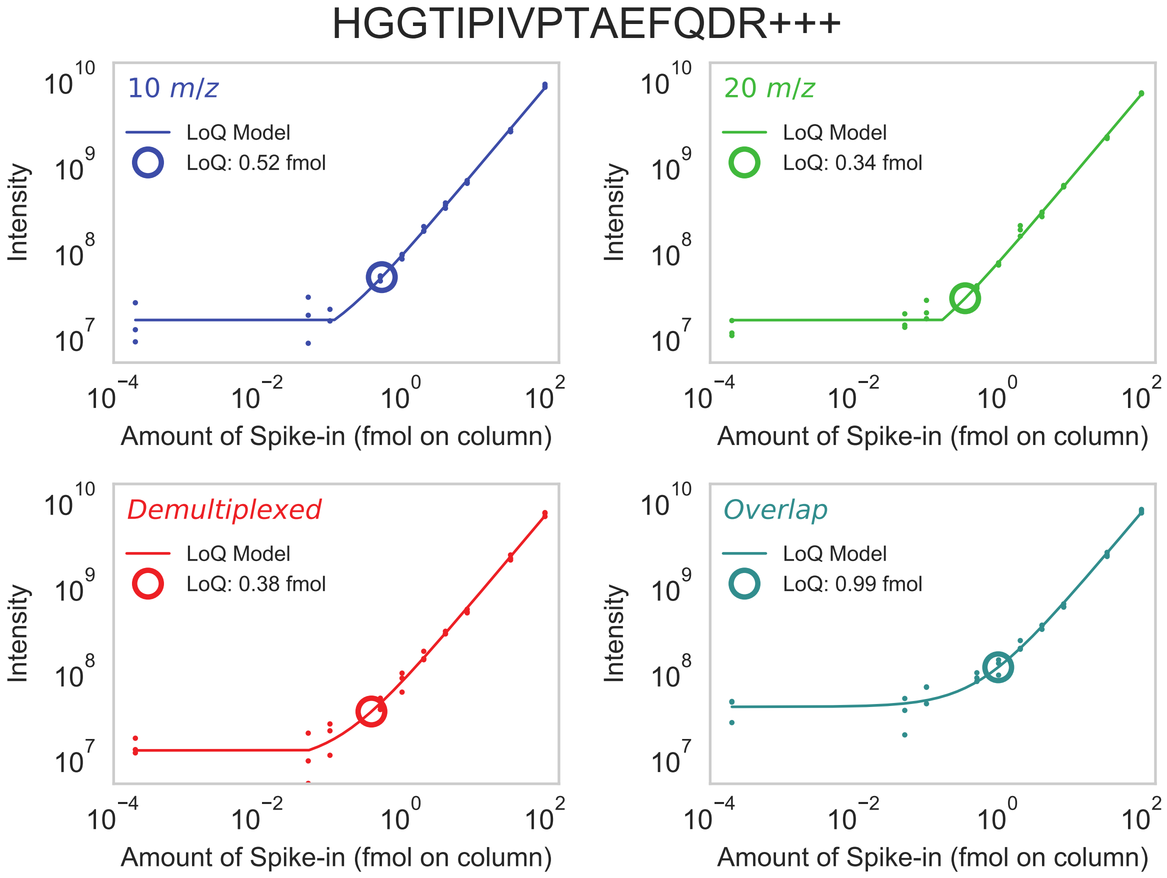

HGGTIPIVPTAEFQDR+++
10 m/z
LoQ Model
LoQ: 0.52 fmol
20 m/z
LoQ Model
LoQ: 0.34 fmol
Demultiplexed
LoQ Model
LoQ: 0.38 fmol
Overlap
LoQ Model
LoQ: 0.99 fmol
Intensity
Amount of Spike-in (fmol on column)

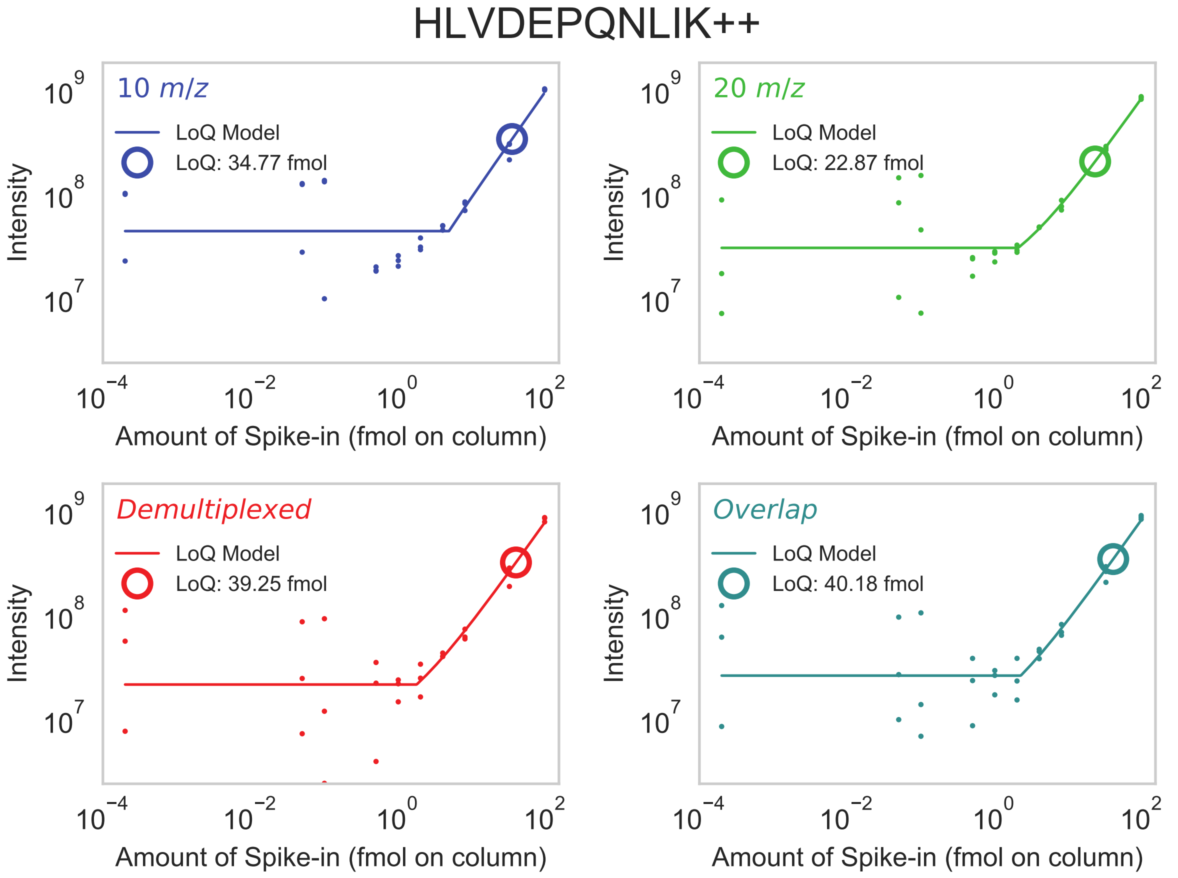

HLVDEPQNLIK++
10 m/z
LoQ Model
LoQ: 34.77 fmol
20 m/z
LoQ Model
LoQ: 22.87 fmol
Demultiplexed
LoQ Model
LoQ: 39.25 fmol
Overlap
LoQ Model
LoQ: 40.18 fmol
Intensity
Amount of Spike-in (fmol on column)

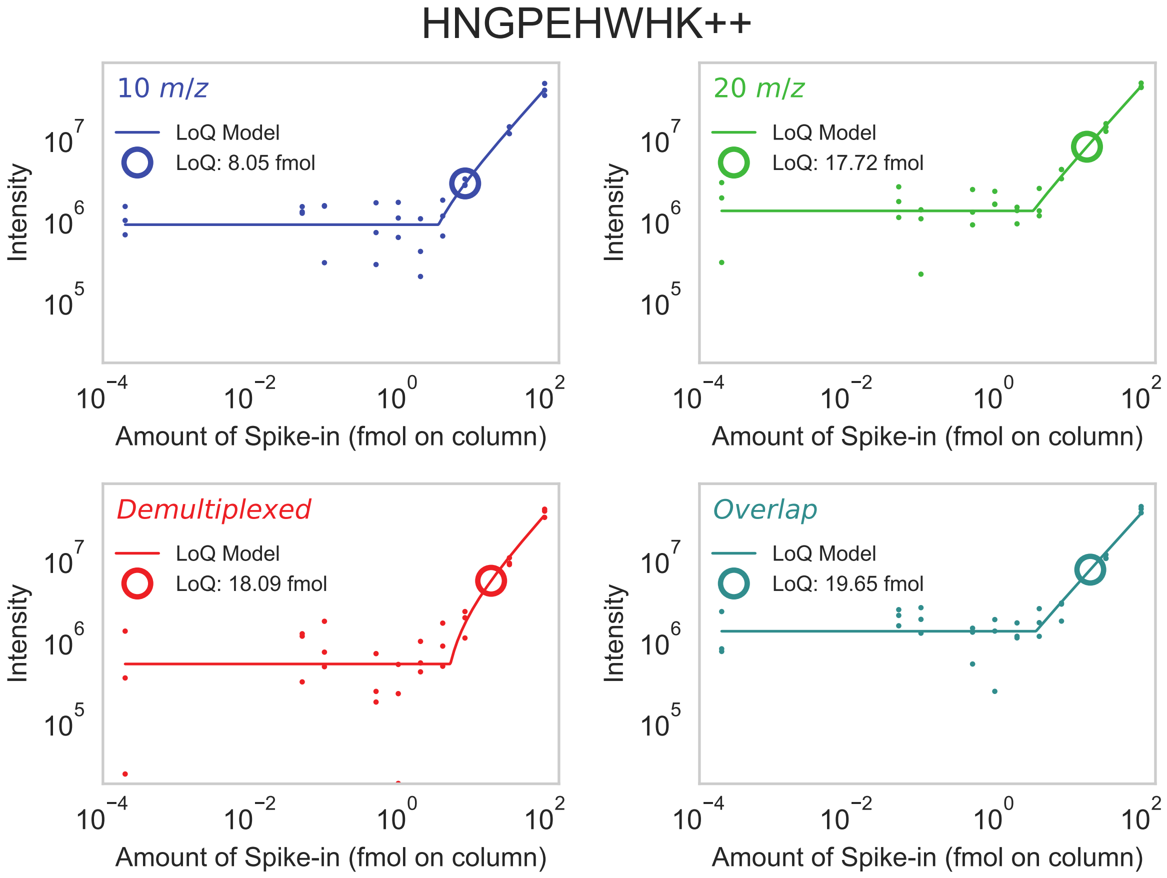
HNGPEHWHK++
10 m/z
LoQ Model
LoQ: 8.05 fmol
20 m/z
LoQ Model
LoQ: 17.72 fmol
Demultiplexed
LoQ Model
LoQ: 18.09 fmol
Overlap
LoQ Model
LoQ: 19.65 fmol
Intensity
Amount of Spike-in (fmol on column)

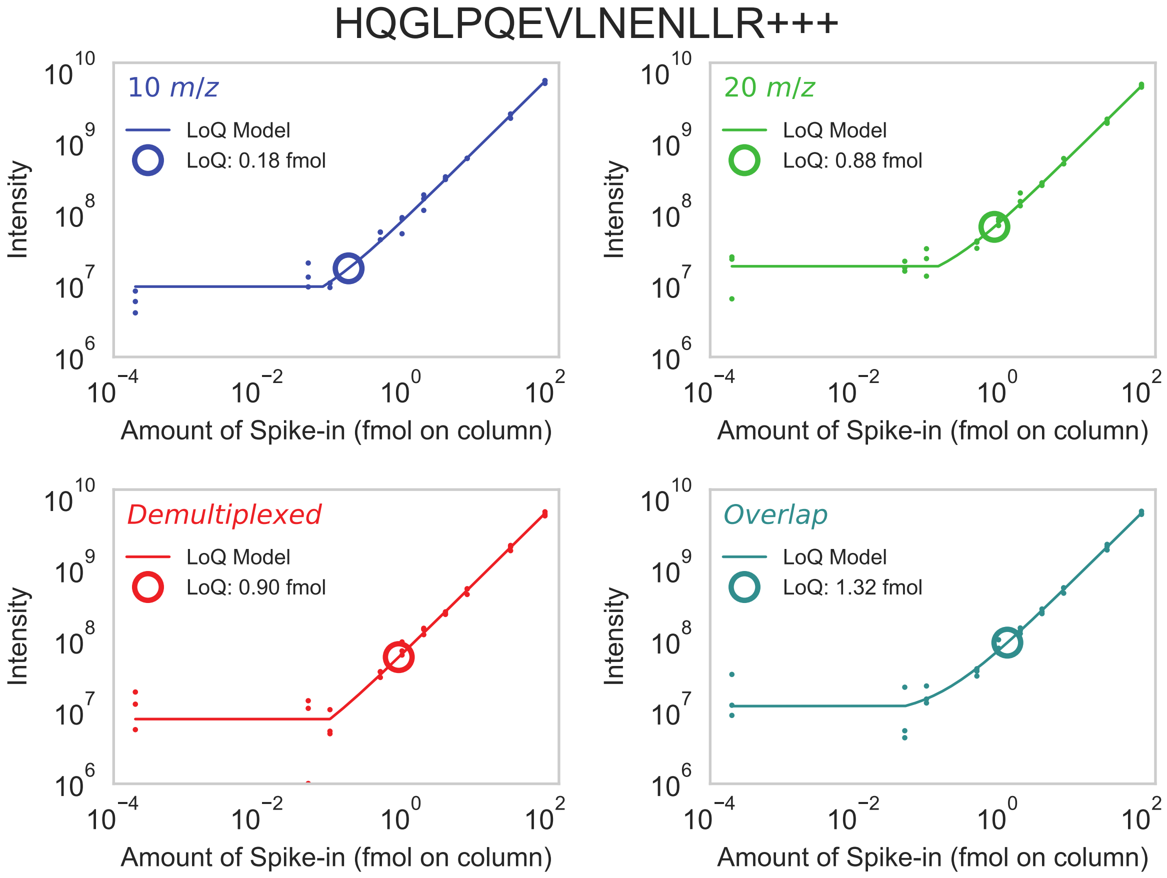

HQGLPQEVLNENLLR+++
10 m/z
LoQ Model
LoQ: 0.18 fmol
20 m/z
LoQ Model
LoQ: 0.88 fmol
Demultiplexed
LoQ Model
LoQ: 0.90 fmol
Overlap
LoQ Model
LoQ: 1.32 fmol
Intensity
Amount of Spike-in (fmol on column)

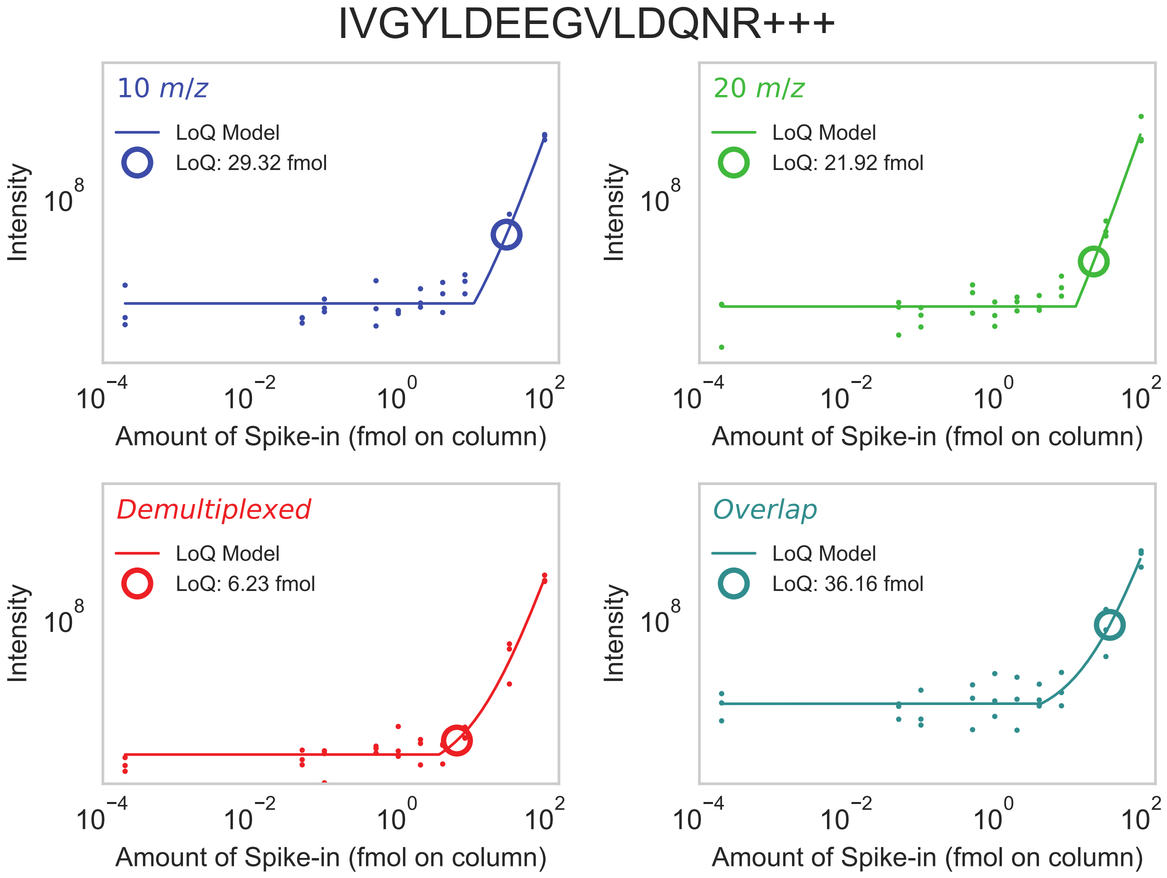
IVGYLDEEGVLDQNR+++
10 m/z
LoQ Model
LoQ: 29.32 fmol
20 m/z
LoQ Model
LoQ: 21.92 fmol
Demultiplexed
LoQ Model
LoQ: 6.23 fmol
Overlap
LoQ Model
LoQ: 36.16 fmol
Intensity
10^8
10^-4
10^-2
10^0
10^2
Amount of Spike-in (fmol on column)

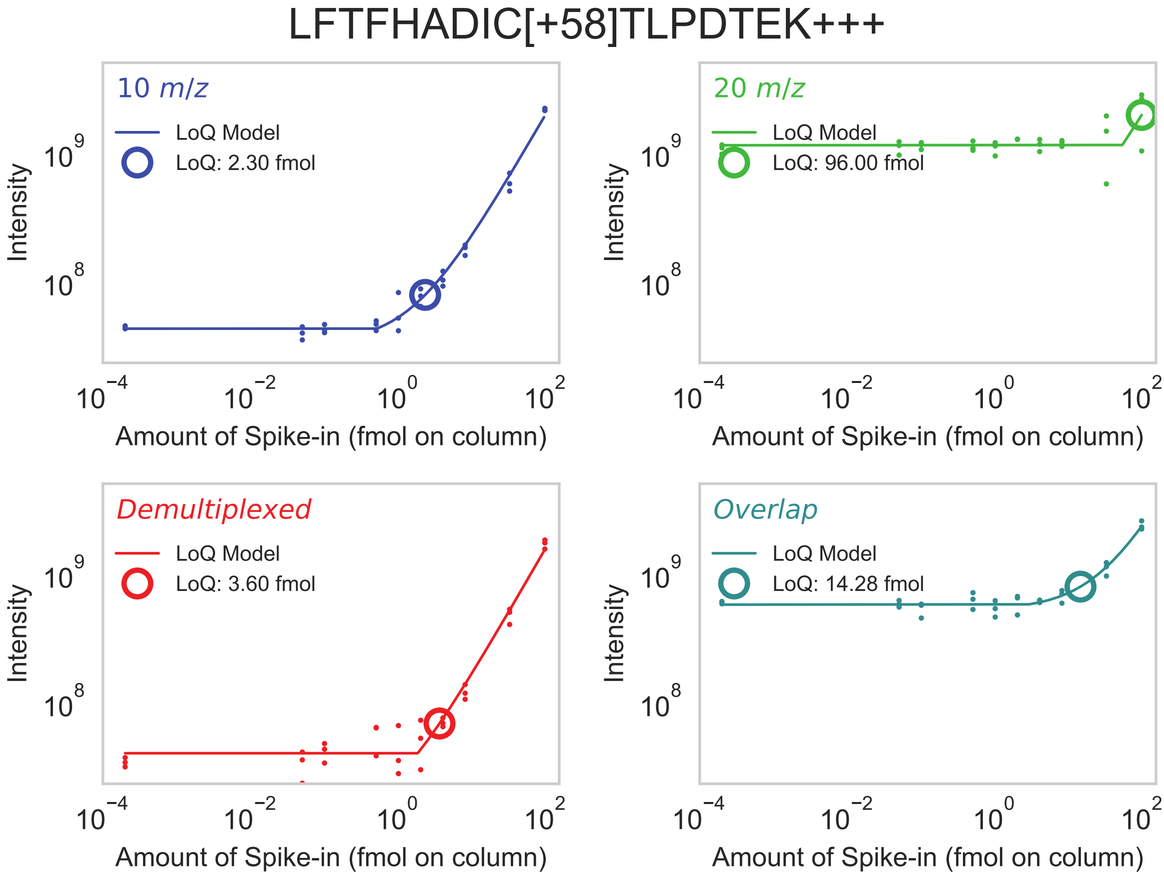

LFTFHADIC[+58]TLPDTEK+++
10 m/z
LoQ Model
LoQ: 2.30 fmol
20 m/z
LoQ Model
LoQ: 96.00 fmol
Demultiplexed
LoQ Model
LoQ: 3.60 fmol
Overlap
LoQ Model
LoQ: 14.28 fmol
Intensity
Amount of Spike-in (fmol on column)

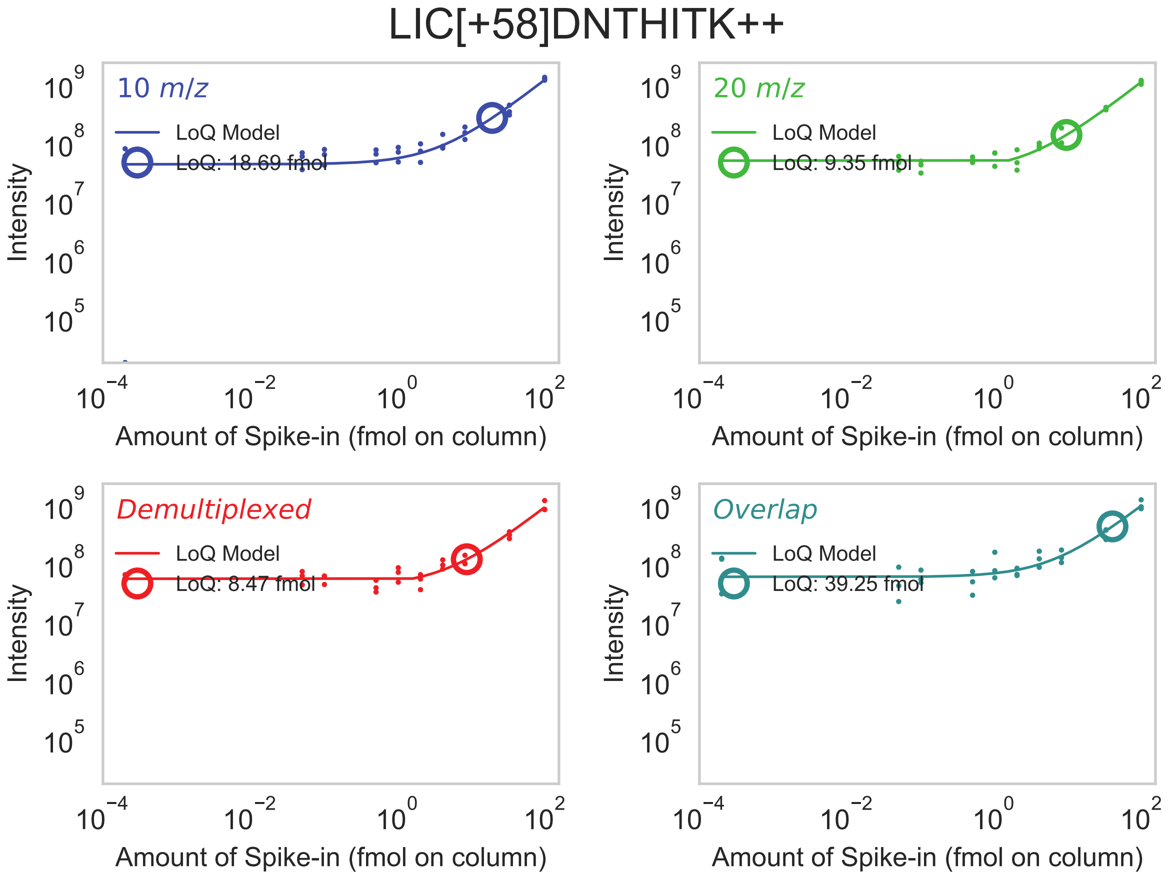
LIC[+58]DNTHITK++
10 m/z
LoQ Model
LoQ: 18.69 fmol
20 m/z
LoQ Model
LoQ: 9.35 fmol
Demultiplexed
LoQ Model
LoQ: 8.47 fmol
Overlap
LoQ Model
LoQ: 39.25 fmol
Intensity
Amount of Spike-in (fmol on column)

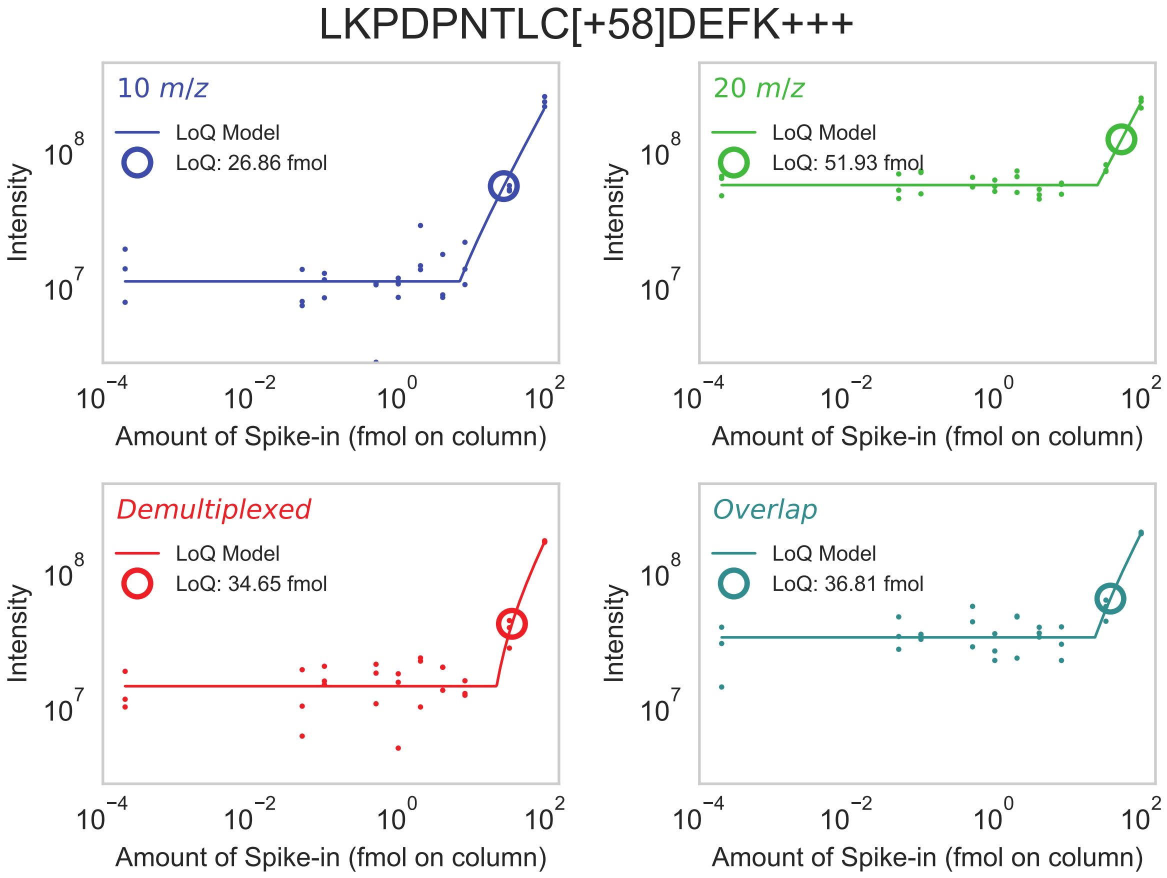
LKPDPNTLC[+58]DEFK+++
10 m/z
LoQ Model
LoQ: 26.86 fmol
20 m/z
LoQ Model
LoQ: 51.93 fmol
Demultiplexed
LoQ Model
LoQ: 34.65 fmol
Overlap
LoQ Model
LoQ: 36.81 fmol
Intensity
Amount of Spike-in (fmol on column)

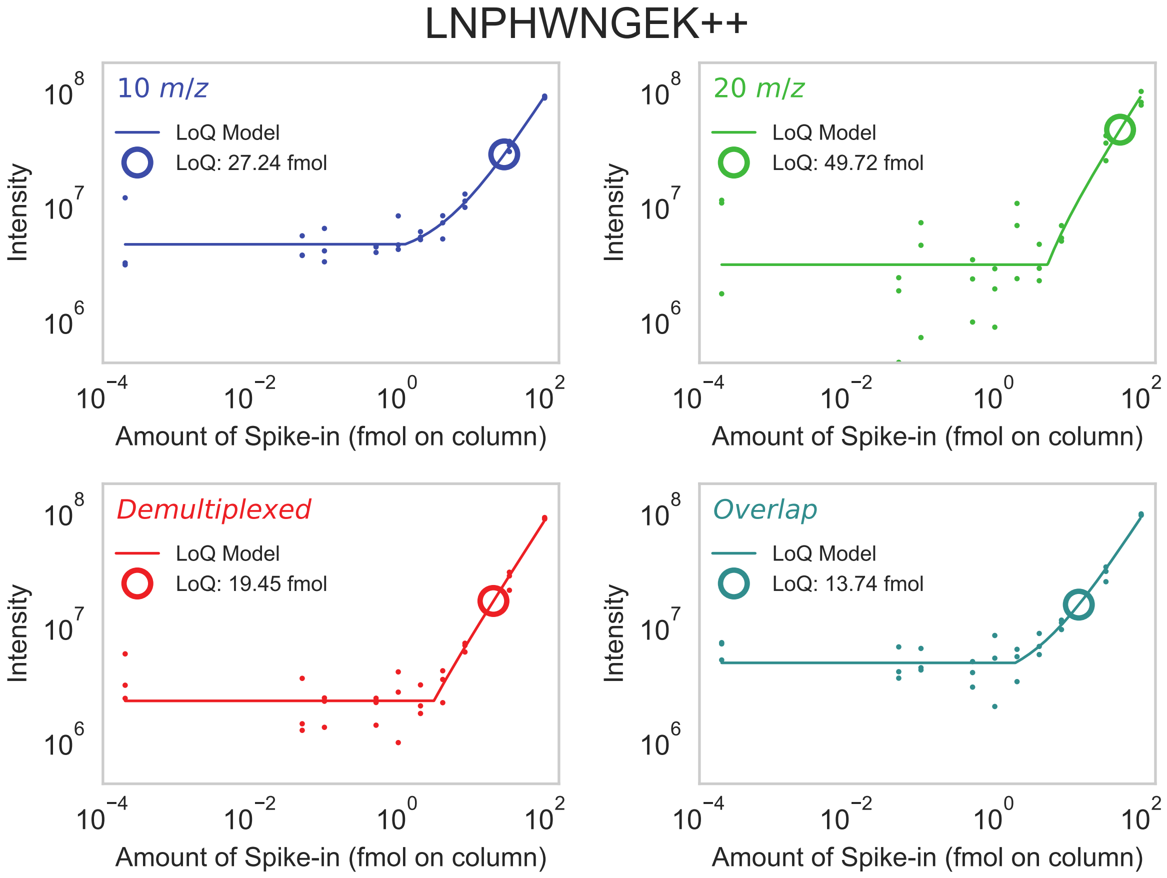

LNPHWNGEK++
10 m/z
LoQ Model
LoQ: 27.24 fmol
20 m/z
LoQ Model
LoQ: 49.72 fmol
Demultiplexed
LoQ Model
LoQ: 19.45 fmol
Overlap
LoQ Model
LoQ: 13.74 fmol
Intensity
Amount of Spike-in (fmol on column)

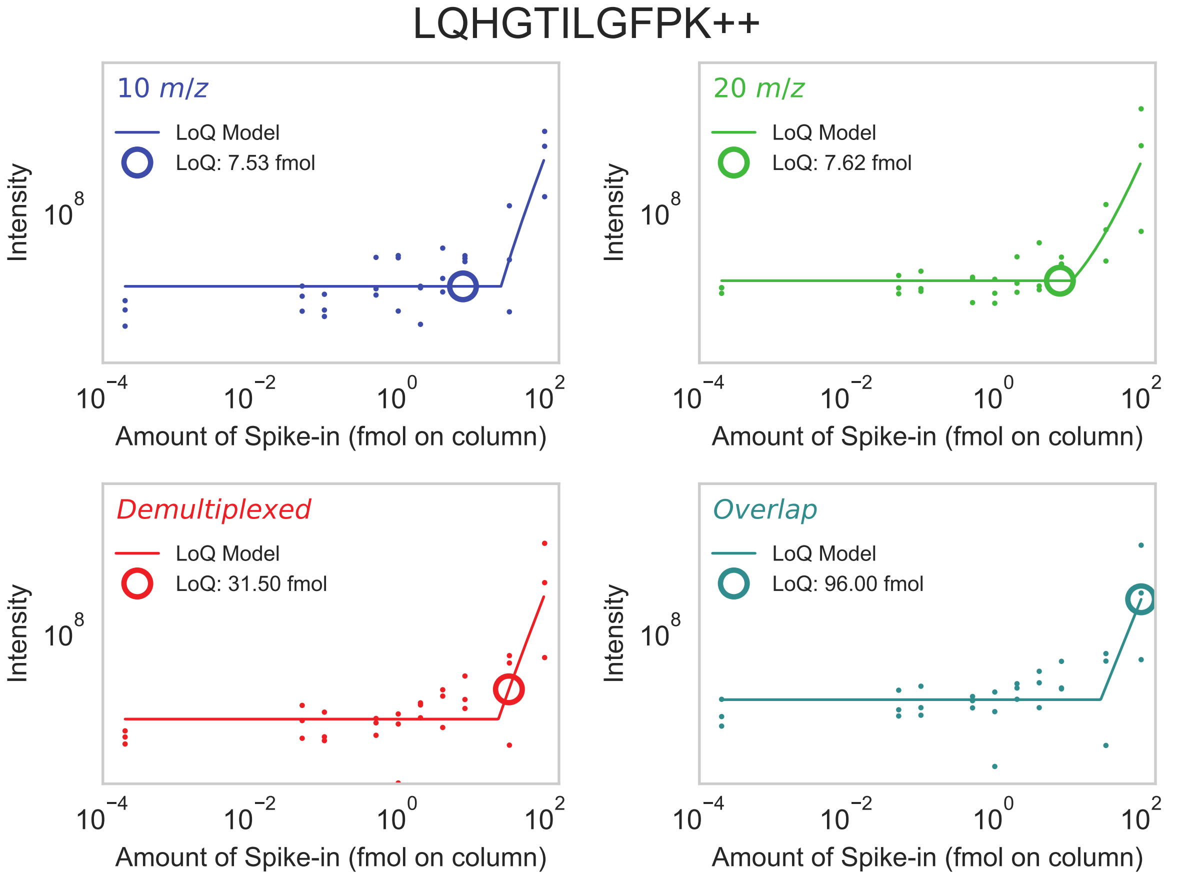
LQHGTILGFPK++
10 m/z
LoQ Model
LoQ: 7.53 fmol
20 m/z
LoQ Model
LoQ: 7.62 fmol
Demultiplexed
LoQ Model
LoQ: 31.50 fmol
Overlap
LoQ Model
LoQ: 96.00 fmol
Intensity
10^8
10^-4
10^-2
10^0
10^2
Amount of Spike-in (fmol on column)

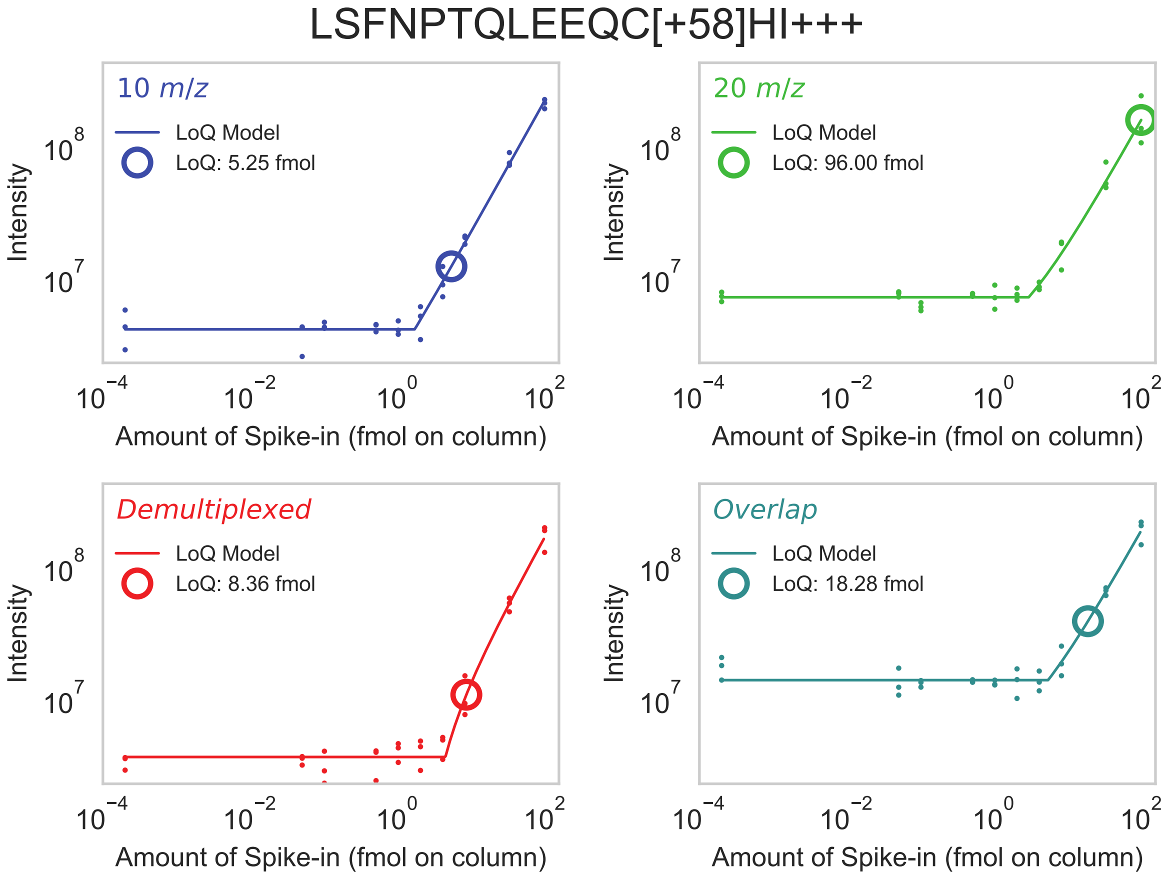

LSFNPTQLEEQC[+58]HI+++
10 m/z
LoQ Model
LoQ: 5.25 fmol
20 m/z
LoQ Model
LoQ: 96.00 fmol
Demultiplexed
LoQ Model
LoQ: 8.36 fmol
Overlap
LoQ Model
LoQ: 18.28 fmol
Intensity
Amount of Spike-in (fmol on column)

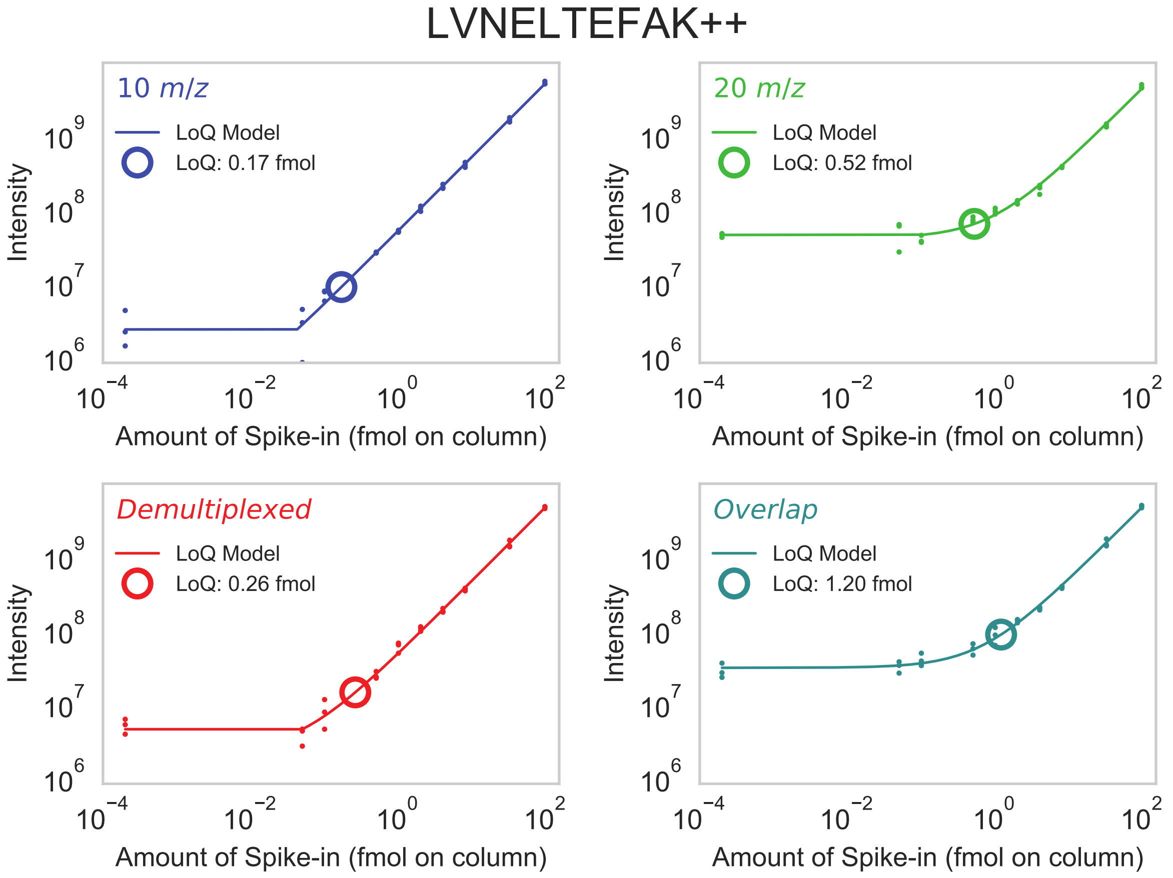

LVNELTEFAK++
10 m/z
LoQ Model
LoQ: 0.17 fmol
20 m/z
LoQ Model
LoQ: 0.52 fmol
Demultiplexed
LoQ Model
LoQ: 0.26 fmol
Overlap
LoQ Model
LoQ: 1.20 fmol
Intensity
Amount of Spike-in (fmol on column)

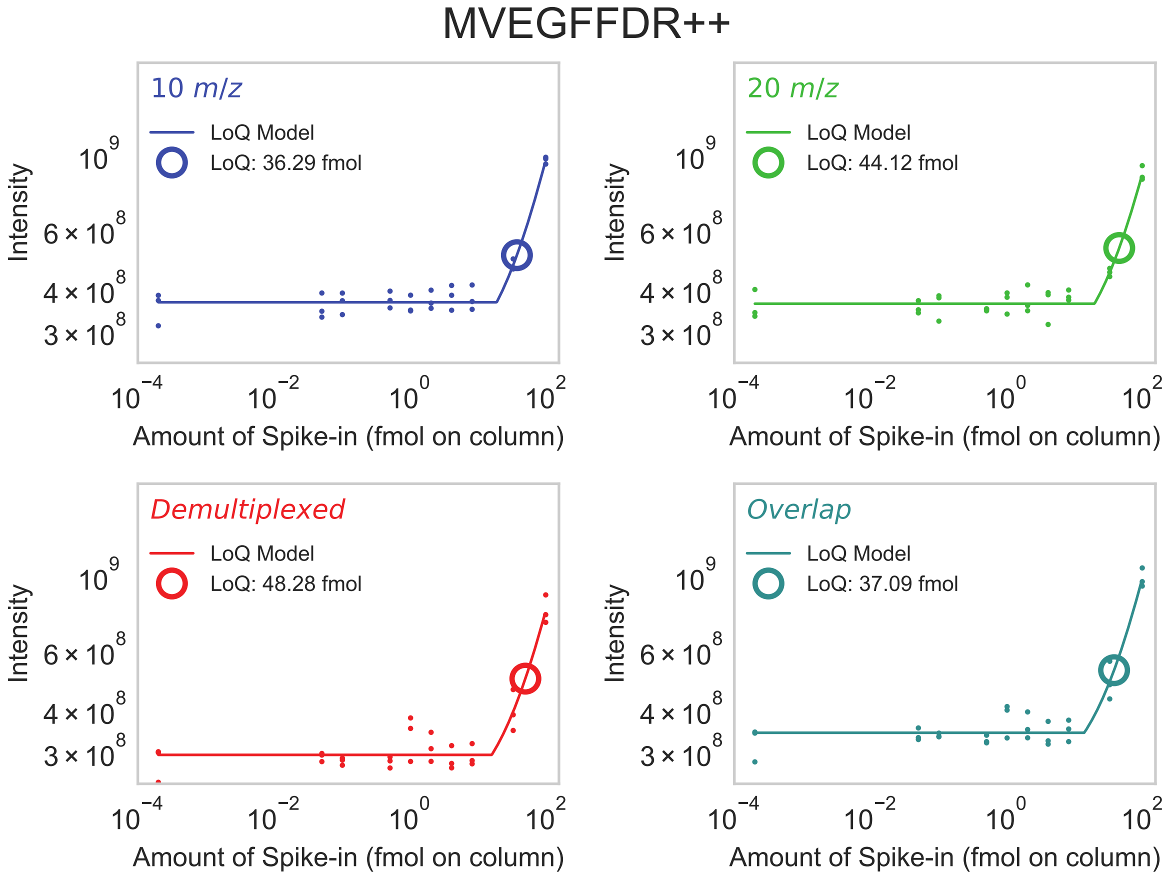

MVEGFFDR++
10 m/z
LoQ Model
LoQ: 36.29 fmol
20 m/z
LoQ Model
LoQ: 44.12 fmol
Demultiplexed
LoQ Model
LoQ: 48.28 fmol
Overlap
LoQ Model
LoQ: 37.09 fmol
Intensity
10^9
6×10^8
4×10^8
3×10^8
10^{-4}
10^{-2}
10^0
10^2
Amount of Spike-in (fmol on column)

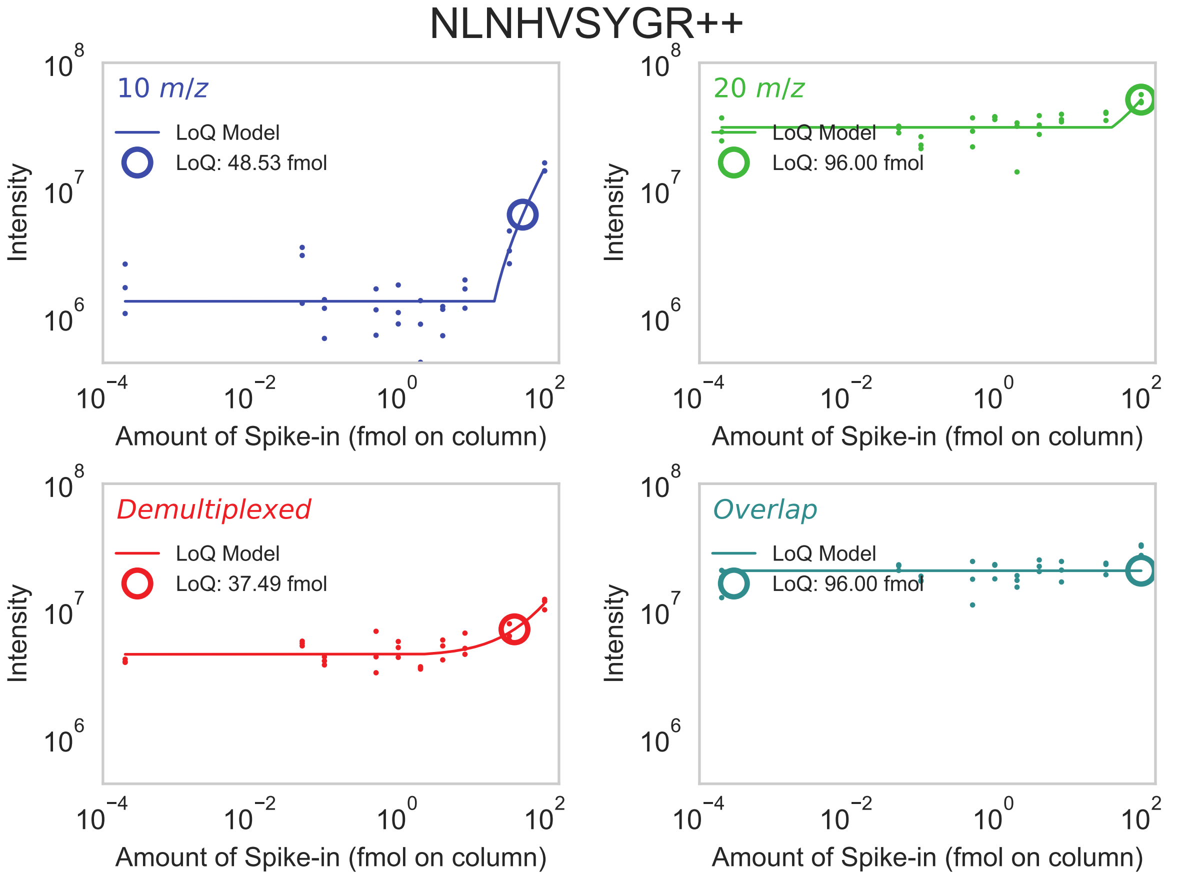

NLNHVSYGR++
10 m/z
LoQ Model
LoQ: 48.53 fmol
20 m/z
LoQ Model
LoQ: 96.00 fmol
Demultiplexed
LoQ Model
LoQ: 37.49 fmol
Overlap
LoQ Model
LoQ: 96.00 fmol
Intensity
Amount of Spike-in (fmol on column)

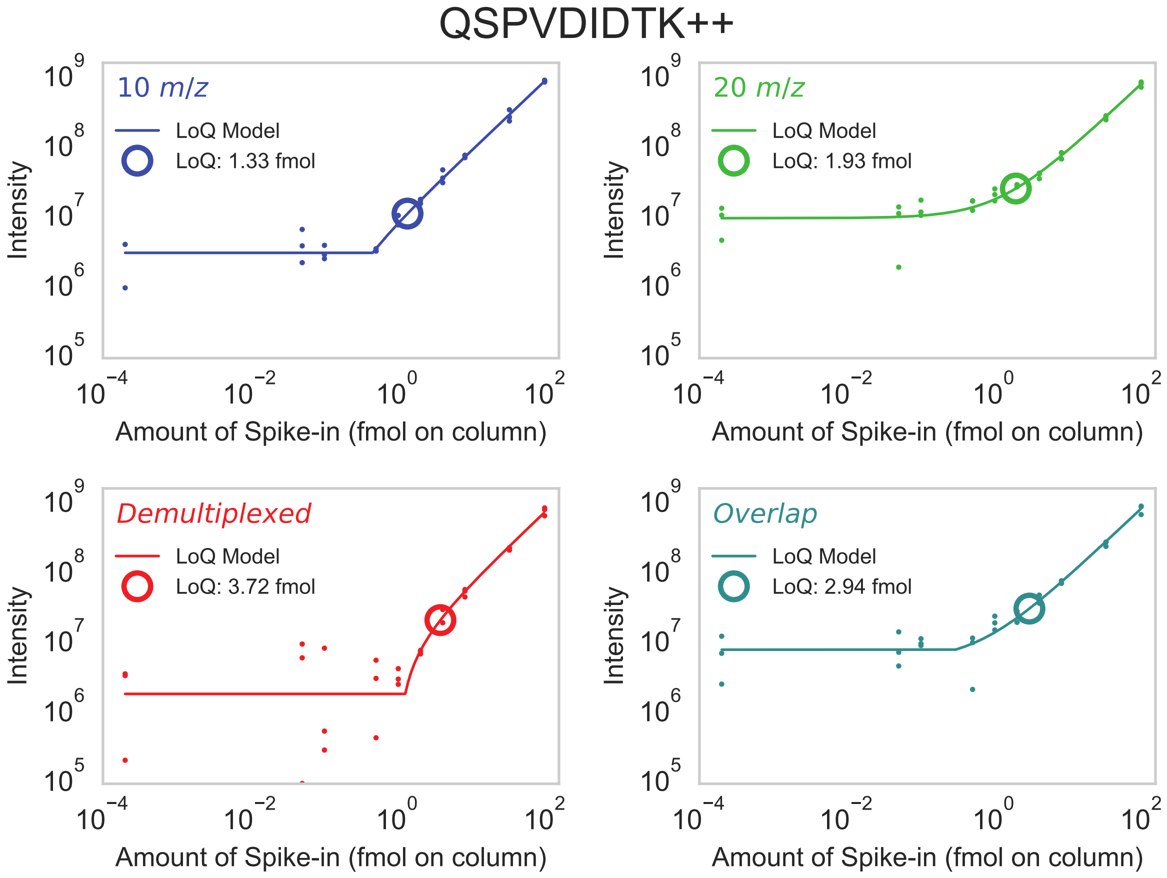
QSPVDIDTK++
10 m/z
LoQ Model
LoQ: 1.33 fmol
20 m/z
LoQ Model
LoQ: 1.93 fmol
Demultiplexed
LoQ Model
LoQ: 3.72 fmol
Overlap
LoQ Model
LoQ: 2.94 fmol
Intensity
Amount of Spike-in (fmol on column)
10^{5} 10^{6} 10^{7} 10^{8} 10^{9}
10^{-4} 10^{-2} 10^{0} 10^{2}

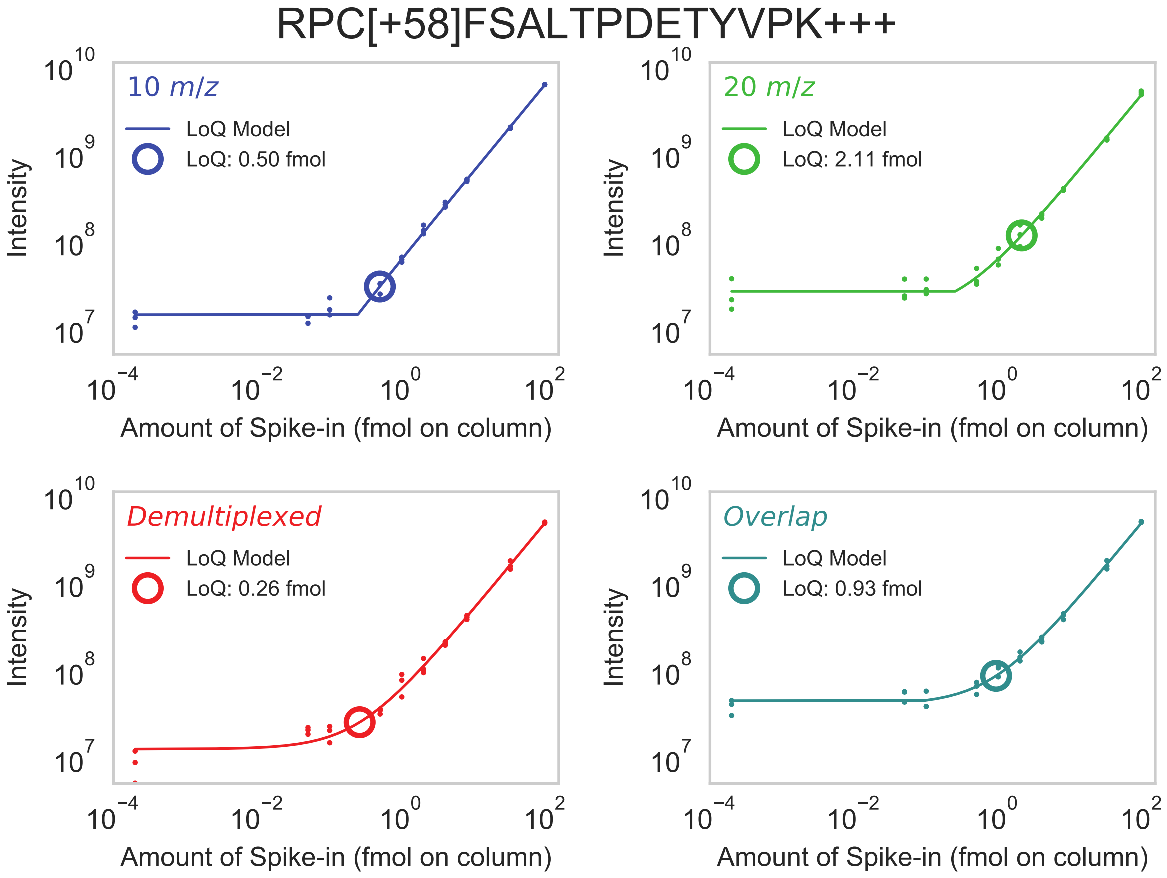
RPC[+58]FSALTPDETYVPK+++
10 m/z
LoQ Model
LoQ: 0.50 fmol
20 m/z
LoQ Model
LoQ: 2.11 fmol
Demultiplexed
LoQ Model
LoQ: 0.26 fmol
Overlap
LoQ Model
LoQ: 0.93 fmol
Intensity
Amount of Spike-in (fmol on column)

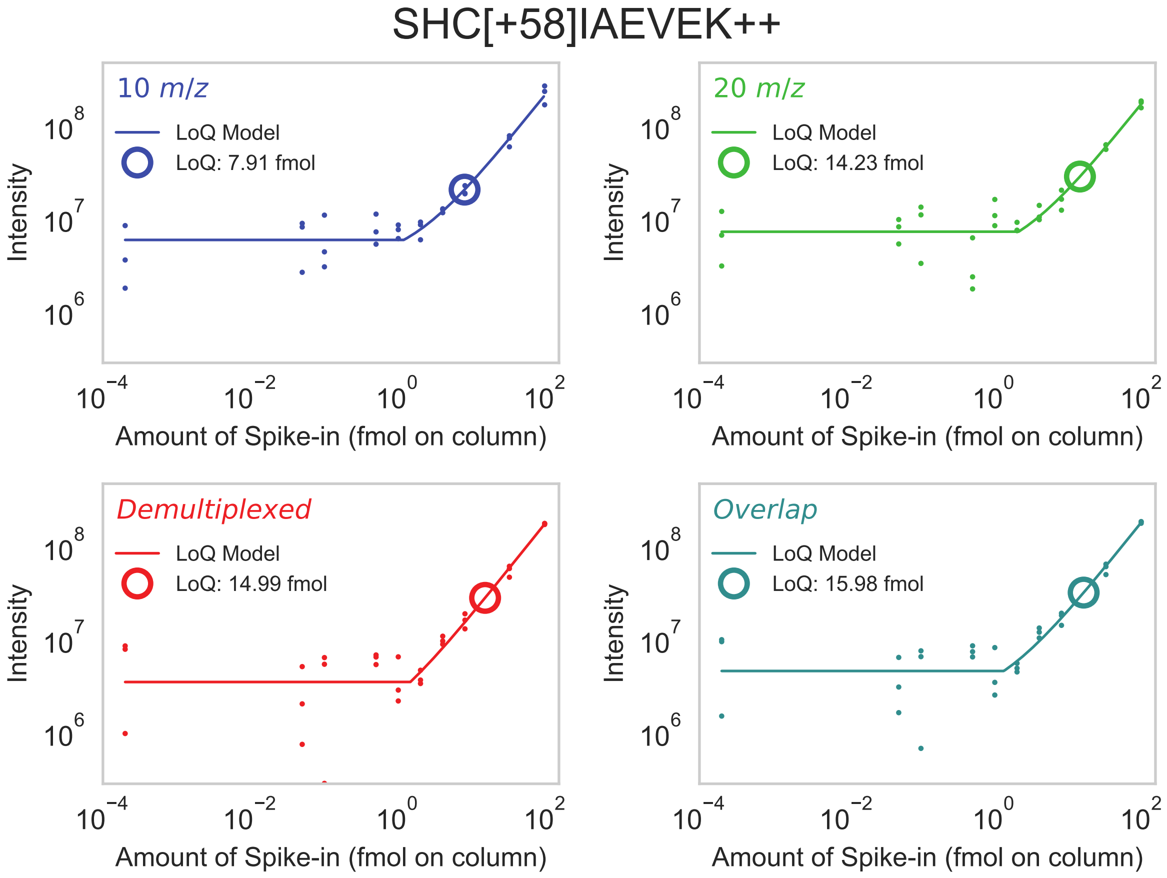
SHC[+58]IAEVEK++
10 m/z
LoQ Model
LoQ: 7.91 fmol
20 m/z
LoQ Model
LoQ: 14.23 fmol
Demultiplexed
LoQ Model
LoQ: 14.99 fmol
Overlap
LoQ Model
LoQ: 15.98 fmol
Intensity
Amount of Spike-in (fmol on column)

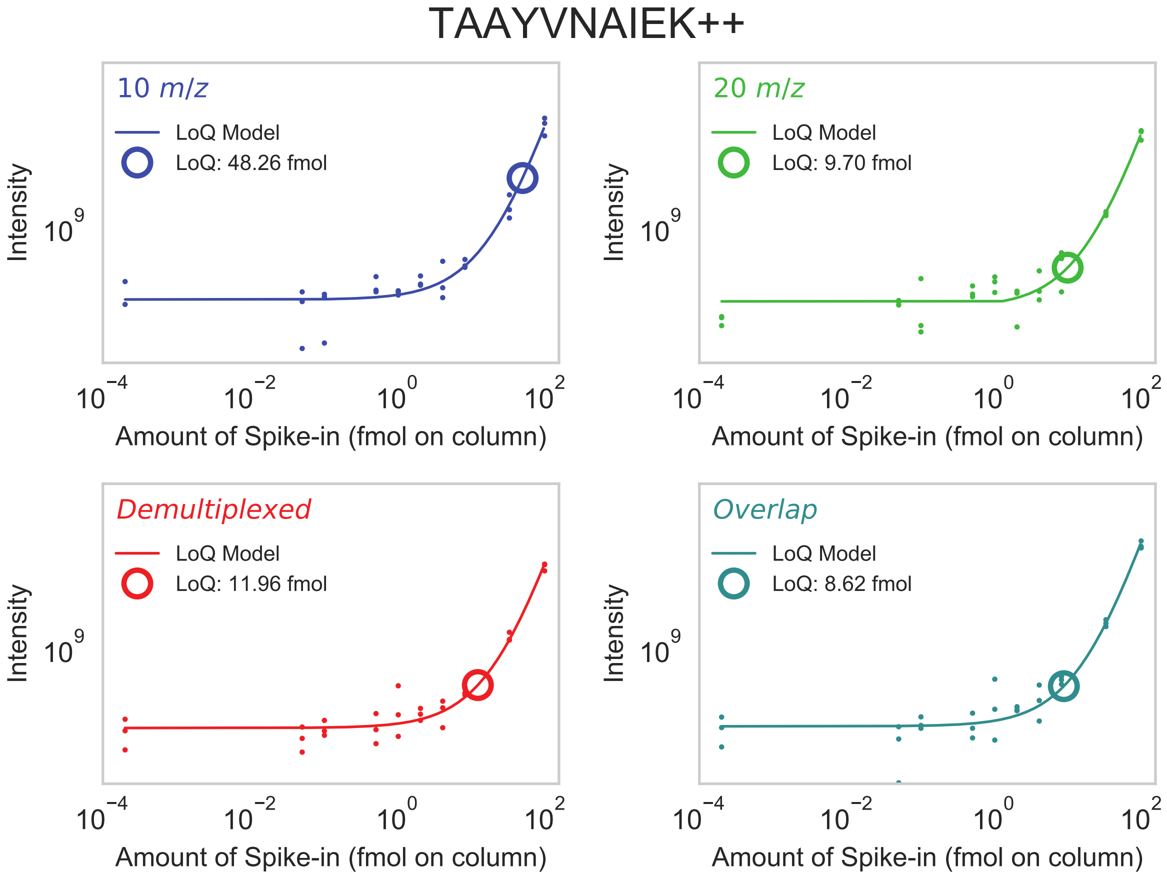
TAAYVNAIEK++
10 m/z
LoQ Model
LoQ: 48.26 fmol
20 m/z
LoQ Model
LoQ: 9.70 fmol
Demultiplexed
LoQ Model
LoQ: 11.96 fmol
Overlap
LoQ Model
LoQ: 8.62 fmol
Intensity
10^9
10^-4
10^-2
10^0
10^2
Amount of Spike-in (fmol on column)

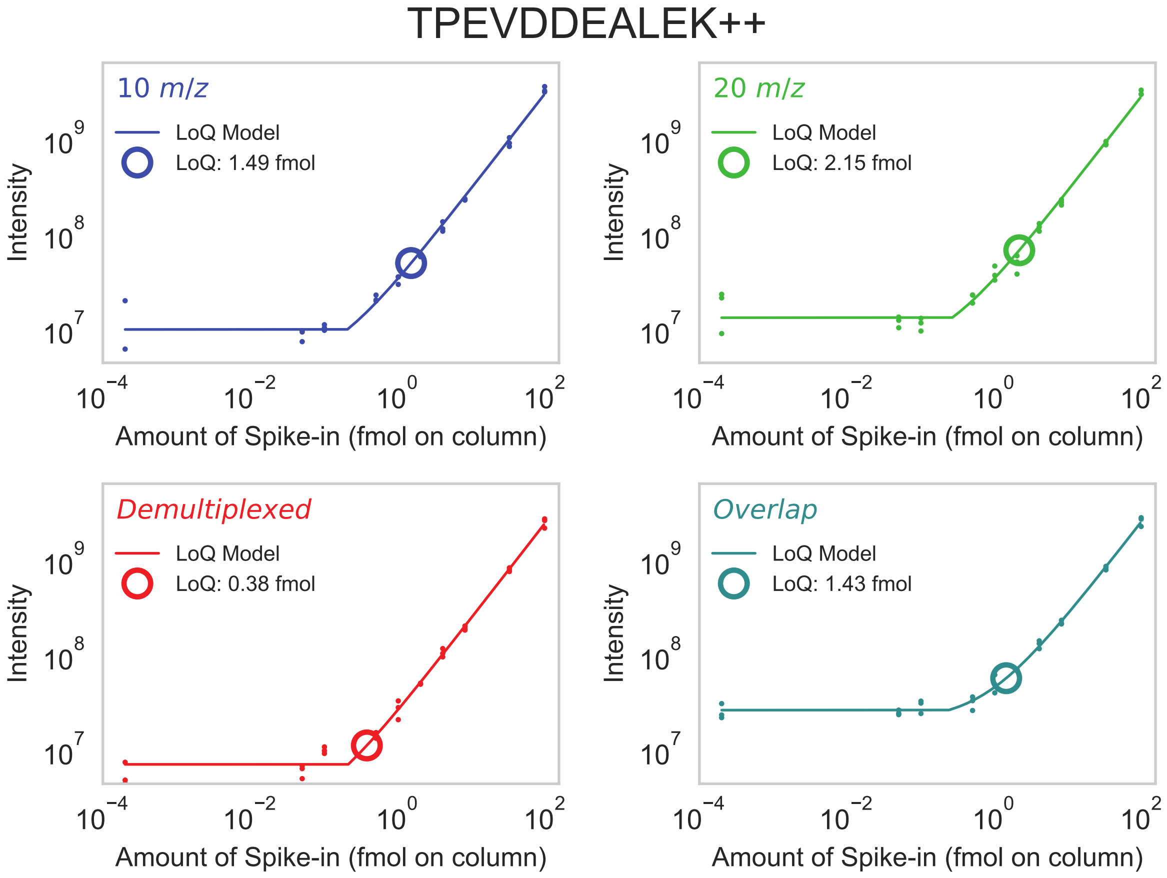
TPEVDDEALEK++
10 m/z
LoQ Model
LoQ: 1.49 fmol
20 m/z
LoQ Model
LoQ: 2.15 fmol
Demultiplexed
LoQ Model
LoQ: 0.38 fmol
Overlap
LoQ Model
LoQ: 1.43 fmol
Intensity
Amount of Spike-in (fmol on column)

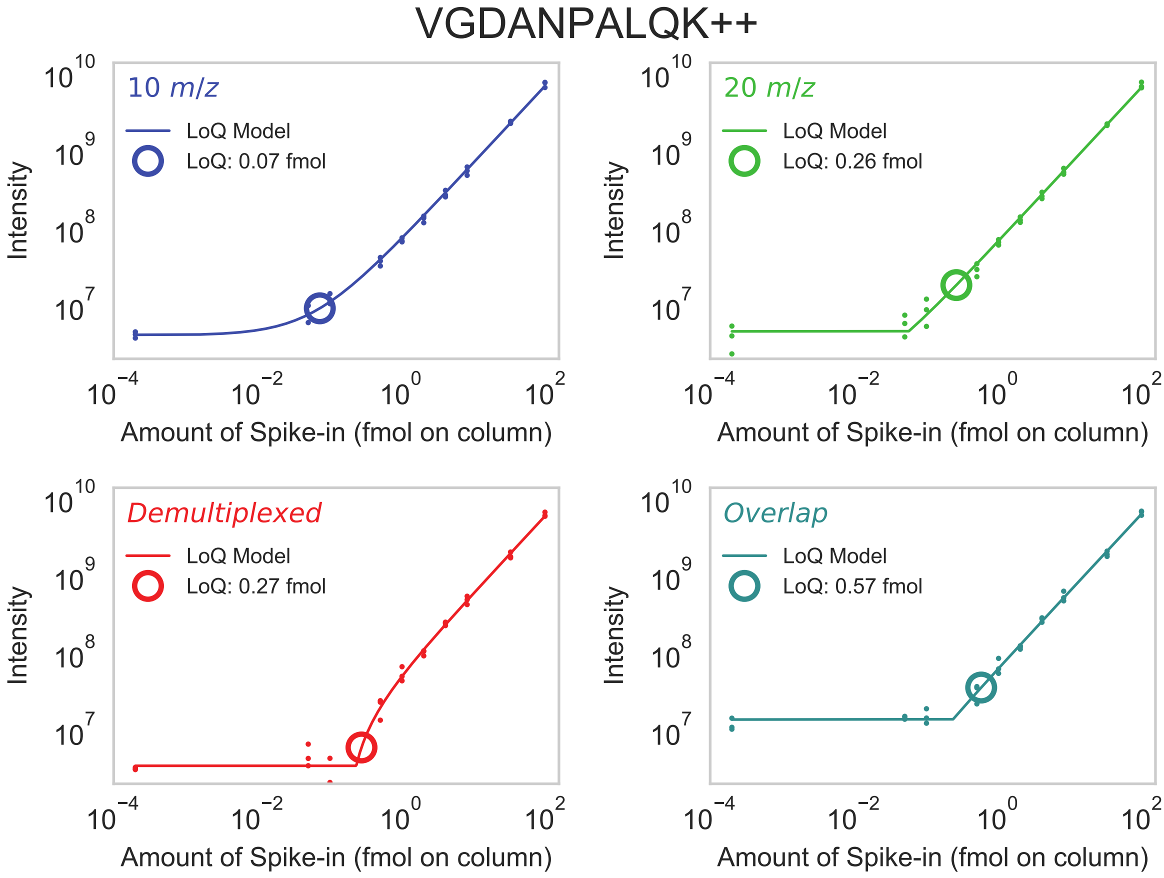
VGDANPALQK++
10 m/z
LoQ Model
LoQ: 0.07 fmol
20 m/z
LoQ Model
LoQ: 0.26 fmol
Demultiplexed
LoQ Model
LoQ: 0.27 fmol
Overlap
LoQ Model
LoQ: 0.57 fmol
Intensity
Amount of Spike-in (fmol on column)
10^{10}
10^{9}
10^{8}
10^{7}
10^{-4}
10^{-2}
10^{0}
10^{2}

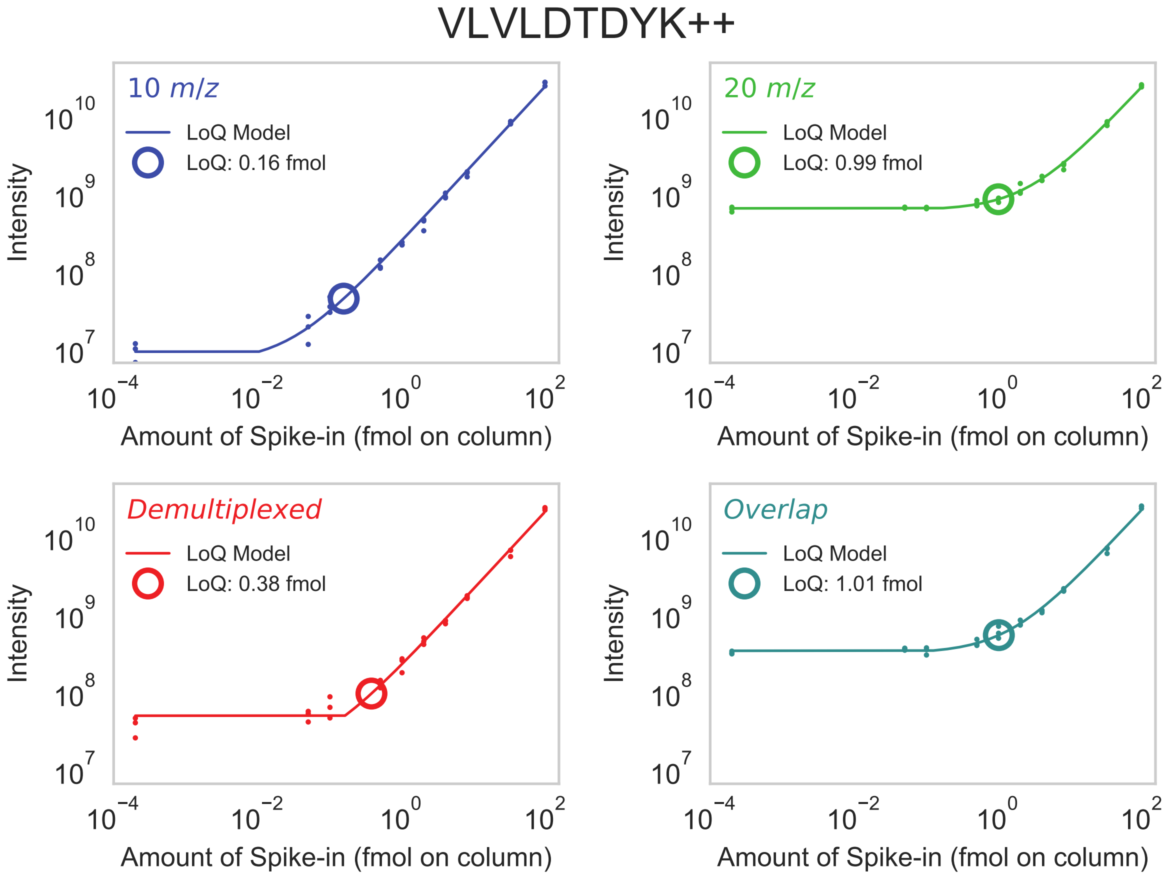

VLVLDTDYK++
10 m/z
LoQ Model
LoQ: 0.16 fmol
20 m/z
LoQ Model
LoQ: 0.99 fmol
Demultiplexed
LoQ Model
LoQ: 0.38 fmol
Overlap
LoQ Model
LoQ: 1.01 fmol
Intensity
Amount of Spike-in (fmol on column)

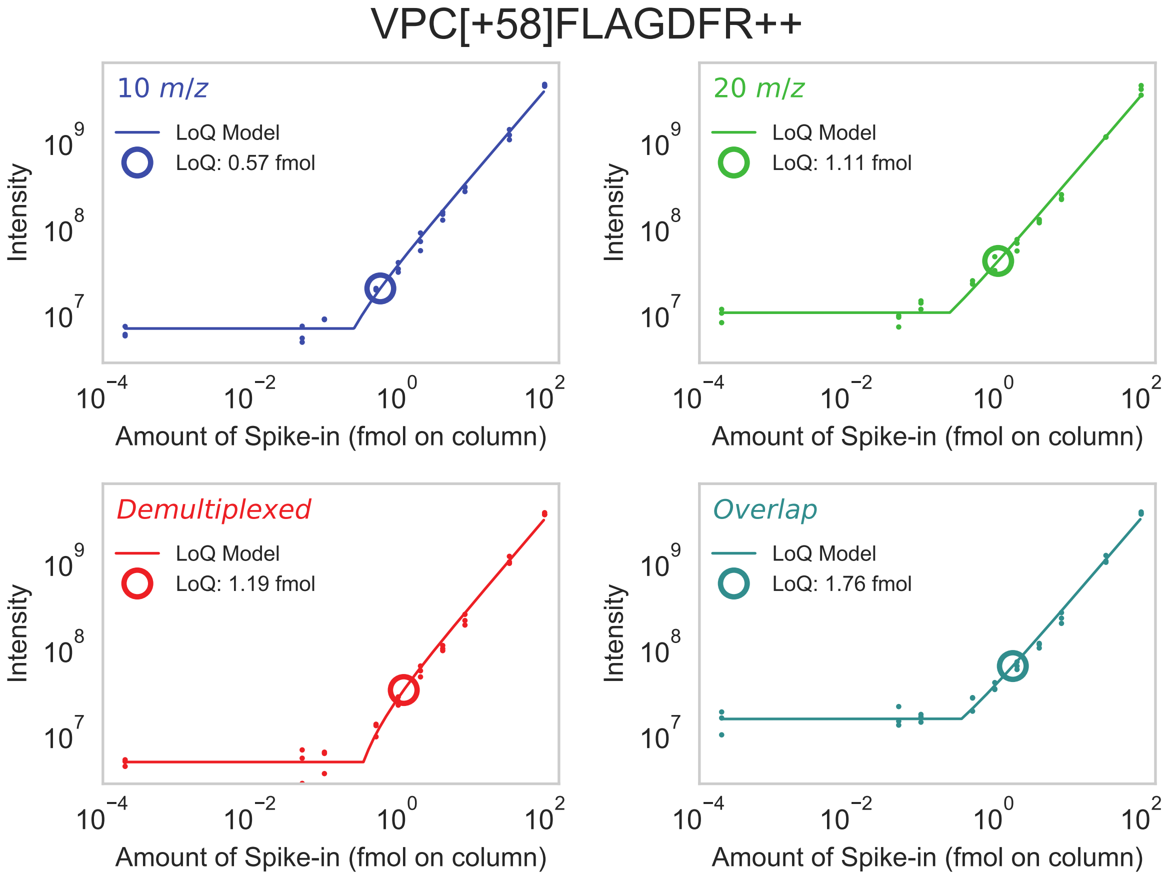
VPC[+58]FLAGDFR++
10 m/z
LoQ Model
LoQ: 0.57 fmol
20 m/z
LoQ Model
LoQ: 1.11 fmol
Demultiplexed
LoQ Model
LoQ: 1.19 fmol
Overlap
LoQ Model
LoQ: 1.76 fmol
Intensity
Amount of Spike-in (fmol on column)

VYNEAGVTFT++

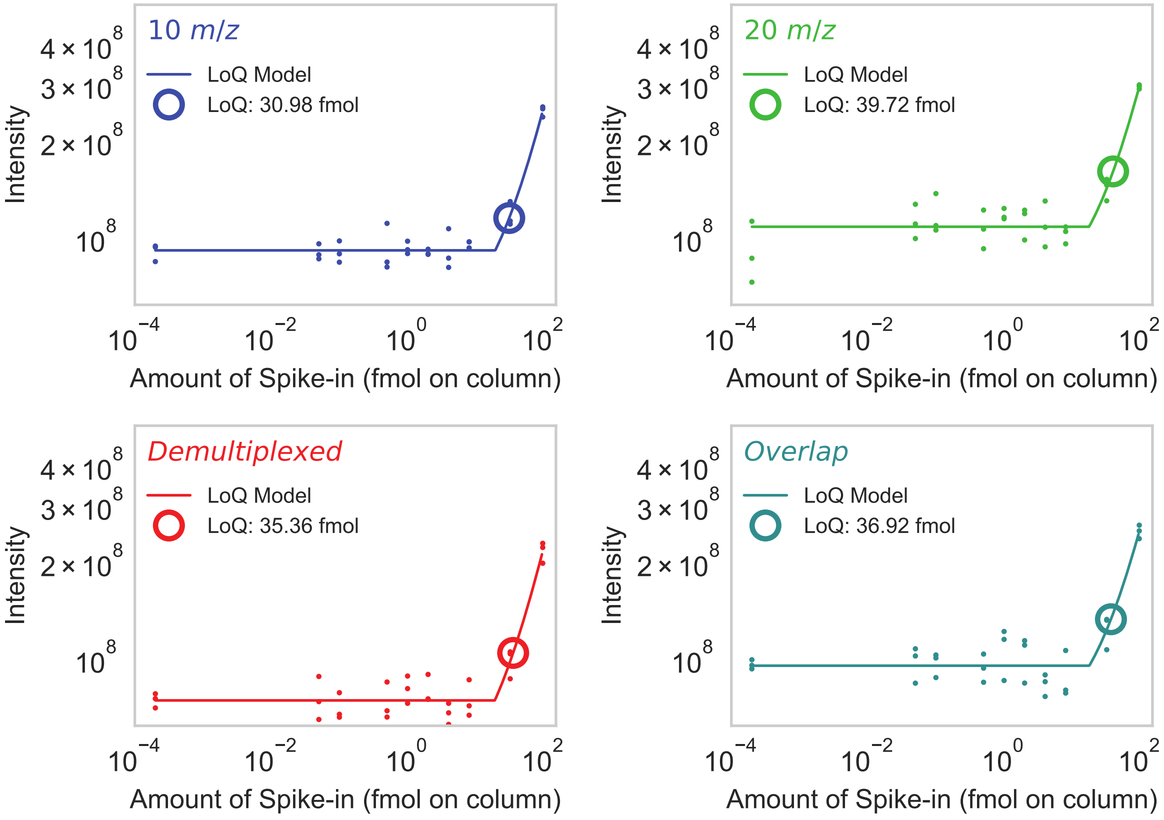

10 m/z
LoQ Model
LoQ: 30.98 fmol
20 m/z
LoQ Model
LoQ: 39.72 fmol
Demultiplexed
LoQ Model
LoQ: 35.36 fmol
Overlap
LoQ Model
LoQ: 36.92 fmol
Intensity
Amount of Spike-in (fmol on column)

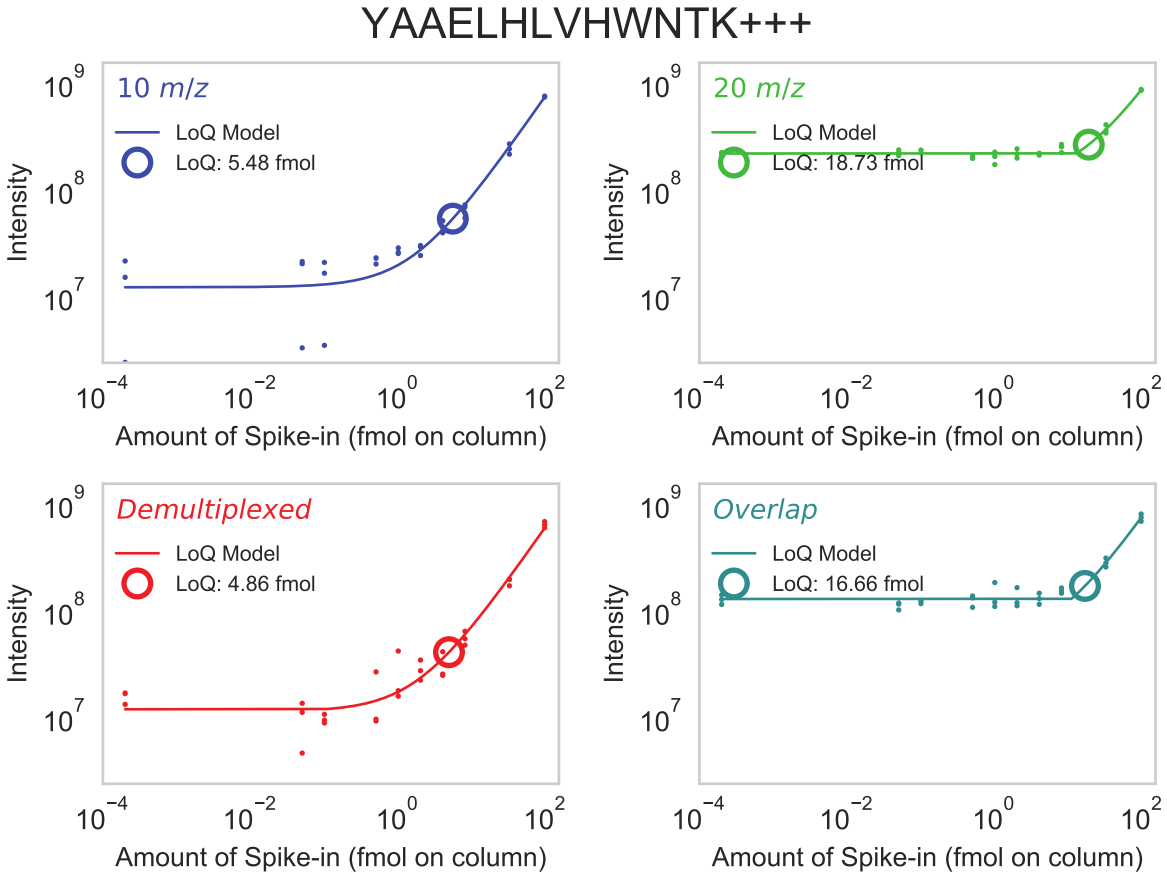

YAAELHLVHWNTK+++
10 m/z
LoQ Model
LoQ: 5.48 fmol
20 m/z
LoQ Model
LoQ: 18.73 fmol
Demultiplexed
LoQ Model
LoQ: 4.86 fmol
Overlap
LoQ Model
LoQ: 16.66 fmol
Intensity
Amount of Spike-in (fmol on column)

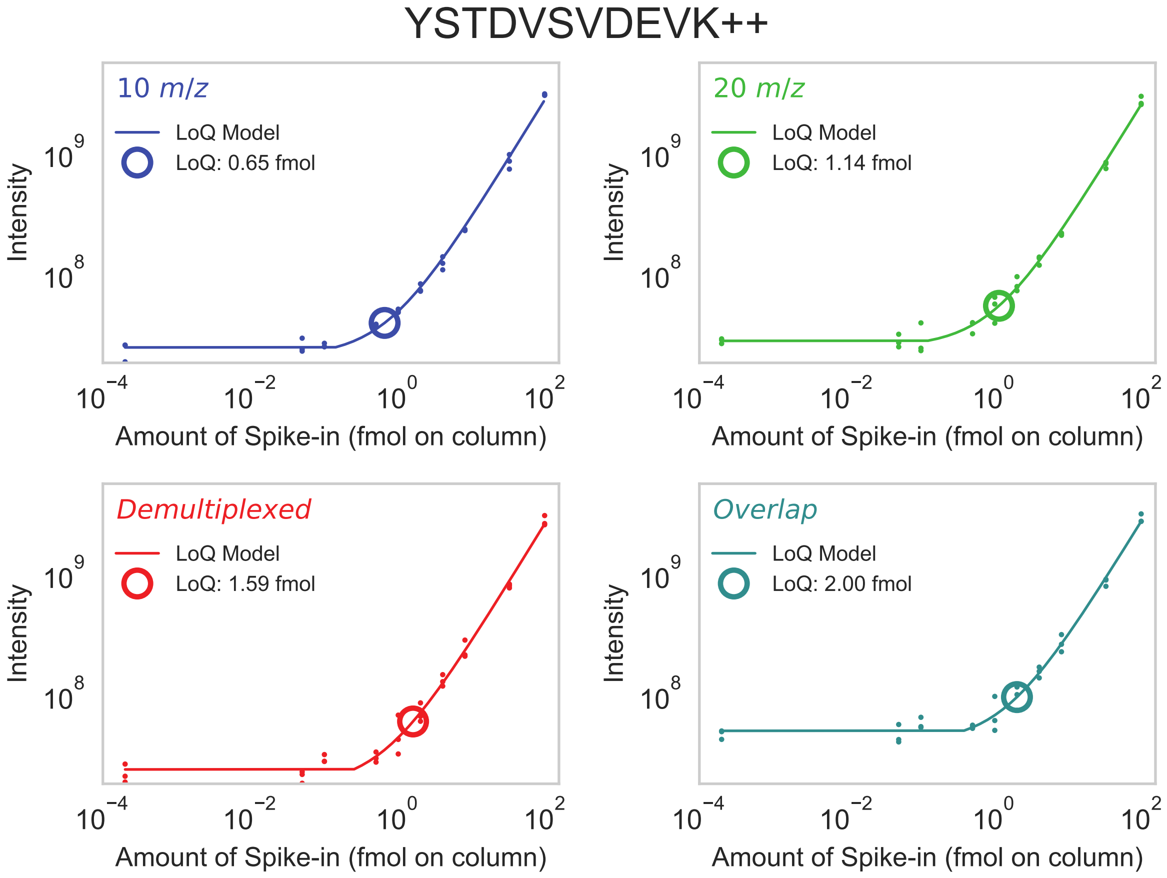
YSTDVSVDEVK++
10 m/z
LoQ Model
LoQ: 0.65 fmol
20 m/z
LoQ Model
LoQ: 1.14 fmol
Demultiplexed
LoQ Model
LoQ: 1.59 fmol
Overlap
LoQ Model
LoQ: 2.00 fmol
Intensity
Amount of Spike-in (fmol on column)
