# Supplementary material for: Improving Precursor Selectivity in Data-Independent Acquisition Using Overlapping Windows
Source: J Am Soc Mass Spectrom. 2019 Jan 22;30(4):669–84. doi: 10.1007/s13361-018-2122-8 (PMC6445824; doi:10.1007/s13361-018-2122-8)
Supplement: Supplementary file 12 — (PDF 143 kb) [file 13361_2018_2122_MOESM12_ESM.pdf]

Supplementary Note

1 Demultiplexing Algorithm

1.1 Defining the System of Equations

Our demultiplexing algorithm is a general procedure for assigning signals from wide but overlapping isolation windows to their appropriate sub-windows, and thus computationally increasing the precursor specificity of the collected data. The algorithm can be performed on any data collected with overlapping windows, regardless of the size of the windows, the length of the overlaps, or the configuration of the window cycles. For instance, the data collected in this manuscript uses 20 Th windows with alternating window cycles offset by 10 Th, but the algorithm could just as easily be applied to 25 Th windows with each cycle offset by 5 Th, or to data where each successive window, rather than window cycle, is successively offset by 5 Th.

Key to the algorithm is the notion of "demultiplexing sub-windows", the regions of overlap between windows to which signals can be uniquely assigned. Each scan of an isolation window can be approximated as a linear superposition of data from each of its component demultiplexing sub-windows. In the current manuscript, each 20 Th isolation window can be approximated as a superposition of data from the two demultiplexing sub-windows forming its left and right side. Different scans contain independent pairs of windows, enabling us to localize signals from the 20 Th windows to the appropriate 10 Th sub-window. The localization of signals from wide isolation windows to their appropriate demultiplexing sub-window is performed by solving a linear system of equations. For each scan s, and each transition t that we are interested in, we form the equation:

$$\mathbf{X}\vec{a} = \vec{y} \tag{1}$$

Here $\vec{y}$ is a vector containing the measured intensities of transition t for the N scans surrounding the scan of interest (i.e. scans number $s - N/2$ to $s + N/2 - 1$), where N is the total number of demultiplexing sub-windows (for the scheme in this manuscript, $N = 41$. $\vec{a}$ is a vector of the unknown intensities of the N demultiplexing sub-windows (which we are trying to solve for), in the current scheme these are the 41 10 Th sub-windows. $\mathbf{X}$ is the “design matrix”, such that the rows of $\mathbf{X}$ encode the set of demultiplexing sub-windows which are contained in a given isolation window scan ($\mathbf{X}$ contains a 1 wherever a sub-window lies in a window, and a 0 otherwise), and the columns of $\mathbf{X}$ are demultiplexing sub-windows.

Equation 1 describes how to solve the linear system of equations for a single transition. For purposes of practicality and computational efficiency, we typically lump together a number of transitions, so that the system of equations takes the form:

$$\mathbf{X}\mathbf{A} = \mathbf{Y} \tag{2}$$

where **A** and **Y** are now N x M matrices, where M is the number of transitions of interest. We emphasize that we never demultiplex the entire MS2 spectrum (which would be possible but computationally very expensive), and instead simply focus on the transitions of interest for a given scan (which are determined by which peptides we wish to extract chromatograms from).

A further improvement to the system of equations (2) can be made by taking advantage of an unusual property of the overlap pattern of scans, which is that the same combination of demultiplexing sub-windows repeats every 41 scans. For example, if scan 1 covers the 500-520 Th window (which i.e. consists of the 500-510 Th and 510-520 Th sub-windows), then scan 42 will cover exactly the same window (i.e. it is the corresponding scan in the next cycle), as will scans 83, 124, and so on. This is in contrast to the MSX method [2], in which combinations of isolation windows are generated randomly and are very unlikely to repeat. The repeating windows allow us to interpolate an estimate of the intensity in each window *all at the same time point* rather than evaluated across a 4 second duty cycle, which helps to reduce distortions caused by the non-constant shape of the peak elution profile. For example, the window which is scanned at $s = 1, 42, 83, 124, \ldots$ can be interpolated to scan $s = 100$ by building an interpolation function from the time points and intensities at $s = 1, 42, 83, 124, \ldots$:

$$(t_1, t_{42}, t_{83}, t_{124}), (I_1, I_{42}, I_{83}, I_{124}) \rightarrow S \tag{3}$$

$$I_{100} = S(t_{100}) \tag{4}$$

where I_s represents the intensity recorded at scan s and t_s represents the time at which scan s was taken. This process can then be repeated for every window to obtain a vector $\vec{y}$ that contains intensities for all the windows, all evaluated at the same scan time t_{100}.

S can be any interpolation function, but in this study we use cubic spline interpolation based on the 4 closest scans of a given window. We find that compared with earlier attempts to do demultiplexing without any interpolation, the spline-based interpolation produces smoother and more accurate chromatograms.

1.2 Solving the System of Equations

To solve the nonlinear least squares problem we use the Lawson-Hanson algorithm [2] implemented in Skyline (see "Skyline Implementation" in methods) and based upon the Matlab standard implementation of this algorithm. The Lawson-Hanson algorithm involves repeated inversions of combinations of rows of the design matrix (e.g. for a 40x40 matrix we might have to invert the 15x40 matrix containing rows 1 through 10, 16, 20, 38, and 39, as well as many other such submatrices), and for this reason the running time of the algorithm is typically $O(N^3ts)$ where N is the number of rows and columns of the design matrix, t is the number of transitions (we must solve a separate system of equations for each transition), and s is the number of scans (we must solve a separate system of equations for each scan). Our experiment involves $N = 41$ windows, $t \sim 1000$ transitions, and $s \sim 60,000$ scans, making the running time of the full demultiplexing algorithm infeasibly long. To reduce the running time we employ two computational shortcuts.

The first shortcut is based on the observation that the design matrix **X**is nearly diagonal, because every row consists of an adjacent set of 1's and the rest 0's. This is because every isolation windows consists of several adjacent demultiplexing sub windows. For instance, with 20 Th isolation windows and 10 Th overlaps, **X** has the tridiagonal form:

$$\mathbf{X} = \begin{bmatrix} 1 & 1 & 0 & 0 & \dots & 0 \\ 0 & 1 & 1 & 0 & \dots & 0 \\ 0 & 0 & \ddots & \ddots & 0 & 0 \\ \vdots & \vdots & 0 & 1 & 1 & 0 \\ 0 & 0 & \dots & 0 & 1 & 1 \end{bmatrix} \tag{5}$$

Thus, if we are interested in the 20th window (column) in this 41 row matrix, the windows near the edges are less likely to be important to the solution than the window near window 20, since they are much more indirectly connected to the 20th window. One can make a smaller approximation to the system $\mathbf{X}\vec{a} = \vec{y}$ that considers only the k rows and columns surrounding scan 20 and window 20, $20 - k/2$ to $20 + k/2 - 1$. In the extreme case, if $k = 2$, this reduces to inferring the intensity in a window simply by subtracting out neighboring scans. This extreme case gives poor results, but it turns out that k can be relatively small while still giving good results. We have empirically discovered that $k = 7$ is a good trade-off between accuracy and speed. With this approximation, the matrix $\mathbf{X}$ reduces to:

$$\mathbf{X} = \begin{bmatrix} 1 & 1 & 0 & 0 & 0 & 0 & 0 \\ 0 & 1 & 1 & 0 & 0 & 0 & 0 \\ 0 & 0 & 1 & 1 & 0 & 0 & 0 \\ 0 & 0 & 0 & 1 & 1 & 0 & 0 \\ 0 & 0 & 0 & 0 & 1 & 1 & 0 \\ 0 & 0 & 0 & 0 & 0 & 1 & 1 \\ 0 & 0 & 0 & 0 & 0 & 0 & 1 \end{bmatrix} \tag{6}$$

Given that N is now small, we can now employ the second trick, which involves caching the QR decomposition of every possible subset of rows of $\mathbf{X}$ for use in the Lawson-Hanson algorithm (as there are only $2^7 = 128$ such submatrices), eliminating the most time-intensive part of the computation. With these computational improvements, the algorithm can demultiplex the ~ 1000 transitions and $\sim 60,000$ scans in our runs in approximately 7 minutes, and in tests we find that even with 10,000 transitions the running time is only 30 minutes.

2 References

1. Egertson et al, Nature Methods, 2013.
2. Hanson and Lawson, Solving Least Squares Problems, 1974.
